# Integrin Beta 3 Regulates Cellular Senescence by Activating the TGF-β Pathway

**DOI:** 10.1016/j.celrep.2017.02.012

**Published:** 2017-03-07

**Authors:** Valentina Rapisarda, Michela Borghesan, Veronica Miguela, Vesela Encheva, Ambrosius P. Snijders, Amaia Lujambio, Ana O’Loghlen

**Affiliations:** 1 Epigenetics & Cellular Senescence Group, Blizard Institute, Barts and The London School of Medicine and Dentistry, Queen Mary University of London, 4 Newark Street, London E1 2AT, UK; 2 Department of Oncological Sciences, Icahn School of Medicine at Mount Sinai, 1470 Madison Avenue, New York, NY 10029, USA; 3 Protein Analysis and Proteomics Group, The Francis Crick Institute, South Mimms EN6 3LD, UK

**Keywords:** senescence, integrin, aging, CBX7, ITGB3, β3, SASP, TGFβ, cilengitide, Palbociclib

## Abstract

Cellular senescence is an important in vivo mechanism that prevents the propagation of damaged cells. However, the precise mechanisms regulating senescence are not well characterized. Here, we find that *ITGB3 (*integrin beta 3 or β3) is regulated by the Polycomb protein CBX7. β3 expression accelerates the onset of senescence in human primary fibroblasts by activating the transforming growth factor β (TGF-β) pathway in a cell-autonomous and non-cell-autonomous manner. β3 levels are dynamically increased during oncogene-induced senescence (OIS) through CBX7 Polycomb regulation, and downregulation of β3 levels overrides OIS and therapy-induced senescence (TIS), independently of its ligand-binding activity. Moreover, cilengitide, an αvβ3 antagonist, has the ability to block the senescence-associated secretory phenotype (SASP) without affecting proliferation. Finally, we show an increase in β3 levels in a subset of tissues during aging. Altogether, our data show that integrin β3 subunit is a marker and regulator of senescence.

## Highlights

• *ITGB3* (integrin β3) is regulated by the Polycomb protein CBX7
• β3 regulates senescence by activating TGF-β in a paracrine and autocrine fashion
• β3 is highly expressed in OIS and induces senescence via ligand-independent pathway
• There is a positive correlation between β3 levels and aging in different tissues

## Graphical Abstract

**Figure:**
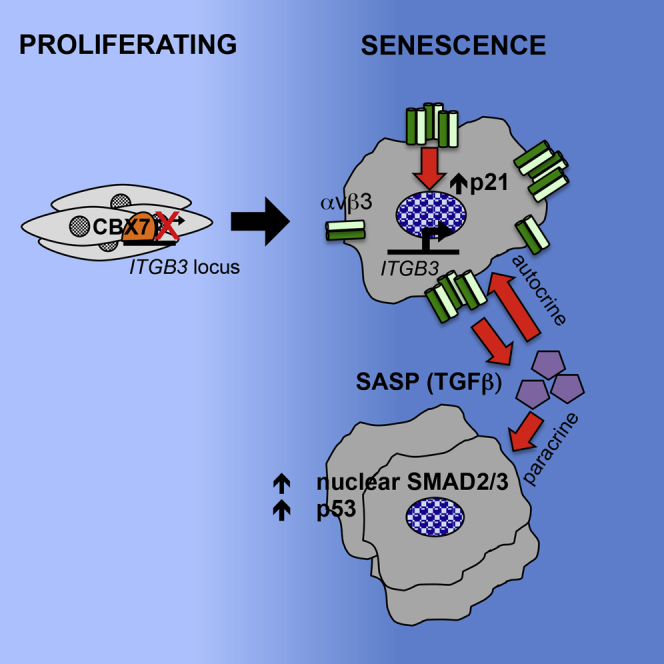

## Teaser

Rapisarda et al. show that integrin β3 subunit expression induces senescence by activating TGF-β, while β3 knockdown overcomes senescence. β3 is dynamically upregulated in OIS and has ligand-independent activity. They also find a positive correlation between β3 levels and aging in a subset of tissues.

## Introduction

Cellular senescence is characterized by a proliferative arrest induced to prevent the propagation of damaged cells in a tissue. This arrest is mainly driven by the activation of two important pathways, p53/p21^CIP^ and RB/p16^INK4A^. The senescence program can be triggered by a number of stressors, like the activation of oncogenes (termed oncogene-induced senescence; OIS), drug treatment (therapy-induced senescence; TIS) or deregulation of Polycomb Repressive Complex 1 (PRC1) proteins, including the polycomb protein chromobox 7 (CBX7) (Gil and O’Loghlen, 2014, Salama et al., 2014). Although arrested, senescent cells are metabolically and transcriptionally functional, and they actively communicate with their surroundings (Pérez-Mancera et al., 2014). In fact, senescent cells secrete an array of inflammatory proteins, growth factors, and metalloproteases that collectively constitute the SASP (senescence-associated secretory phenotype). The SASP recruits the immune system in order to eliminate senescent cells and induces changes in the extracellular matrix (ECM), thus facilitating tissue homeostasis and regeneration. The presence of senescent cells has been found in vivo in preneoplastic lesions (Muñoz-Espín and Serrano, 2014), in wound healing (Jun and Lau, 2010), during embryonic development (Muñoz-Espín et al., 2013, Storer et al., 2013), and in different tissues throughout aging (Baker et al., 2016, Krishnamurthy et al., 2004, Ressler et al., 2006). Interestingly, a recent study has demonstrated that p16^INK4A^-positive cells accumulate during aging and contribute to age-related dysfunctions in different tissues. Thus, the elimination of senescent cells reverses the aging phenotype and stimulates tissue regeneration, demonstrating that the activation of senescence is a direct cause of aging and opening avenues for targeting senescent cells as a therapy to extend healthy lifespan (Baker et al., 2016).

Senescence activation is governed by intracellular and extracellular signals and highly depends on the interaction of the cells with ligands in the ECM (Gutiérrez-Fernández et al., 2015, Jun and Lau, 2010, Parrinello et al., 2005). The most common ECM-receptor interaction proteins are integrins, which are heterodimeric cell-surface transmembrane receptors that provide cellular adhesion (Hynes, 2002). Upon ligand binding, intracellular proteins are recruited to clusters of integrin heterodimers in the plasma membrane, forming focal adhesion (FA) complexes, which mediate downstream signals to the cell. Integrin signaling affects numerous cellular processes, including cell adhesion, migration, proliferation, survival, and differentiation (Seguin et al., 2015) and, thus, has key roles during development, immune responses, and different pathologies such as cancer (Legate et al., 2009). Interestingly, integrins can also mediate signaling cascades independent of ligand binding. In fact, integrin αvβ3 can induce apoptosis (Stupack et al., 2001), tumor progression (Desgrosellier et al., 2009), and tumor stemness (Seguin et al., 2014) in an anchorage-independent manner.

Here, we establish integrin beta 3 (β3) subunit as a marker and regulator of senescence. Our findings highlight the importance of the β3 subunit signaling in regulating senescence by activating the transforming growth factor β (TGF-β) pathway in an autocrine and paracrine fashion. We found that β3 levels are dynamically upregulated during OIS, by CBX7 transcriptional regulation, and that β3 regulates OIS independently of its ligand-binding activity. Importantly, an αvβ3 antagonist exerts dual activity by regulating interleukin (IL)-6/IL-8 secretion but not the growth arrest during OIS. Additionally, we found an increase in the levels of β3 during aging in a subset of mouse tissue and human samples, where the manipulation of β3 levels in fibroblasts derived from old human donors overcomes the accumulation of different markers of senescence and aging. Our results demonstrate the importance of cellular adhesion during senescence and identify integrins as potential therapeutic targets during early carcinogenesis and aging.

## Results

### SILAC Screen Identifies Proteins Grouped in the ECM-Receptor Interaction Pathway as Putative Regulators of Senescence

**Figure 1:**
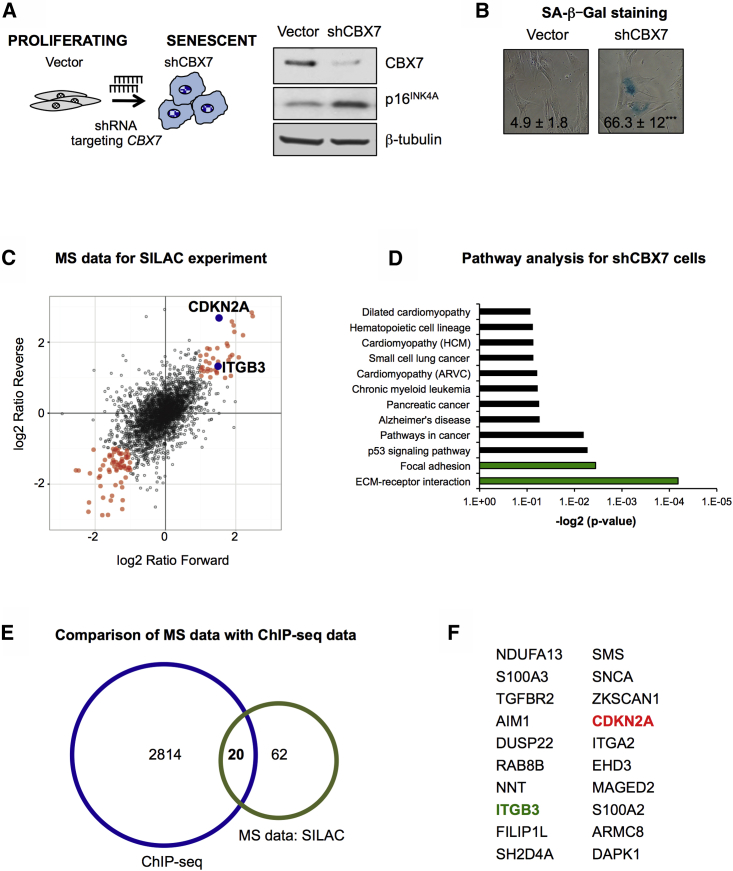
SILAC Screen Identifies Putative Regulators of Senescence (A) Left panel: schematic representation of the senescence model used in the SILAC screen. Human primary breast fibroblasts (BFs) were transduced with a lentivirus harboring an shRNA targeting *CBX7* (shCBX7). Right panel: immunoblot showing CBX7 knockdown efficiency and an increase in p16^INK4A^ protein levels. β-tubulin is used as loading control. (B) Senescence induced upon shCBX7 is shown by an increase in the percentage of cells staining positive for SA-β-galactosidase (SA-β-Gal) activity. Quantification of two to three independent experiments is shown. (C) Scatterplot of mass spectrometry (MS) results from both forward and reverse SILAC experiments. A 2-fold difference in expression upon shCBX7 is indicated with orange circles, outlining CDKN2A and ITGB3 in blue. Gray circles represent unchanged proteins. (D) Pathway analyses (KEGG) show that proteins with a 2-fold difference in expression fall within the categories of the extracellular matrix (ECM)-interacting and FA pathways. (E) Comparison of the proteins significantly deregulated in the SILAC experiment with a published dataset for genes regulated by CBX proteins in human diploid fibroblasts (Pemberton et al., 2014). (F) List of 20 proteins in the SILAC screen whose genes could be regulated by CBX proteins, highlighting CDKN2A (red) and ITGB3 (green).

We have previously shown that CBX7 loss of function by short hairpin RNA (shRNA) in primary cells induces cellular senescence (Gil and O’Loghlen, 2014, O’Loghlen et al., 2015a, O’Loghlen et al., 2015b) (Figures 1A and 1B). This has been primarily attributed to the derepression of the *CDKN2A* locus, which encodes the cell-cycle inhibitor p16^INK4A^. As PRC1 targets multiple genes, and we see additional markers of senescence present upon *CBX7* knockdown (Figures 1B and S1A), we hypothesized that other unknown regulators could be inducing senescence in this model. To this end, we performed a quantitative proteomic analysis to determine changes occurring upon *CBX7* knockdown in human primary fibroblasts, taking advantage of the SILAC (stable isotope labeling by amino acids in culture) technology. Human primary breast fibroblasts (BFs) transduced with an shRNA targeting *CBX7* (shCBX7) were grown in media supplemented with “heavy” (forward experiment)- or “light” (reverse experiment)-labeled amino acids and compared with BFs expressing an empty vector (Figure S1B). Combination of the results from both forward and reverse experiments show 82 proteins with a 2-fold expression difference in both experiments, including CDKN2A (Figure 1C). Annotation of differentially expressed proteins into functional pathways (Kyoto Encyclopedia of Genes and Genomes; KEGG) shows the ECM-receptor-interacting and focal adhesion pathways upregulated upon *CBX7* knockdown (Figure 1D).

**Figure 2:**
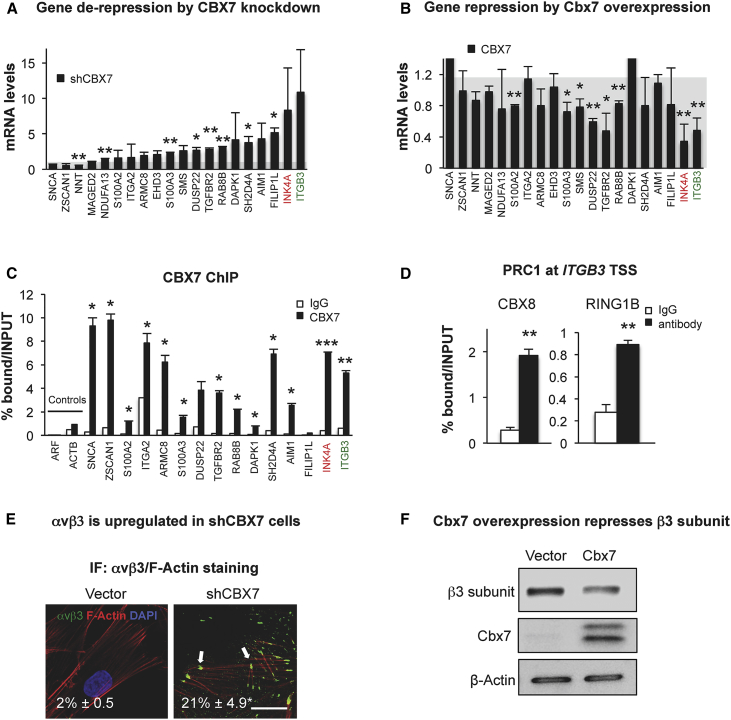
The Genes Encoding the Proteins Found in the SILAC Screen Are Regulated by CBX7 (A) qPCR analyses show the relative mRNA levels of the selected genes upon shCBX7. *INK4A* is highlighted in red as a known CBX7-regulated gene, and *ITGB3* is highlighted in green as a potentially new gene regulated by CBX7. Data are normalized to the control, shown as a gray shade, and represent the mean ± SD of two independent experiments. (B) Overexpression of Cbx7 reduces the expression of its target genes. Relative mRNA levels are shown by qPCR. Data are normalized to the control, shown as a gray shade, and represent the mean ± SD of two independent experiments. (C) ChIP for endogenous CBX7 (black bars) shows enrichment at the transcription start site (TSS) of its target genes, in comparison with immunoglobulin G (IgG) control (white bars). There is no CBX7 enrichment at the TSS of non-PRC1 target genes (Controls): *ARF* (encoding p14^ARF^) and *ACTB* (β-actin). Data represent the mean ± SD of a representative experiment. (D) ChIP for other PRC1 proteins (CBX8 and RING1B) show enrichment at the TSS of *ITGB3*, in comparison with IgG (white bars). A representative experiment is shown. (E) Representative images showing integrin αvβ3 (green) and F-actin (red) staining in fibroblasts expressing empty vector or shCBX7. The formation of αvβ3-stained FA complexes can be observed only in cells harboring shCBX7 (white arrows). The quantification indicates the percentage of cells positive for αvβ3 staining ± SD (three to five independent experiments). Scale bar, 20 μm. (F) A representative blot for BFs overexpressing Cbx7 shows reduced levels of endogenous β3 subunit and mouse Cbx7 overexpression levels. β-actin is used as loading control. ^∗^p < 0.05; ^∗∗^p < 0.01; ^∗∗∗^p < 0.001.

As the SILAC screen was performed in a CBX7-depleted background, we hypothesized that the genes encoding the proteins found in the SILAC screen could be regulated by CBX7. Thus, we compared the SILAC data with a published genome-wide binding profile for CBX proteins (chromatin immunoprecipitation sequencing; ChIP-seq) in human fibroblasts (Pemberton et al., 2014). We found 20 proteins whose genes are potential targets for CBX proteins, including *CDKN2A* (Figures 1E and 1F). In fact, knockdown of *CBX7* led to more than 2-fold upregulation of the mRNA levels of the majority of the 20 genes, as shown by qPCR (Figures 2A and S1C), while overexpression of murine Cbx7 resulted in gene silencing and transcriptional repression (Figures 2B and S1D). This was repeated using a different strain of human fibroblast, IMR-90 (Figure S1E). To further confirm that these genes are regulated by CBX7, we performed ChIP for endogenous CBX7 in BFs and analyzed the enrichment of CBX7 at the transcription start site (TSS) of the 20 genes. Our data show enrichment for CBX7 at the TSS of the analyzed genes, including *INK4A (*encoding p16^INK4A^), but not the negative controls *ARF* (encoding p14^ARF^) or *ACTB* (encoding β-actin) (Figure 2C).

### *ITGB3* Is Regulated by PRC1

Among the potential inducers of senescence regulated by CBX7, we focused on *ITGB3* (which encodes for the integrin β3 subunit) because: (1) it is a component of the two most representative pathways upon *CBX7* knockdown in the SILAC; (2) we found CBX7 at the TSS of *ITGB3* in the ChIP-seq dataset; and (3) it is the gene that is most upregulated upon *CBX7* knockdown. Interestingly, two additional PRC1 proteins, CBX8 and RING1B, were also found at the TSS of the *ITGB3* locus (Figure 2D), further supporting the regulation of *ITGB3* by PRC1. To confirm whether the latter changes at the mRNA level correlate with protein levels, we checked the levels of the integrin heterodimer αvβ3 by immunofluorescence (IF) (Figure 2E) and the β3 subunit by immunoblot upon *CBX7* knockdown (Figure S1F) or Cbx7 ectopic expression (Figure 2F). Importantly, we observed that shCBX7 increases the number of cells presenting αvβ3-stained FA complexes by IF (Figure 2E). The regulation of β3 protein levels by CBX7 was also confirmed in IMR-90 fibroblasts (Figure S1G). Altogether, these data show that the *ITGB3* locus is regulated by the Polycomb protein CBX7 in human primary fibroblasts.

### ITGB3 Induction of Senescence Is Dependent on the p53/p21^CIP^ Pathway

**Figure 3:**
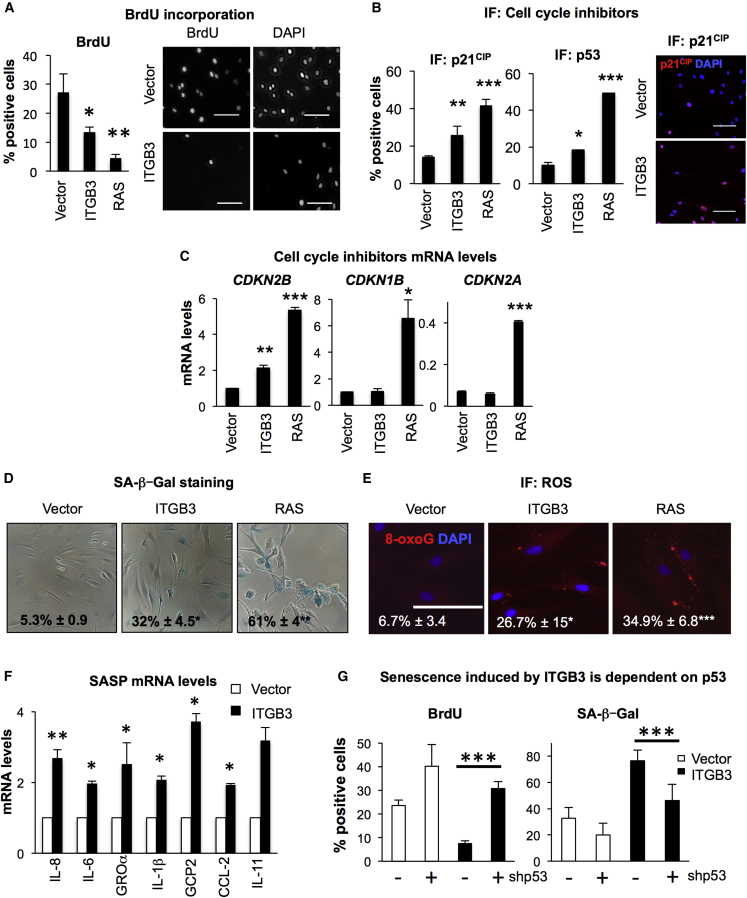
ITGB3 Ectopic Expression Induces Senescence via p21^CIP^/p53 Pathway (A–F) Overexpression of a retroviral construct encoding ITGB3 in BFs induces senescence. H-Ras^G12V^ (RAS) is used as a positive control for inducing senescence. (A) We show a reduction in proliferation in BFs expressing ITGB3 by measuring the percentage of cells incorporating BrdU (left panel: quantification levels; right panel: representative pictures). Proliferation was assessed 4–5 days after plating. BFs expressing ITGB3 show (B) an increase in p21^CIP^ and p53 protein levels by IF 4–5 days after plating (left panel: percentage of cells stained positive for p21^CIP^ and p53; right panel: representative pictures for p21^CIP^ staining) and (C) an increase in *CDKN2B* (encoding p15^INK4B^) mRNA levels by qPCR. No changes were observed in *CDKN1B* (encoding p27^KIP1^) or *CDKN2A* (p16^INK4A^). (D) Expression of ITGB3 also induced an increase in senescence-associated β-galactosidase activity (SA-β-Gal). Data represent the percentage of cells staining positive for SA-β-Gal ± SD. Staining was performed 7–10 days after plating; (E) an increase in the levels of ROS, measured by 8-oxoG staining, and (F) a mild increase of the mRNA levels of different SASP by qPCR. Cells were subjected to analysis (either by IF or qPCR) 4–5 days after plating. (G) An shRNA against p53 (shp53) prevents the activation of senescence induced by ITGB3 ectopic expression, as shown by the reversion in the percentage of BFs incorporating BrdU induced by ITGB3 (left graph) and the decrease in SA-β-Gal activity (right graph). BrdU was added 24 hr prior to fixing the cells for IF. Data represent the mean ± SD of more than two independent experiments. Scale bars, 100 μm. ^∗^p < 0.05; ^∗∗^p < 0.01; ^∗∗∗^p < 0.001.

The aforementioned findings suggest that ITGB3 could be a regulator of cellular senescence. Indeed, expression of a retroviral vector encoding ITGB3 in BFs reduces their proliferation rate, quantified by measuring the percentage of cells incorporating bromodeoxyuridine (BrdU) (Figure 3A). A retroviral construct expressing the oncogene H-Ras^G12V^ (RAS) was used as a positive control (Serrano et al., 1997). Concomitant with the growth arrest, we observed an increase in the protein levels of the cell-cycle inhibitors p21^CIP^ and p53 by IF (Figure 3B) and *CDKN2B* mRNA levels by qPCR, with no changes observed in *CDKN2A* or *CDKN1B* (Figure 3C). Consistent with the activation of senescence, ITGB3 expression led to an increase in the number of cells staining positive for senescence-associated β-galactosidase (SA-β-Gal) activity (Figure 3D), an accumulation of reactive oxygen species (ROS) (Figure 3E), and a mild increase in the mRNA levels of different SASPs (Figure 3F). However, we failed to observe a DNA-damage response upon ITGB3 expression (Figure S2A). Importantly, ITGB3 expression also induced senescence in IMR-90 fibroblasts, indicating that this response is not strain specific (Figures S2B–S2D). We confirmed that the activation of senescence by ITGB3 expression in BFs is dependent on the p53 pathway. Using a previously characterized shRNA targeting *TP53* (shp53) (Acosta et al., 2008), we impaired not only the proliferation arrest induced by *ITGB3* (Figure 3G, left panel) but also the increase in SA-β-Gal activity (Figure 3G, right panel). The use of a short interfering RNA (siRNA) targeting *TP53* (sip53) also impaired the growth arrest induced by ITGB3 expression (Figure S2E). Thus, ITGB3 ectopic expression induces senescence in human primary fibroblasts, which is dependent on the p53/p21^CIP^ pathway.

### *ITGB3* mRNA Levels Are Dynamically Regulated during Senescence

**Figure 4:**
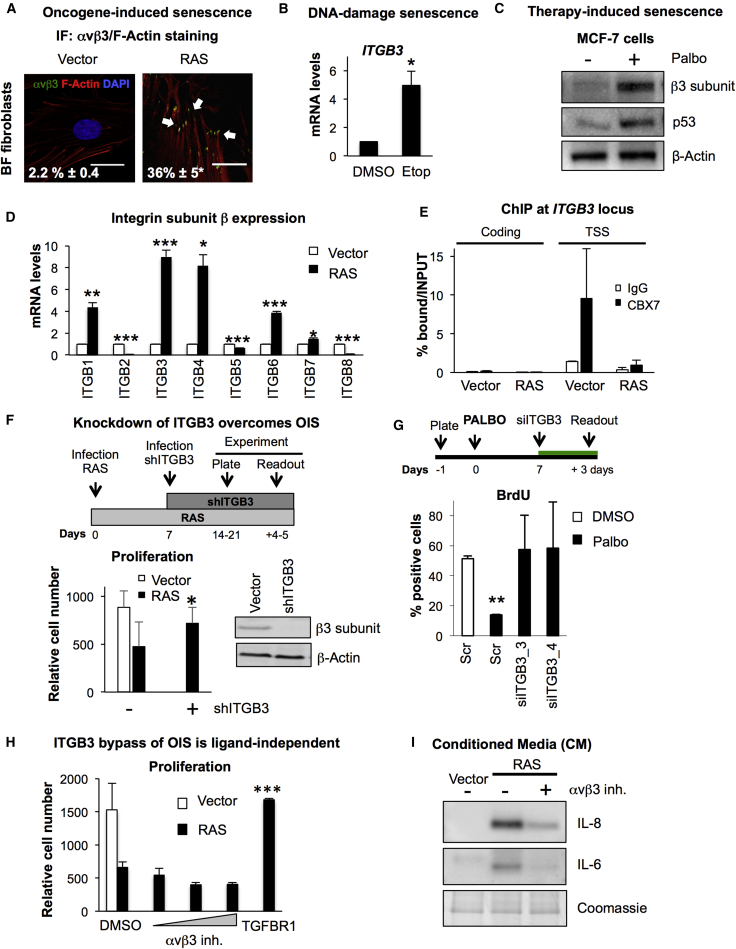
ITGB3 Regulates Senescence Independently of Its Binding Activity (A) Endogenous αvβ3 expression increases during OIS upon RAS expression in BFs. Representative pictures for αvβ3 (green) and F-actin (red) staining by IF in vector and RAS cells are shown. αvβ3-stained FA complexes are indicated with white arrows. Data represent the percentage of cells positive for αvβ3 staining. Scale bar, 20 μm. (B) *ITGB3* is endogenously upregulated during DNA-damage-induced senescence (DDIS). BFs were treated with 100 μM etoposide (Etop) for 2 days and replaced with fresh media for 5 days. (C) MCF7 breast cancer cells were treated with 200 nM palbociclib (Palbo) for 7 days, after which cells were lysed for immunobloting. An increase in β3 subunit and p53 can be observed after Palbo treatment. (D) mRNA analyses for *ITGB* subunits 1–8 during RAS-induced senescence in BFs. (E) CBX7 binding to *ITGB3* TSS is reduced during OIS. ChIP for CBX7 enrichment (black bars) versus IgG control (white bars) at an *ITGB3* TSS and a coding region in BFs expressing vector or RAS. (F) Schematic representation of the timings used to determine the role for ITGB3 overcoming OIS (top panel). Lower left panel: relative cell number in RAS-expressing BFs transduced with a vector or an shRNA targeting *ITGB3* (shITGB3). Lower right panel: representative immunoblot showing β3 subunit knockdown efficiency. (G) Top panel shows the experimental planning. Senescence was induced by Palbo treatment, after which siITGB3 was transfected (green bar). Two independent siRNAs targeting ITGB3 (siITGB3) overcame the senescence arrest induced by treating MCF7 cells with Palbo for 7 days. BrdU was added 24 hr before the end of the experiment. (H) Cells expressing RAS were treated with DMSO or αvβ3 inhibitor (cilengitide) for 48 hr, and the relative cell number was calculated. Increasing concentrations of cilengitide (10, 25, and 50 nM) show no reversion of the proliferation arrest induced by RAS. An inhibitor for TGF-β-receptor 1 (TGFBR1, 4 μM) was used as positive control. (I) Immunoblot for the conditioned media (CM) from cells expressing either vector or RAS treated with or without 50 nM of αvβ3 inhibitor (cilengitide) for 48 hr, followed by a 72-hr incubation in fresh media. Coomassie staining is shown as loading control. ^∗^p < 0.05; ^∗∗^p < 0.01; ^∗∗∗^p < 0.001.

We next decided to determine whether *ITGB3* was endogenously regulated during senescence. As OIS is a potent tumor suppressor mechanism both in vitro and in vivo (Muñoz-Espín and Serrano, 2014), we used BFs stably expressing the oncogene RAS. Staining for αvβ3 by IF shows a substantial increase in its expression levels upon RAS induction. In addition, αvβ3 co-localizes with F-actin, indicating that it is part of FA complexes during RAS activation (Figure 4A). We confirmed that β3 protein and transcript levels are endogenously upregulated upon RAS expression compared to the vector control in an additional strain of human and also in mouse fibroblasts (Figure S3A). We also observed the upregulation of β3 in BFs during DNA-damage-induced senescence (DDIS) induced by etoposide treatment (Figures 4B and S3B). Moreover, treatment of two different cancer cell lines, MCF7 (breast) and SK-HEP-1 (liver) (Bollard et al., 2016), with a CDK4/6 inhibitor (Palbociclib or Palbo), mimicking TIS, also triggered endogenous upregulation of β3 (Figures 4C, S3C, and S3D). Intriguingly, the upregulation of *ITGB3* mRNA levels in SK-HEP-1 cells could only be observed after 10 days of treatment with Palbo, concomitant with the establishment of senescence. To determine the temporal changes of *ITGB3* during senescence, we took advantage of IMR-90 fibroblasts expressing an endoplasmic reticulum (ER):RAS fusion protein (ER:RAS). Upon treatment with 4-hydroxytamoxifen (4OHT), senescence is progressively established, displaying an initial mitotic arrest followed by full senescence induction after 4–6 days treatment. Though the mRNA levels of *ITGB3* at early timepoints of the induction of senescence were downregulated, we did observe a consistent upregulation of *ITGB3* during the establishment of senescence (Figure S3E). This pattern highly resembles the recently described temporal changes induced by NOTCH1 during OIS (Hoare et al., 2016).

As integrins play a predominant role in cellular signaling and adhesion, we next investigated whether other integrin beta subunits were deregulated during OIS. To avoid the confounding effects of integrin changes during the initial phases of the establishment of senescence, we decided to measure the mRNA expression levels of *ITGB1-8* using BFs stably expressing RAS. Our data show a noticeable deregulation of integrin beta subunits during OIS, with upregulation of *ITGB1*, *3*, *4*, and *6* during RAS expression, *ITGB3* being the subunit most upregulated (Figure 4D). Therefore, we show that *ITGB3* mRNA levels increase concomitantly with the establishment of senescence.

### CBX7 Regulates *ITGB3* Locus during OIS

Since *ITGB3* locus is regulated by CBX7 (Figure 2), we reasoned that the endogenous upregulation of *ITGB3* mRNA upon RAS expression could be due to epigenetic regulation by CBX7. To test this hypothesis, we performed ChIP for endogenous CBX7 on the *ITGB3* locus in BFs transduced with vector or RAS. Our data show a reduced binding of CBX7 to the *ITGB3* TSS during OIS (Figure 4E), suggesting that the endogenous upregulation of *ITGB3* during OIS is due to the transcriptional deregulation of the locus by the loss of CBX7 binding. As expected, we did not observe changes in CBX7 binding in vector or RAS BFs in a control-coding region (Figure 4E).

### β3 Regulates Senescence Independently of Its Binding Activity

To test whether β3 has a functional role during senescence, we manipulated *ITGB3* mRNA levels during OIS and TIS, once senescence was fully established. The transduction of BFs expressing RAS with an shRNA targeting *ITGB3* (shITGB3) or transfection with two different siRNAs (siITGB3) impaired the proliferation arrest induced by RAS (Figures 4F, S3F, left panel, and S3G) and partially reverted the increase in the cell-cycle inhibitor p21^CIP^ induced upon OIS, as shown by IF (Figure S3F, right panel). Furthermore, ablation of *ITGB3* mRNA by siRNA also overcame the proliferation arrest induced by Palbo treatment in MCF7 cells (Figure 4G). We next treated BFs stably expressing RAS with the αvβ3/αvβ5 antagonist cilengitide, as we reasoned that inhibiting αvβ3 could be a therapeutic treatment to overcome senescence. Surprisingly, treatment of BFs expressing RAS with cilengitide could not reverse the proliferation arrest (Figure 4H) or the upregulation of p21^CIP^ or p16^INK4A^ protein levels (Figure S4A). This suggested that β3 induces senescence independently of its ligand-binding activity, which was further confirmed by the ectopic expression of a mutant β3, defective for the ligand-binding domain (ITGB3^D119A^) (Loftus et al., 1990) that also induced senescence (Figures S4B–S4E). Excitingly, although cilengitide treatment during OIS could not reverse the proliferation arrest induced by RAS or the upregulation of p16^INK4A^ o p21^CIP^ protein levels, we did observe a significant reduction of the SASP, as shown by measuring the levels of IL-8 and IL-6 secreted to the supernatant by immunoblot (Figure 4I). Therefore, αvβ3 inhibition is able to uncouple the SASP release from the proliferation arrest in OIS.

### β3 Regulates Cellular Senescence in a Cell-Autonomous Fashion by Activating the TGF-β Pathway

**Figure 5:**
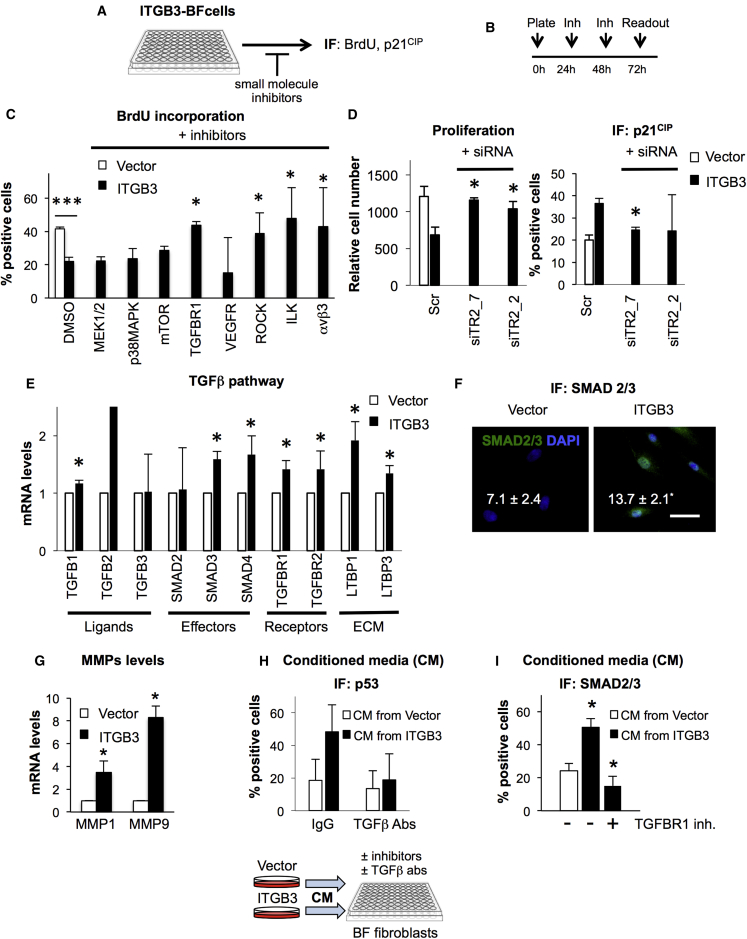
ITGB3 Induces Senescence by Activating the TGF-β Pathway in a Cell-Autonomous and Non-Cell-Autonomous Fashion (A and B) Shown here are (A) a schematic representation and (B) timings of a miniscreen for small molecule inhibitors (Inh) used to determine the pathway activating senescence induced by β3. (C) BFs expressing vector or ITGB3 were treated for 48 hr with a variety of drugs inhibiting different signaling pathways. Drugs were renewed every 24 hr, and BrdU was added 16 hr prior to fixing the cells. The percentage of BrdU-positive cells with or without the inhibitors is shown. The graph indicates the inhibitor’s targets: 40 μM PD98059 (targeting MEK1/2), 20 μM SB202190 (p38MAPK), 100 nM TORIN2 (mammalian target of rapamycin; mTOR), 4 μM TGF-β-R1 (TGFBR1), 8 μM Vegfr-2/Flt3/C-Kit (VEGFR), 150 nM GSK429286A (ROCK1/2, Rho-associated kinase), 50 nM Cpd22 (ILK, integrin-linked kinase), and 50 nM cilengitide (αvβ3). Except when indicated, asterisks represent the statistical differences for cells expressing ITGB3 treated with DMSO or the different small molecule inhibitors. (D) Knockdown of TGFBR2 overcomes senescence induced by β3. We measured the proliferation (left graph) and p21^CIP^ levels (right graph) in BF fibroblasts transiently transfected with a scramble (Scr) or two independent siRNAs against *TGFBR2* (siTR2_2 and 7) for 4–5 days. (E) qPCR analyses of different regulators of the TGF-β pathway: ligands (TGFB1, -2, and -3), effectors (SMAD2, -3, and -4), receptors (TGFBR1 or ALK5, TGFBR2), and ECM proteins (LTBP1, -3, or latent TGF binding proteins). (F) Representative IF pictures for SMAD2/3 staining and quantification of the percentage of cells positive for nuclear SMAD2/3. Scale bar, 50 μm. (G) *MMP1*–*MMP9* mRNA levels upon ITGB3 expression. (H and I) Normal BFs were treated with conditioned media (CM) from BFs expressing vector or ITGB3. (H) Pan-specific neutralizing anti-TGF-β1-3 antibodies inhibit the stabilization of p53 induced by the CM taken from ITGB3 cells. A species matching IgG was used as negative control. Bottom panel: a diagram showing the experimental planning. CM was collected after 7 days and transferred to normal BFs with or without different treatments. (I) A TGFBR1 inhibitor (4 μM) blocks the nuclear translocation of SMAD2/3 induced by the CM taken from ITGB3 cells. Data represent mean ± SD of 2–4 independent experiments. ^∗^p < 0.05; ^∗∗∗^p < 0.001.

To determine the pathway by which β3 induces senescence in BFs, we next used a panel of small molecule inhibitors. We assessed proliferation levels by quantifying BrdU incorporation and p21^CIP^ levels by IF (schematic representation of the drug screen and timings are shown in Figures 5A and 5B). Out of all the chemical compounds used, we found that the inhibitors targeting TGF-β-receptor 1 (TGFBR1 or ALK5), αvβ3/αvβ5 integrin (cilengitide), Rho-associated kinases 1/2 (ROCK1/2), and integrin-linked kinase (ILK) were capable of reversing not only the proliferation arrest induced by β3 expression (Figure 5C) but also the upregulation of p21^CIP^ (Figure S5A). Oddly, cilengitide did affect proliferation in this setting, which could be explained by compensation of αvβ5 upon ITGB3 overexpression (*ITGB5* is downregulated during OIS [Figure 4D] but not upon ITGB3 expression [data not shown]). As previous reports have shown that TGF-β regulates senescence (Acosta et al., 2013, Muñoz-Espín et al., 2013) and integrins are known to activate TGF-β (Asano et al., 2005, Margadant and Sonnenberg, 2010), we thought it would be interesting to determine whether β3 regulates senescence via activation of the TGF-β pathway. To further investigate this possibility, we analyzed the effect of a scramble siRNA (Scr) and two independent siRNAs targeting TGF-β-receptor 2 (siTR2_2 and siTR2_7) on the proliferation of BFs expressing vector or ITGB3. Our data demonstrate that both siRNAs against *TGFBR2* overcome senescence induced by the overexpression of ITGB3, as shown by measuring the relative cell number and p21^CIP^ levels by IF (Figures 5D and S5B). Interestingly, we could not reproduce these results using pan-specific neutralizing anti-TGF-β1–3 antibodies (data not shown), suggesting that ITGB3 regulates TGF-β, at least partially, in a cell-autonomous fashion.

The TGF-β superfamily comprises a number of molecular players, including receptors (TGFBR1 and -2), ligands (TGF-β1, -2, and -3), effectors (SMAD proteins), and ECM-binding proteins (LTBPs or latent TGF binding proteins). Upon binding of TGF-β ligands with TGFBRs, the pathway becomes active, and specific SMAD proteins translocate to the nucleus to control gene expression (Schmierer and Hill, 2007). We reasoned that, if β3 is inducing senescence by activating the TGF-β pathway, we should find differences in the expression levels of different members of this pathway. Indeed, qPCR analyses of a range of regulators implicated in the TGF-β pathway are upregulated in BFs expressing ITGB3 (Figure 5E). To further confirm that the pathway is active upon ITGB3 expression, we next measured the translocation of SMAD2/3 to the nucleus by IF. Indeed, BFs expressing ITGB3 showed a higher percentage of cells staining positive for nuclear SMAD2/3 (Figure 5F). Altogether, our data show that *ITGB3* regulates senescence via TGF-β activation.

### Non-Cell-Autonomous Effect of β3 on Human Primary Fibroblasts

Integrins can activate TGF-β embedded in the ECM by increasing the expression of matrix-degrading enzymes (matrix metalloproteinases; MMPs) and the proteolytic release of TGF-β to the media. Therefore, we determined the expression levels of different MMPs in BFs expressing ITGB3 and found upregulation of the mRNA levels of *MMP1* and *MMP9*, indicating that ITGB3 expression can directly activate the TGF-β pathway (Figure 5G). We next tried to determine whether TGF-β was being released to the supernatant in BFs expressing ITGB3 and had a non-cell-autonomous role on surrounding cells. While we could not detect TGF-β in the supernatant (data not shown), we did find that the conditioned media (CM) from cells expressing ITGB3 had an effect on normal BFs by inducing the stabilization of p53 protein (Figure 5H), the nuclear translocation of SMAD2/3 (Figure 5I), and a reduced proliferation rate (Figure S5C) in normal BFs. Furthermore, treatment with a pan-specific neutralizing anti-TGF-β1-3 antibody (Figure 5H) or an inhibitor for TGFBR1 (Figure 5I) abrogated the effect of the CM from ITGB3 cells, suggesting that the non-cell-autonomous effect of ITGB3 cells is dependent on TGF-β.

### β3 Subunit Expression Increases during Replicative Senescence

**Figure 6:**
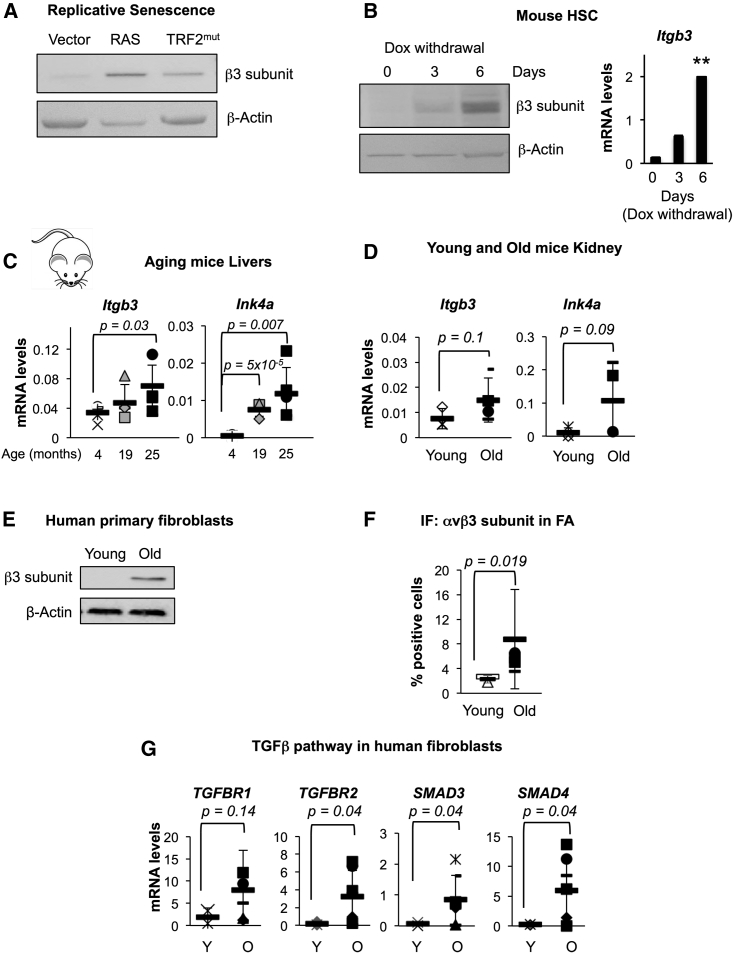
β3 Subunit Is Upregulated during Replicative Senescence and Aging in Human and Mouse (A) Immunoblot for β3 subunit in BFs expressing empty vector, RAS or the dominant-negative telomeric repeat binding factor 2 (TRF2^ΔBΔM^), mimicking replicative senescence. β-actin is used as loading control. (B) Immunoblot for β3 subunit (left panel) and qPCR analysis for *Itgb3* mRNA levels (right panel) in mouse hepatic stellate cells (mHSCs) upon different days of doxycycline (Dox) withdrawal. (C) mRNA levels are shown for *Ink4a* and *Itgb3* in livers taken from C57BL/6J female mice aged 4, 19, and 25 months old. (D) Kidneys from young (4 months) and old (25 months) C57BL/6J mice were subjected to qPCR to determine *Ink4a* and *Itgb3* mRNA expression levels. (E) Representative immunoblot for β3 subunit in human primary fibroblasts derived from young and old donors. We observed similar results with other young and old samples. β-actin is the loading control. (F) Quantification of the percentage of cells stained positive for αvβ3 in FA complexes by IF in young and old human fibroblasts. Data represent the mean ± SD of fibroblasts derived from young and old donors. (G) qPCR analyses for TGF-β receptors 1 and 2 and SMAD3 and 4 in human fibroblasts from young (Y) and old (O) donors. In (C) and (D), data represent the mean ± SD of 4–5 mice per condition. In (F) and (G), data represent the mean ± SD from fibroblasts from 2–4 young and 6–7 old donors.

Activation of senescence has been described in a variety of physiological and pathological conditions, including aging. In fact, the activation of cellular senescence is considered one of the hallmarks of aging (López-Otín et al., 2013). In order to determine whether the β3 subunit is upregulated during aging, we used a retroviral construct encoding the dominant-negative allele of the telomeric repeat binding factor 2 (TRF2^ΔBΔM^). Expression of TRF2^ΔBΔM^ in primary fibroblasts rapidly mimics the process of replicative senescence and aging (Karlseder et al., 1999). Similar to our previous results, where β3 is upregulated in senescence, TRF2^ΔBΔM^-expressing BFs presented an increase in β3 subunit (Figure 6A). This was further confirmed in murine hepatic stellate cells (mHSCs) extracted from an adult mouse harboring a doxycycline (Dox)-inducible construct to express shp53 (Lujambio et al., 2013). Upon Dox withdrawal, senescence is induced by re-expression of p53 and mHSCs showed an increase in a number of markers of senescence (Figure S6A), concomitant with the accumulation of *Itgb3* (Figure 6B).

### *ITGB3* mRNA Levels Are Dynamically Upregulated during Aging in Mice

To determine whether *Itgb3* expression is changed during aging in vivo, we extracted RNA from liver tissue of C57BL/6J female mice aged 4, 19, and 25 months. Livers from mice aged 19 months presented a dynamic increase at the mRNA levels of *Cdkn2a* and *Itgb3*, but the highest increase was observed in 25-month-old mice, where additional markers of senescence were observed (Figures 6C and S6B). This is in agreement with our in vitro data that show a concomitant upregulation of *ITGB3* with *CDKN2A* mRNA levels (Figure S3E). Upregulation of *Itgb3* mRNA and other markers of senescence were also observed in kidney (Figures 6D and S6B) and, to a lesser extent, in the intestine in 25-month-old mice (Figures S6B and S6C).

### β3 Subunit and TGF-β Components Are Highly Expressed in Fibroblasts Derived from Old Human Donors

Next, we took advantage of primary skin fibroblasts derived from young (∼10 years old) and old (∼80 years old) human donors and tested whether a correlation between senescence and β3 subunit existed during aging (Figure S6D). In order to confirm that fibroblasts derived from old donors behaved as senescent cells, we analyzed different senescence markers. We could, indeed, observe that fibroblasts derived from old donors presented a reduced proliferative capacity measured by relative cell number (Figure S6E) and had a significant increase in p16^INK4A^ and p21^CIP^ protein levels, as measured by IF, compared to young donor cells (Figure S6F). We then analyzed the expression levels of β3 subunit between fibroblasts from young and old donors and observed an increase in β3 by immunoblotting (Figure 6E), an increase in the percentage of αvβ3 staining in FA complexes by IF (Figures 6F and S6G), and an increase in *ITGB3* at the RNA level (Figure S6H) in fibroblasts from old compared to young donors. We have previously shown that activation of senescence by β3 is dependent on the TGF-β pathway. To determine whether the same activation pathway applies to fibroblasts derived from human donors, we analyzed the expression levels of different regulators of the TGF-β pathway. Interestingly, we could also observe an increase in the mRNA levels of different regulators of the TGF-β pathway, including TGF-β receptors 1 and 2 and SMAD3 and 4 (Figure 6G). Altogether, these data show the existence of a positive correlation between senescence and the expression levels of β3 subunit and different regulators of the TGF-β pathway in aging.

### *ITGB3* Plays a Role in Aging in Fibroblasts Derived from Old Human Donors

**Figure 7:**
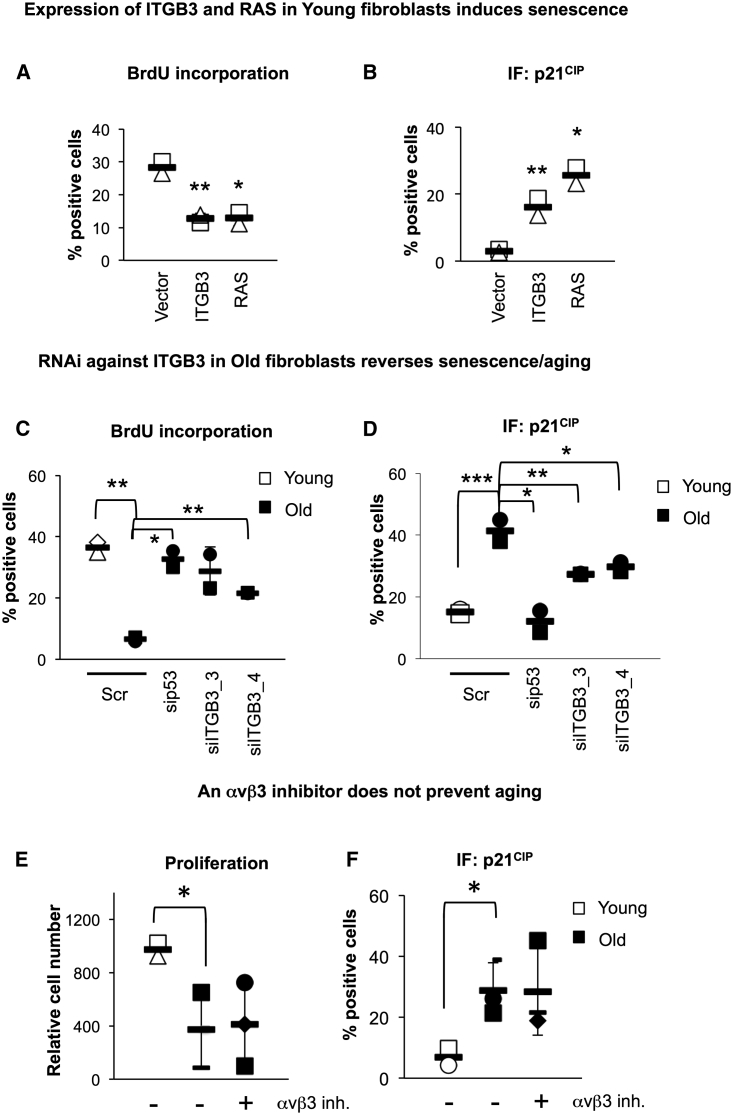
Changes in the Expression Levels of *ITGB3* Affect Aging and Senescence Cellular Features (A and B) Ectopic expression of a construct encoding either RAS or ITGB3 induces a senescence-like arrest in human fibroblasts derived from two independent young donors. (A) Percentage of BrdU-positive cells and (B) p21^CIP^ protein levels quantified by IF are shown. (C and D) RNAi targeting *ITGB3* in fibroblasts derived from old donors averts cellular features of aging and senescence. Cells derived from two young donors were used as controls (white filling). Fibroblasts from old donors (black filling) were transiently transfected with a scramble (Scr) or two independent siRNAs against *ITGB3* (siITGB3_3 and 4) for 4–5 days. sip53 was used as a control. (C) The percentage of cells staining positive for BrdU and (D) p21^CIP^ were quantified by IF. (E and F) Treatment of cells derived from old donors with cilengitide does not affect senescence/aging. Cells from two independent old donors (black filling) were treated with 50 nM cilengitide αvβ3 inhibitor for 2 days, and (E) the percentage of BrdU- and (F) p21^CIP^-positive cells was assessed. inh., inhibitor. All data represent the mean ± SD from cells derived from two independent young (A and B) or old (C–F) individuals. ^∗^p < 0.05; ^∗∗^p < 0.01; ^∗∗∗^p < 0.001.

We next decided to manipulate the expression levels of ITGB3 in fibroblasts derived from young and old donors to identify whether *ITGB3* plays a role in this model. As expected, ectopic expression of either ITGB3 or RAS in fibroblasts derived from two different young donors induced senescence-like growth arrest, as observed by a reduction in the percentage of cells incorporating BrdU (Figure 7A) and an upregulation of cells staining positive for p21^CIP^ (Figure 7B). We next decided to determine whether reducing the endogenous levels of *ITGB3* mRNA in cells derived from old donors could attenuate aging. To this end, we chose the two fibroblasts from old donors that expressed the highest levels of β3, and we reduced *ITGB3* expression levels using RNAi. Transfection with two different siRNAs (siITGB3) and sip53, overcame the proliferation arrest characteristic of old fibroblasts (Figure 7C) and partially reverted the increase in the cell-cycle inhibitor p21^CIP^ (Figure 7D). This was further confirmed using shITGB3 (Figure S7A). As our previous data show that ITGB3 induces senescence independently of its ligand-binding activity, we investigated whether this mechanism was conserved during aging. Treatment of old donor cells with cilengitide could not attenuate senescence, neither the proliferation arrest nor p21^CIP^ upregulation (Figures 7E and 7F), suggesting that the role for ITGB3 in aging in human primary fibroblasts is independent of its ligand-binding activity. Altogether, these data suggest that ITGB3 is a regulator of aging in the human primary fibroblasts derived from old donors in this study.

## Discussion

Intercellular communication is an important feature to maintain tissue homeostasis, where the activation of cellular senescence plays a crucial role. In fact, previous reports have found ECM remodeling to regulate fibrosis by activating the senescence program (Jun and Lau, 2010, Krizhanovsky et al., 2008, Lujambio et al., 2013). Apart from inflammation and ECM remodeling, cells can communicate via the secretion of extracellular vesicles (Tkach and Théry, 2016), cell-cell contact (Hoare et al., 2016), or intercellular protein transfer (Biran et al., 2015). Here, we provide evidence that the integrin β3 subunit plays a role in senescence through activation of the TGF-β pathway.

A great deal of information exists regarding the biological function of integrins and their regulation of the microenvironment, but relatively little is known about the transcriptional regulation of integrins themselves. A recent report has found that MYC overexpression leads to a direct downregulation of *ITGB3*, inducing decreased motility and invasiveness (Liu et al., 2012). Our results further add PRC1 complex as a regulator of the *ITGB3* locus in normal fibroblasts and for CBX7 during OIS, where CBX7 binding to the *ITGB3* TSS is reduced. In fact, *ITGB3* has been previously identified as a Polycomb target by ChIP sequencing in other biological contexts, such as mouse embryonic stem cells (ESCs) (Morey et al., 2013) and human primary fibroblasts (Pemberton et al., 2014), suggesting that the *ITGB3* locus is epigenetically regulated in several biological contexts.

Integrin signaling regulates diverse functions in cancer, angiogenesis, stemness, and drug resistance (Desgrosellier and Cheresh, 2010). In addition, integrins also regulate fibrosis and wound healing (Margadant and Sonnenberg, 2010). Our findings establish the β3 subunit as a regulator for cellular senescence. We show that β3 subunit expression accelerates the onset of senescence in human primary fibroblasts, which is dependent on the activation of the p21^CIP^/p53 pathways. This is complementary with a previous study, which shows activation of fibroblast senescence by the ECM protein CCN1 that binds to α6β1, activating ROS production (Jun and Lau, 2010). Our results also show a robust expression of β3 upon senescence activation induced by a variety of stimuli, while interference with its expression levels disrupts the senescence phenotype. In contrast, it was found that αvβ3 expression escapes OIS in glioblastoma by activating PAK4 (Franovic et al., 2015) and that mice expressing β1-deficient tumors show reduced tumor burden and activation of senescence (Kren et al., 2007). Furthermore, mice lacking β3 accelerate wound-healing closure, which could be by restricting the induction of senescence. However, in contrast with our findings, the authors observed an increase in TGF-β signaling in β3 null mice (Reynolds et al., 2005). All these seemingly paradoxical behaviors of integrin-signaling activity could be due to differences in the cellular and environmental contexts during senescence activation, as it has been previously described for H-Ras^G12V^ (Serrano et al., 1997) and the chemokine receptor CXCR2 (Acosta et al., 2008).

Cellular adhesion is a key feature of senescence. In agreement with our results, several reports have found differential expression of integrins during cellular senescence activation. Analysis of published datasets show that the “cellular adhesion” pathway and integrins are differentially expressed during senescence activation (Fridman and Tainsky, 2008, Storer et al., 2013). Likewise, a number of studies have found that TGF-β ligands are part of the SASP and play an important role in senescence through p21^CIP^ regulation, in agreement with our data (Acosta et al., 2013, Hoare et al., 2016, Muñoz-Espín et al., 2013, Storer et al., 2013). The TGF-β superfamily controls numerous cellular and biological processes, such as development, regeneration, fibrosis, and cancer (Macias et al., 2015). Accumulating evidence indicates that a cross-talk between integrins and TGF-β exists, in particular to regulate fibrosis, wound healing, and cancer (Asano et al., 2005, Margadant and Sonnenberg, 2010). However, even if senescence is known to regulate all these biological processes, none of these studies have reported the existence of a cross-talk between integrins and TGF-β in senescence or aging. Our data show that β3 regulates senescence by activating TGF-β via cell-autonomous and non-cell-autonomous mechanisms. The use of small molecule inhibitors, RNAi technology, and the analysis of the expression levels of various members of the TGFβ pathway authenticate a role for TGF-β during senescence induced by β3 expression.

Different reports have found that there is cross-talk between integrins and chemokine receptors (Desgrosellier and Cheresh, 2010). Although we could not detect any changes in the mRNA expression levels of *CXCR2* in cells expressing ITGB3 (data not shown), it would be interesting to further investigate a potential connection in senescence.

Senescence regulates tissue-regenerative capacity and homeostasis. In fact, αvβ3 expression is increased in a number of cell types undergoing tissue remodeling (Asselin-Labat et al., 2007, Brooks et al., 1994). Furthermore, integrins can direct specific stemness-related reprogramming, providing an important role during development independent of their ligand-binding activity (Seguin et al., 2015). Interestingly, developmental senescence is activated to promote tissue remodeling and stem cell renewal (Muñoz-Espín and Serrano, 2014). Our data show that senescence induced by β3 presents a similar pattern to developmental senescence (activation of p21^CIP^, TGF-β/SMAD, and no DNA damage) and that it is independent of ligand binding. It would be interesting to investigate whether integrins also play a role in this context.

Our data show an increase in the expression levels of *Itgb3* mRNA concomitant with an increase in different markers of senescence in tissue from old mice. Upregulation of β3 and senescence/aging markers, including TGF-β members, was further observed in fibroblasts from old human donors. This is in accordance with previous reports, which have found that p16^INK4A^ levels correlate with chronological age in most tissues analyzed, both in mice (Baker et al., 2016, Krishnamurthy et al., 2004) and in humans (Ressler et al., 2006). Interestingly, knockdown of *ITGB3* mRNA partially reversed the aging phenotype of fibroblasts derived from old human donors. However, the αvβ3 antagonist, cilengitide, could not reverse aging, suggesting that the role for β3 in this cellular system is independent of its ligand-binding activity. Our data show that cilengitide has a diverse effect on the SASP and on the senescence growth arrest. As senescent cells accumulate during aging, causing chronic inflammation (van Deursen, 2014), cilengitide could be a potential therapeutic route to block inflammation without affecting proliferation in aging.

In summary, here, we provide evidence for the β3 subunit being a marker and regulator of senescence. Our results demonstrate the importance of FA complex formation regulating the microenvironment during senescence activation and identify integrins as potential therapeutic targets to promote healthy aging.

## Experimental Procedures

The care and use of mice were in accordance with the UK Home Office regulations and the UK Animals (Scientific Procedures) Act of 1986.

### Cell Culture and Retroviral and Lentiviral Infections

MCF7, SK-HEP-1, and IMR-90 were obtained from the American Type Culture Collection. BFs were described previously (Pemberton et al., 2014). Donor primary human fibroblasts were obtained from the Coriell Cell Repository. Cells were maintained in DMEM (Invitrogen) with 10% fetal bovine serum (FBS) (PAA Laboratories) and 1% antibiotic-antimycotic solution (Invitrogen). Mouse hepatic stellate cells were maintained in the same media supplemented with 1 μg/mL Dox. Methods used for retrovirus and lentivirus production and infection have been previously described (O’Loghlen et al., 2012).

### Treatment with Kinase Inhibitors

BFs were seeded at the same density in 96-well or 24-well plates. Inhibitors for different signaling pathways were added at the concentrations detailed in Table S2. BFs were incubated with the inhibitors for 48 hr, renewing after 24 hr. Cells were fixed 24 hr later.

### Conditioned Media Experiments

The indicated cells were cultured for 7 days in DMEM in 0.5% FBS. The conditioned media (CM) were collected and supplemented to generate 10% FBS CM, and normal cells were treated with or without pan-specific TGF-β1-3 antibodies or the TGFBR1 inhibitor (4 μM) for 72 hr. For the immunoblotting, CM was concentrated using Amicon Ultra Centrifugal Filters (Millipore) and stained with IL-8/IL-6 antibodies.

### Statistics

Results are expressed as the mean ± SD, and statistical analysis was performed using a Student’s t test. A p < 0.05 was considered significant. ^∗^p < 0.05; ^∗∗^p < 0.01; ^∗∗∗^p < 0.001.

## Author Contributions

V.R. performed most of the experiments, except where specified. A.O., V.E., and A.P.S. performed and analyzed the SILAC data. CDK4/6 inhibitor experiments were performed by M.B., V.M., and A.L. A.O. conceived and designed the study and analyzed most data. A.O. wrote and edited the manuscript, with input from all the authors.

## Accession Numbers

The accession number for the proteomics dataset reported in this paper is PRIDE: PXD005717.

## Supplemental Information

Document S1. Supplemental Experimental Procedures, Figures S1–S7, and Tables S1 and S2

Document S2. Article plus Supplemental Information
