# Supplementary material for: Integrin Beta 3 Regulates Cellular Senescence by Activating the TGF-β Pathway
Source: Cell Rep. 2017 Mar 7;18(10):2480–93. doi: 10.1016/j.celrep.2017.02.012 (PMC5357738; doi:10.1016/j.celrep.2017.02.012)
Supplement: Document S1. Supplemental Experimental Procedures, Figures S1–S7, and Tables S1 and S2 [file mmc1.pdf]

Cell Reports, Volume *18*

Supplemental Information

Integrin Beta 3 Regulates Cellular Senescence by Activating the TGF-β Pathway

Valentina Rapisarda, Michela Borghesan, Veronica Miguela, Vesela Encheva, Ambrosius P. Snijders, Amaia Lujambio, and Ana O'Loghlen

Rapisarda *et al.* **Figure S1**

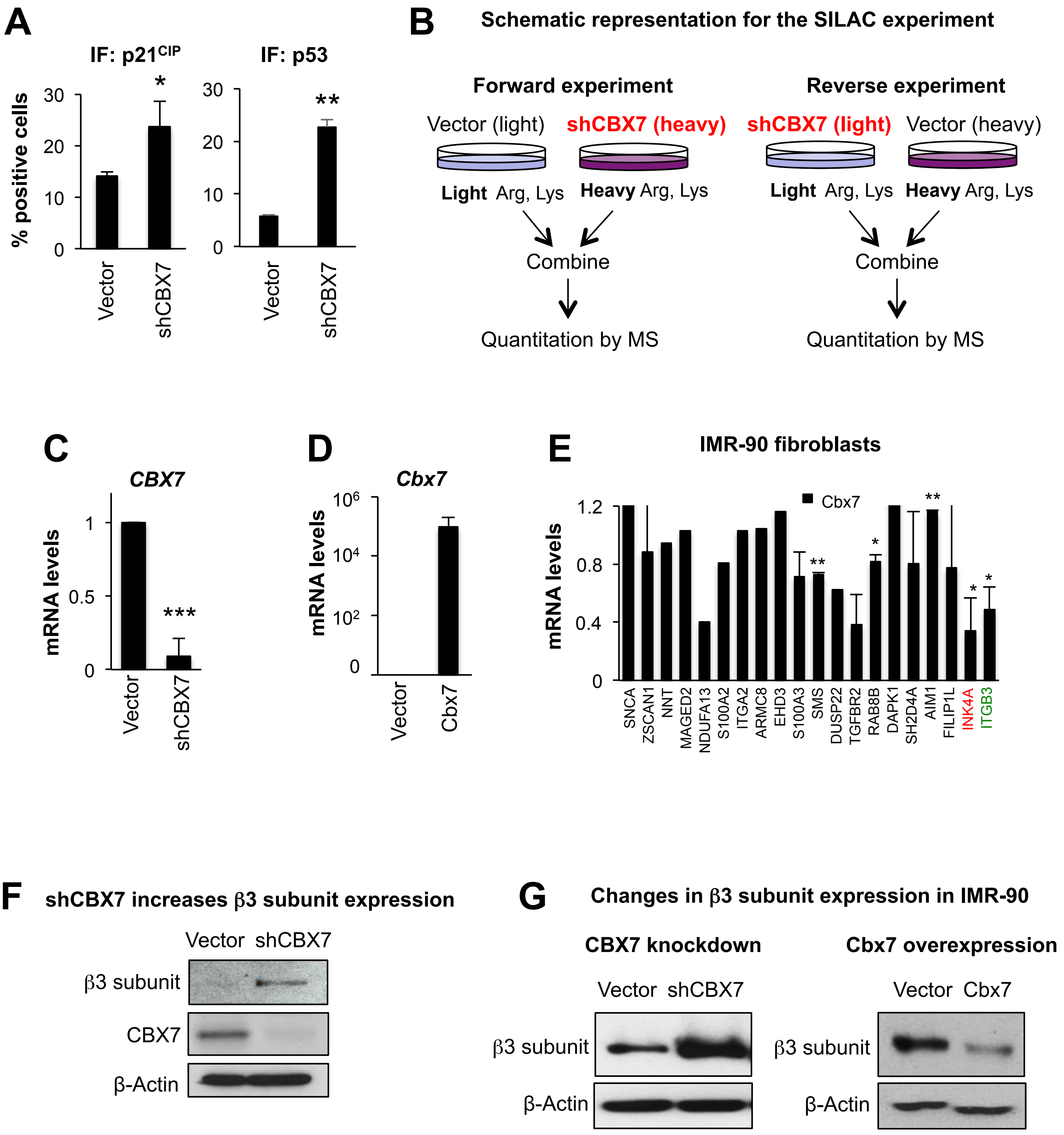

Figure S1. CBX7 knockdown induces senescence in human primary breast fibroblasts (Related to Figures 1 and 2)
(A) Knockdown of *CBX7* (shCBX7) in BF fibroblasts induces an increase in the percentage of cells staining positive for p53 and $p21^{CIP}$. (B) Scheme for the strategy followed to perform the SILAC experiment. In the forward experiment, we grow BF infected with shCBX7 in the media containing "heavy aminoacids", whereas in the reverse experiment we culture shCBX7 cells in the media with "light aminoacids". (C) qPCR showing *CBX7* knockdown efficiency at the mRNA level in BF transduced with shCBX7. (D) Ectopic expression of mouse Cbx7 by retroviral transduction shows an increase in Cbx7 mRNA by qPCR. (E) qPCR analyses show that Cbx7 expression downregulates the genes of the SILAC proteins in IMR-90 fibroblasts. Data is normalized to the control, and represent the mean ± SD of 1-3 independent experiments. (F) Representative immunoblot showing an increase in β3 subunit levels, concomitant with a decrease in CBX7 protein levels in BF transduced with shCBX7. β-Actin is used as loading control. (G) Representative immunoblot showing changes in β3 subunit levels upon CBX7 knockdown or overexpression in IMR-90 fibroblasts. β-Actin is used as loading control.

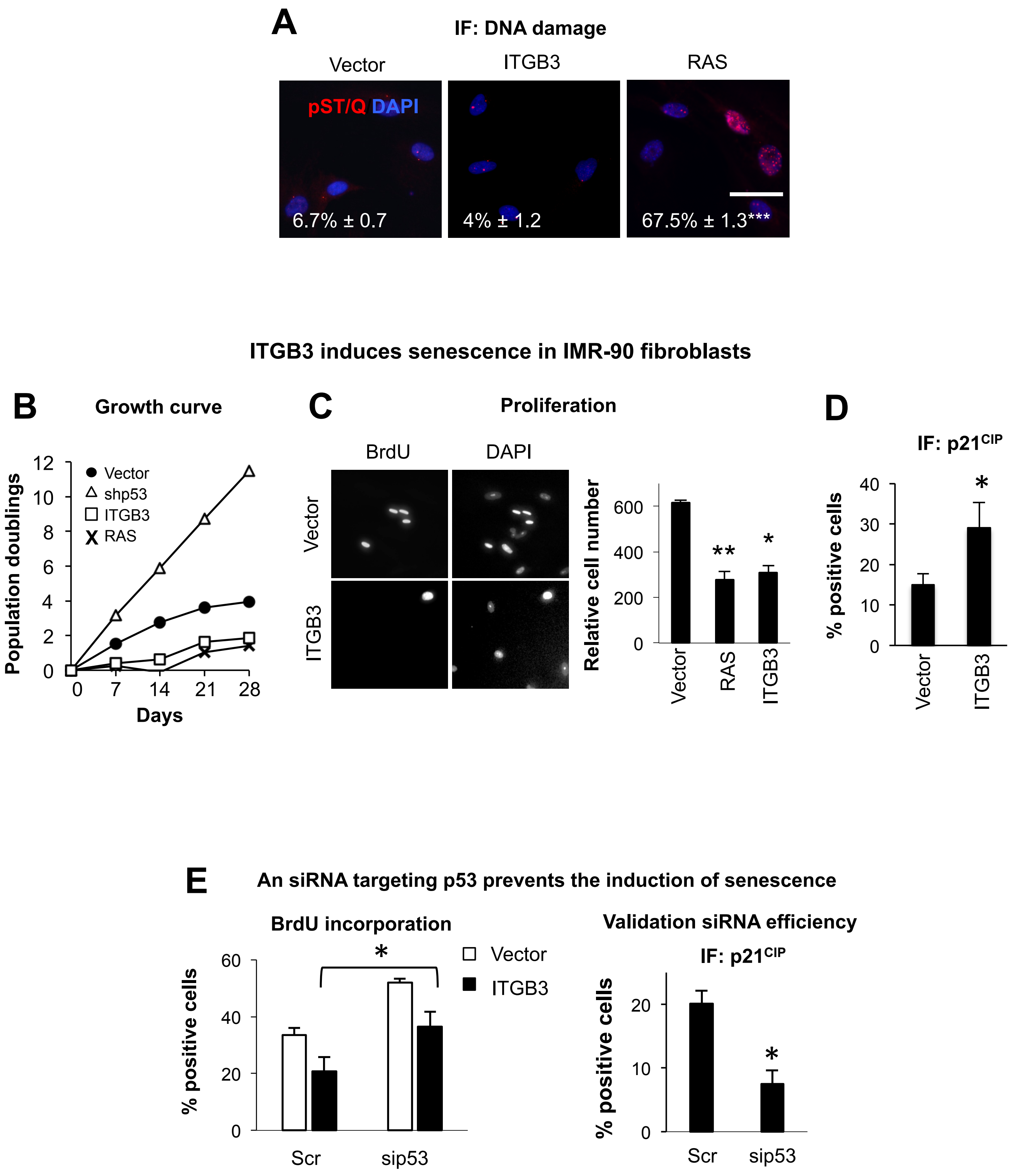
A
IF: DNA damage
Vector
ITGB3
RAS
pST/Q DAPI
6.7% ± 0.7
4% ± 1.2
67.5% ± 1.3***
ITGB3 induces senescence in IMR-90 fibroblasts
B
Growth curve
Population doublings
Vector
shp53
ITGB3
RAS
0
2
4
6
8
10
12
0
7
14
21
28
Days
C
Proliferation
BrdU
DAPI
Vector
ITGB3
Relative cell number
0
200
400
600
**
*
Vector
RAS
ITGB3
D
IF: p21CIP
% positive cells
0
10
20
30
40
*
Vector
ITGB3
E
An siRNA targeting p53 prevents the induction of senescence
BrdU incorporation
% positive cells
0
20
40
60
*
Vector
ITGB3
Scr
sip53
Validation siRNA efficiency
IF: p21CIP
% positive cells
0
10
20
*
Scr
sip53

Figure S2. ITGB3 induces senescence dependent on the p53 pathway (Related to Figure 3)
(A) BF expressing ITGB3 do not display DNA damage, measured by pST/Q staining, and calculated by quantifying the percentage of cells positive for pST/Q staining. RAS was used as a positive control for DNA-damage in senescence. (B) IMR-90 primary fibroblasts transduced with a vector encoding ITGB3 show reduced proliferation, measured by calculating the population doubling growth curve. H-Ras^{G12V} (RAS) and shp53 expressing fibroblasts were used as positive and negative regulators of senescence, respectively. (C) A reduction in the proliferation rate is also shown in representative pictures for BrdU incorporation (left panel) and by measuring the relative cell numbers (right panel). (D) IMR-90 fibroblasts expressing ITGB3 also show an increase in the percentage of cells staining positive for p21^{CIP} protein by IF. (E) Disruption of *TP53* mRNA prevents the activation of senescence induced by the overexpression of ITGB3. BF expressing either Vector or ITGB3 were reverse-transfected with a Scramble (Scr) RNAi or an siRNA targeting p53 (sip53). Left panel: proliferation was quantified by measuring the percentage of cells incorporating BrdU. Right panel: Knockdown efficiency for sip53 was quantified by measuring the levels of p21^{CIP}, a target of p53. Data represents the mean ± SD of 2-3 independent experiments. Scale bar, 50μm

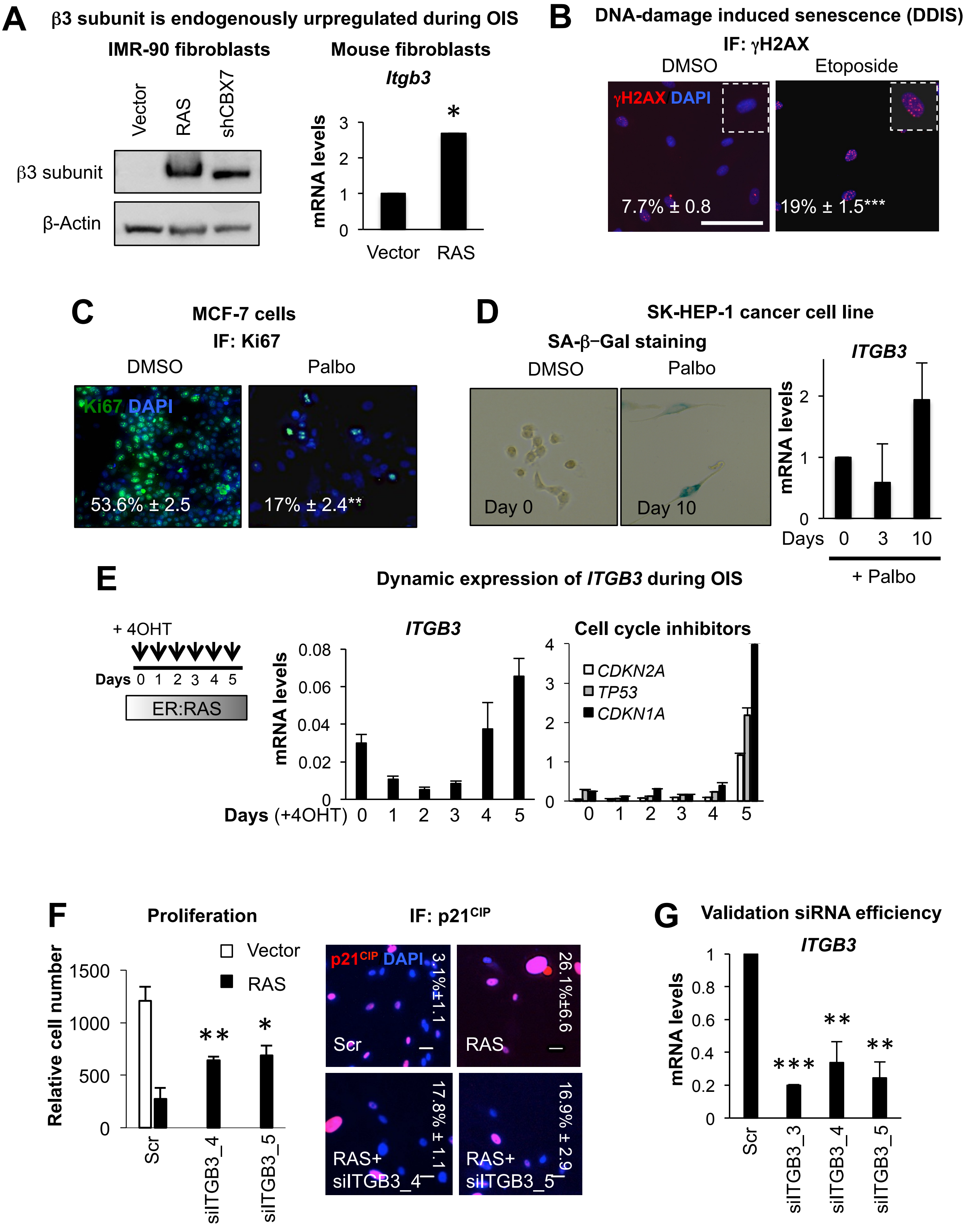
A
β3 subunit is endogenously urpregulated during OIS
IMR-90 fibroblasts
Vector
RAS
shCBX7
β3 subunit
β-Actin
Mouse fibroblasts
Itgb3
mRNA levels
*
Vector
RAS
B
DNA-damage induced senescence (DDIS)
IF: γH2AX
DMSO
Etoposide
γH2AX DAPI
7.7% ± 0.8
19% ± 1.5***
C
MCF-7 cells
IF: Ki67
DMSO
Palbo
Ki67 DAPI
53.6% ± 2.5
17% ± 2.4**
D
SK-HEP-1 cancer cell line
SA-β-Gal staining
DMSO
Palbo
Day 0
Day 10
ITGB3
mRNA levels
Days 0 3 10
+ Palbo
E
Dynamic expression of ITGB3 during OIS
+ 4OHT
Days 0 1 2 3 4 5
ER:RAS
ITGB3
mRNA levels
Days (+4OHT) 0 1 2 3 4 5
Cell cycle inhibitors
CDKN2A
TP53
CDKN1A
F
Proliferation
Vector
RAS
Relative cell number
**
*
Scr
siITGB3_4
siITGB3_5
IF: p21CIP
p21CIP DAPI
3.1%±1.1
Scr
26.1%±6.6
RAS
17.8% ± 1.1
RAS+ siITGB3_4
16.9% ± 2.9
RAS+ siITGB3_5
G
Validation siRNA efficiency
ITGB3
mRNA levels
***
**
**
Scr
siITGB3_3
siITGB3_4
siITGB3_5

Figure S3. *ITGB3* is a regulator of cellular senescence (Related to Figure 4)
(A) Left panel shows immunoblot for β3 subunit in IMR-90 fibroblasts expressing RAS and shCBX7. β−Actin is used as loading control; right panel shows relative *Itgb3* mRNA levels upon RAS expression in mouse embryonic fibroblasts (MEFs) measured by qPCR. A representative qPCR is shown. (B) BF treated with etoposide show an increase in DNA-damage. This was measured by staining with a phospho-γ-H2AX antibody by IF. The quantification represents the percentage of cells with positive staining. Scale bar, 50μm. (C) MCF7 breast cancer cells were treated with 200nM Palbociclib for 7 days. Proliferation was measured by staining with a Ki67 antibody. Data shows the percentage of cells staining positive for Ki67. (D) SK-HEP-1 hepatocellular carcinoma cells were treated with 1μM Palbo for 3 or 10 days and stained for SA-β-Gal (left) or subjected to qPCR analyses for *ITGB3* mRNA levels (right panel). (E) *ITGB3* is dynamically regulated during cellular senescence. Time-course treatment with 200nM 4-Hydroxytamoxifen (4OHT) of fibroblasts harboring an ER:RAS construct. RNA was collected at different time points and subjected to qPCR analysis to determine *ITGB3*, *CDKN2A*, *TP53* and *CDKN1A* levels. (F) BF expressing vector or RAS were transfected with a scramble siRNA (Scr) or two different siRNA against ITGB3 (siITGB3_4 and 5). 4 days after transfection, BrdU was added and 24h later BrdU incorporation and $p21^{CIP}$ levels were assessed. Left panel: relative cell number. Right panel: representative images for $p21^{CIP}$ staining by IF. Scale bar, 100μm. (G) qPCR showing mRNA levels for *ITGB3* in BF fibroblasts transfected with three independent siRNA targeting *ITGB3* (siITGB3).

Rapisarda *et al.* **Figure S4**

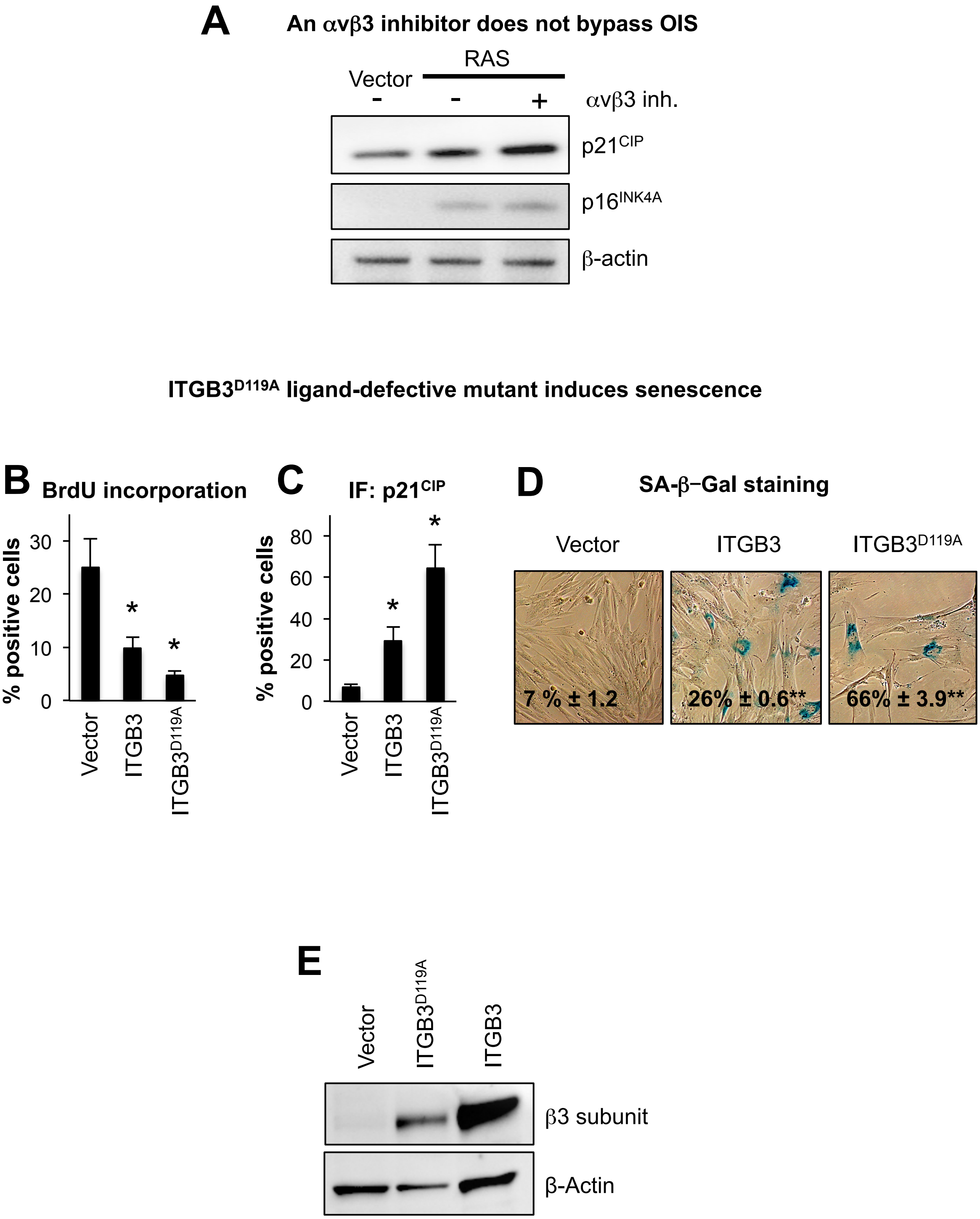

Figure S4. ITGB3 induces senescence independently of its ligand-binding activity (Related to Figure 4)

(A) Immunoblot for $p21^{CIP}$ and $p16^{INK4A}$ protein levels in BF cells expressing either Vector or RAS. RAS cells were treated with DMSO or 50nM of an $\alpha v\beta 3$ inhibitor (cilengitide) for 48h, washed and followed by 72h of fresh media incubation. β-actin is shown as loading control. (B-D) Expression of an ITGB3 mutant construct ($ITGB3^{D119A}$), defective for ligand-binding activity, induces senescence in BF fibroblasts. ITGB3 wild type is used as control. (B) BrdU proliferation is reduced in cells expressing $ITGB3^{D119A}$, while (C) $p21^{CIP}$ protein levels and the (D) the percentage of cells staining positive for SA-β-Gal are increased. (E) Immunoblot for β3 subunit shows the expression levels for ITGB3 wild-type and mutant ($ITGB3^{D119A}$) construct.

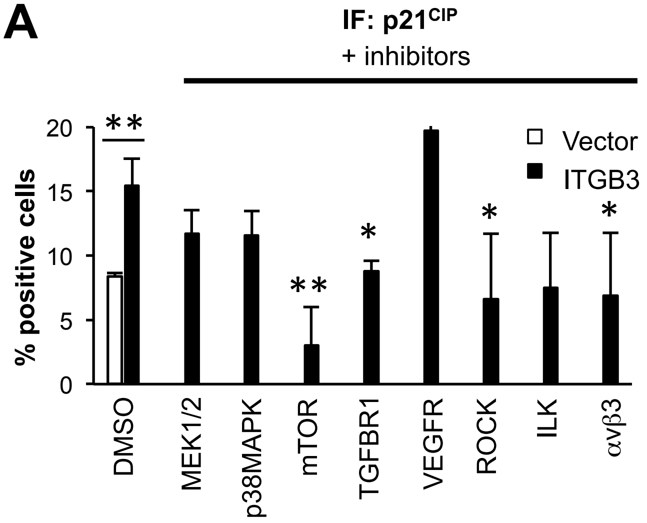
A
IF: p21CIP
+ inhibitors
% positive cells
0
5
10
15
20
**
**
*
*
*
Vector
ITGB3
DMSO
MEK1/2
p38MAPK
mTOR
TGFBR1
VEGFR
ROCK
ILK
αvβ3

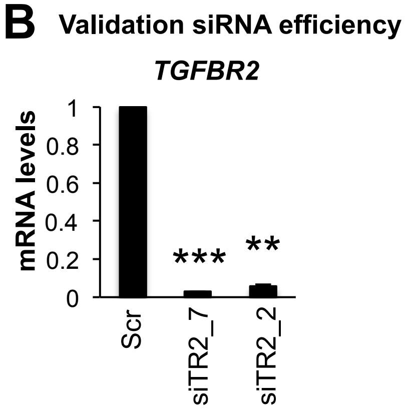
B
Validation siRNA efficiency
TGFBR2
mRNA levels
0
0.2
0.4
0.6
0.8
1
***
**
Scr
siTR2_7
siTR2_2

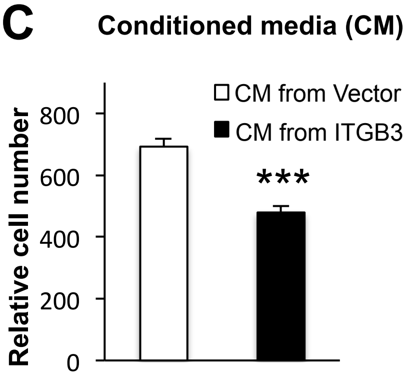
C
Conditioned media (CM)
Relative cell number
0
200
400
600
800
CM from Vector
CM from ITGB3
***

Figure S5. ITGB3 induces senescence in a cell and non-cell autonomous fashion by activating the TGFβ pathway (Related to Figure 5)

(A) BF expressing vector or ITGB3 were treated for 48h with a small molecule drug screen. The percentage of $p21^{CIP}$ positive cells with or without the inhibitors is shown. The graph indicates the inhibitor's targets. Inhibitor's details are: 40μM PD98059 (targeting MEK1/2), 20μM SB202190 (p38MAPK), 100nM TORIN2 (mTOR), 4μM TGFβ-R1 (TGFBR1), 8μM Vegfr-2/Flt3/C-Kit (VEGFR), 150nM GSK429286A (ROCK1/2, Rho-associated kinase), 50nM Cpd22 (ILK, integrin-linked kinase) and 50nM Cilengitide (αvβ3). (B) qPCR analyses to show the mRNA knockdown efficiency of the RNAi targeting *TGFBR2* (siTR2_7 and siTR_2). (C) Conditioned media (CM) from BF expressing either Vector or ITGB3 was used to treat normal BF cells. Conditioned media was produced in 0.5% FBS for 7 days. The relative cell number was calculated after treating the cells for 72h with the conditioned media.

Rapisarda *et al.* **Figure S6**

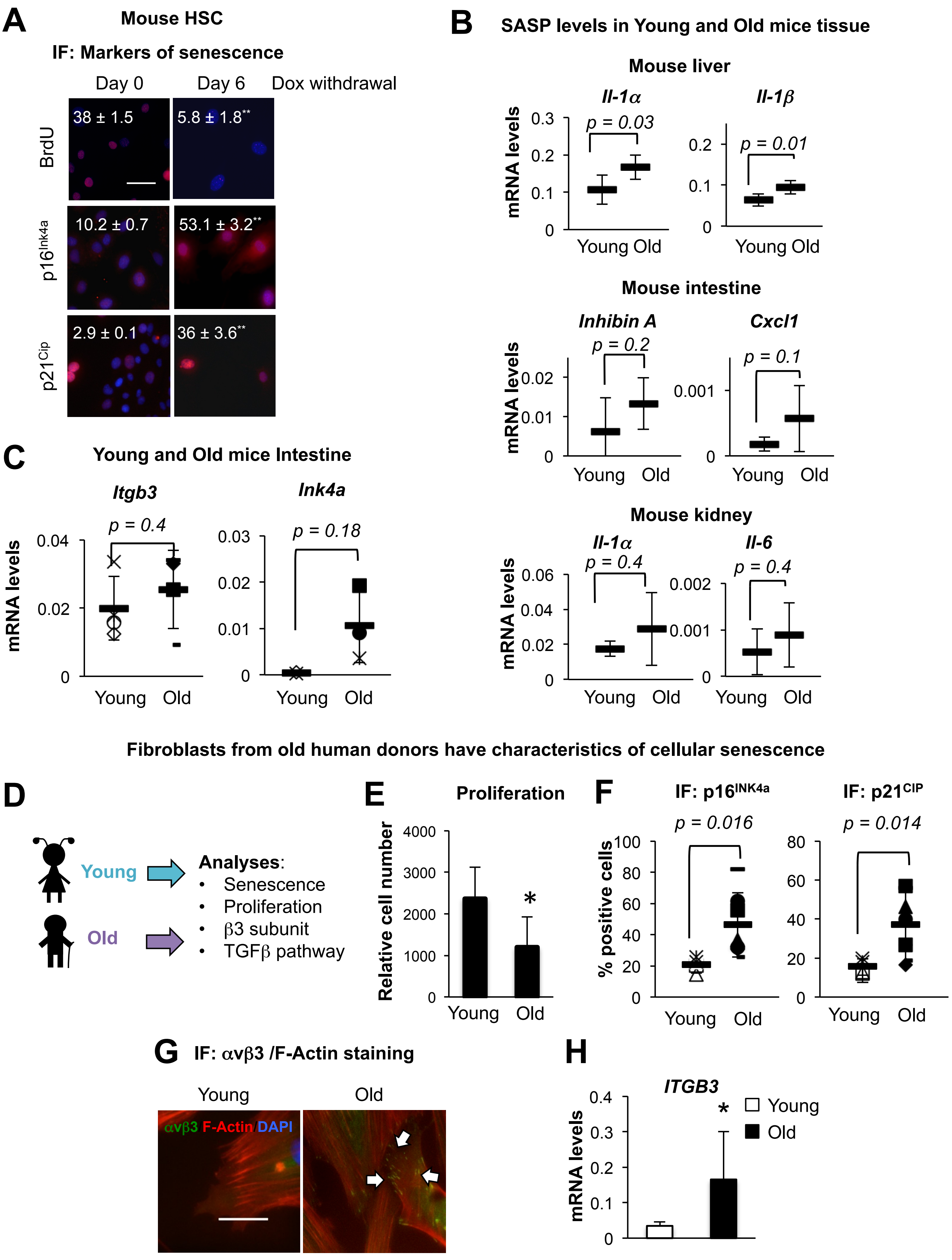

Figure S6. ITGB3 is expressed during replicative senescence and aging in human and mouse (Related to Figure 6)

(A) IF analysis for senescence markers (BrdU, $p16^{Ink4a}$ and $p21^{Cip}$) in mouse Hepatic Stellate Cells (mHSC) upon different days of doxycycline (Dox) withdrawal. Quantification represents the percentage of cells staining positive for each antibody. (B) Additional markers of senescence/aging - different SASP mRNAs - were determined in different tissues (liver, intestine, and kidney) from mice aged 4 (Young) and 25 (Old) months. (C) Intestines from young (4 months) and old (25 months) C57BL/6J mice were subjected to qPCR to determine *Ink4a* and *Itgb3* mRNA expression levels. (D) Schematic representation of the strategy followed to determine the implication of the β3 subunit in aging in human fibroblasts. We analyzed primary fibroblasts from young (∼10 years) and old (∼80 years) human donors to check for markers of senescence, β3 subunit and regulators of TGFβ. (E) Fibroblasts from old donors (n=7 donors) show a lower proliferation rate than fibroblasts from young donors (n=4 donors). (F) Fibroblasts from old donors present different markers for senescence. Graphs represent the percentage of cells stained positive for $p16^{INK4A}$ and $p21^{CIP}$ in young and old fibroblasts. Data represents the mean ± SD of fibroblasts derived from 4 young and 7 old donors. (G) Representative images for αvβ3 (green) and F-Actin (red) staining in young and old donor fibroblasts. The formation of FA is shown with white arrows in the old fibroblasts. Scale bar, 100 μm. (H) qPCR analysis for *ITGB3* mRNA levels in fibroblasts from young and old donors.

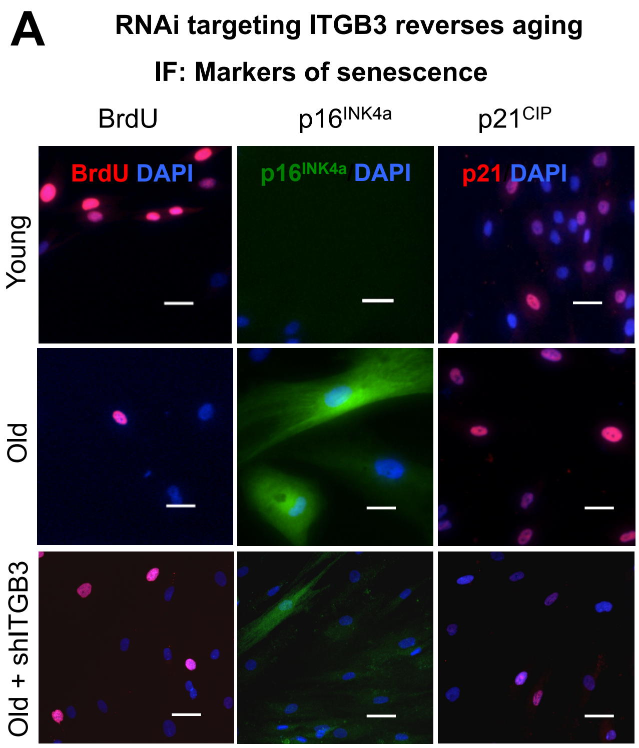
A
RNAi targeting ITGB3 reverses aging
IF: Markers of senescence
BrdU
p16INK4a
p21CIP
BrdU DAPI
p16INK4a DAPI
p21 DAPI
Young
Old
Old + shITGB3

Figure S7. Knockdown of *ITGB3* using a specific shRNA (shITGB3) averts senescence/aging markers in fibroblasts derived from old donors (Related to Figure7)
(A) IF analysis for different markers of senescence/aging (BrdU, $p16^{INK4A}$ and $p21^{CIP}$) in fibroblasts from old donors expressing an shRNA targeting *ITGB3* (shITGB3). Cells from young donors were used as controls.

SUPPLEMENTAL EXPERIMENTAL PROCEDURES

Antibodies for immunofluorescence, immunoblotting and ChIP.

Details of the antibodies used can be found in the Supplemental Table S2.

qPCR, immunoblotting and immunofluorescence

Total RNA was extracted using Trizol Reagent (Invitrogen) according to the manufacturer's protocol. cDNA was generated using the High Capacity cDNA Reverse transcription kit (Invitrogen). For specific primers see the Supplemental Table S2. Protein extracts were processed and analyzed as before (O'Loghlen et al., 2012). Immunofluorescence was performed using an InCell Analyzer 1000 (GE). Image processing and quantification was performed using InCell Investigator software (GE) (Acosta et al., 2008). Primers and antibodies used in this study are listed in the Supplemental Table S2.

RNA interference experiments

Indicated cells were transfected with 30nM of siRNA in a 96-well plate or 6-well plate. A 3.5% solution of HiPerFect transfection reagent (QIAGEN) was prepared in serum-free DMEM and then mixed with the siRNA. The mixture was incubated for 30 minutes at room temperature and then added to the cells. The medium was changed after 24 hours and cells were incubated for additional 24 hours before being processed for IF analysis or RNA/protein isolation. For RNAi targeting integrins, the forward transfection method was use, where the cells are plated first, senescence was induced and the siRNA/HiPerFect mixture was added after senescence was established. Experiments using an siRNA targeting p53 were reverse transfected, where the siRNA/HiPerFect mixture was added at the same time as cells were plated.

SA-β-Galactosidase staining

Cells were seeded at the same density and after 72h fixed with 0.5% Glutaraldehyde, washed with 1mM $MgCl_2$ pH 6.0 and stained with X-Gal staining solution (1mM $MgCl_2$ solution, 1X KC solution and X-Gal). Cells were incubated from 4h up to overnight at 37°C and stained with DAPI. The analysis of the percentage of SA-β-galactosidase positive cells was performed using the plugin Cell Counter in the ImageJ software. SA-β-Gal positive cells were determined as the percentage of cells staining blue (light or dark blue) with respect to the total amount of cells.

Chromatin immunoprecipitation (ChIP)

ChIP assay was performed using BF. Cells were cross-linked in 1% paraformaldehyde for 10 minutes at room temperature. Fixed cells were lysed in Lysis Buffer [50 mM HEPES (pH 7.5), 140 mM NaCl, 1 mM EDTA, 0.1% IGEPAL 630 (Sigma-Aldrich)], containing 0.05% Triton X100, 2.5 % glycerol and supplemented with 1X protease inhibitor cocktail (Roche) for 30 minutes on ice, followed by centrifugation incubation in Buffer 2 [0.1 M Tris HCl (pH 8) and 200 mM NaCl with protease inhibitors] for 30 minutes at room temperature. Chromatin was sonicated as follows: three cycles of 10 minutes each (30 seconds on followed by 30 seconds off). Crosslinked DNA after sonication was precipitated with 5 μg of anti-CBX7, anti-CBX8 and anti-RING1B antibodies or non-immune mouse/rabbit IgG (Abcam) overnight at 4°C. Chromatin/antibody complex was pulled down with Dynal Protein G or DynaI Protein A magnetic beads (Invitrogen) and washed in the low- and high-salt buffers. After de-crosslinking (65°C for 4 hours) and Proteinase K treatment, chromatin was purified by phenol-chloroform extraction and isopropanol precipitation. The antibodies used and qPCR primers are listed in the Supplemental Table S2.

SILAC (Stable Isotope Labeling with Aminoacids in Culture) Mass Spectrometry.

SILAC-labeled vector and shCBX7 fibroblasts were generated as before (O'Loghlen et al., 2012). Protein extracts were separated by SDS-PAGE and subjected to overnight in gel trypsin digestion. Peptide extracts were analyzed using a Q-Exactive mass spectrometer coupled to an Ultimate3000 LC (both

Thermo Fisher) using an Easy Spray Nano-source. The instrument was operated in data dependent acquisition mode selecting the 10 most intense precursor ions for fragmentation. Raw data was processed using MaxQuant/Andromeda as previously described (Cox and Mann, 2008). Outlier detection was performed using the significance B option available in Perseus software with a p-value cut-off of 0.05 for significance. Proteins were selected as significant when a two-fold difference in expression levels was observed. Proteomic data can be found in the Supplemental Table S1.

Pathways analysis KEGG

KEGG pathway analysis was performed on upregulated and downregulated proteins using DAVID Functional Annotation Bioinformatics Microarray Analysis (Huang da et al., 2009).

Mice tissue

All tissues (liver, kidney and intestine) come from female C57BL/6J mice. All mice (4, 19 and 25 month-old) were maintained in the same housing with identical environmental conditions. Tissues were provided by the Tissue Bank provider ShARMUK.

SUPPLEMENTAL REFERENCES

Cox, J., and Mann, M. (2008). MaxQuant enables high peptide identification rates, individualized p.p.b.-range mass accuracies and proteome-wide protein quantification. Nature biotechnology *26*, 1367-1372.

Huang da, W., Sherman, B.T., and Lempicki, R.A. (2009). Systematic and integrative analysis of large gene lists using DAVID bioinformatics resources. Nature protocols *4*, 44-57.

Table S1. Proteins differentially expressed upon shCBX7 (related to Figure 1)

Ratio H/L CB	Ratio H/L no	Ratio H/L CB	Ratio H/L no	PEP	Intensity	Unique pept	Unique pept	Ratio H/L co	Ratio H/L co	Intensity CB	Intensity L C	Intensity H C	Intensity CB	Intensity L C	Intensity H C	p-value FOR	p-value REV	Protein IDs	Protein nam	Gene names	Proteins
1.44631	0.951215	-2.49395	-1.74826	2.40E-63	8.53158	7	9	8	13	8.16035	7.62597	8.01026	8.29097	8.17397	7.66414	0.0292742	0.00089415	P00813;F5G\	Adenosine de	ADA	4
1.71048	1.49687	-1.93838	-1.44957	2.30E-31	7.97017	9	6	9	7	7.87365	7.27788	7.74659	7.26968	7.2029	6.42349	0.00229544	0.021418	Q9Y4K1;B4D	Absent in me	AIM1	9
2.23327	1.77167	-2.64647	-1.89421	8.65E-64	8.72874	10	13	9	13	8.17371	7.51204	8.06696	8.58692	8.45588	8.00268	7.67E-05	0.00020935	P12429;D6R/	Annexin A3;/	ANXA3	6
-1.62629	-2.02421	-2.09456	-1.43546	0	8.086	0	0	2	3	7.81066	7.69509	7.17924	7.7577	7.64607	7.11307	0.00398523	0.00590048	Q86X54;F8WDL0		AP1B1	2
2.37843	1.95832	-3.0517	-2.29301	7.19E-168	8.80721	7	13	6	13	8.32901	7.99498	8.05865	8.63166	8.58	7.68147	1.31E-05	7.60E-06	P02649;H0Y7	Apolipoprote	APOE	11
1.53297	1.05207	-1.85173	-1.29764	4.74E-17	7.85644	4	6	4	8	7.34604	6.78691	7.2058	7.69607	7.56633	7.10813	0.074796	0.0489709	Q8IUR7;A8M	Armadillo rep	ARMC8	17
3.09222	2.55748	1.62138	2.32984	9.01E-05	7.90019	1	1	1	1	7.70547	6.62175	7.66809	7.45808	6.80791	7.34805	2.11E-06	0.00130588	A8K5B0;Q9U	Armadillo rep	ARMCX3	2
2.08726	1.73231	-1.7238	-1.0803	1.49E-06	7.45596	1	2	1	2	6.53507	5.86674	6.43014	7.40045	7.25833	6.84621	0.0134794	0.0826409	A8K9U2;Q9N	Ankyrin repe	ASB6;DKFZp6	4
-1.72681	-2.17456	1.89426	2.51695	1.07E-42	8.13893	6	6	7	6	7.92982	7.81197	7.30576	7.72115	7.04879	7.61734	0.00183179	0.0005141	Q53HH2;P48	ATP synthase	ATP5O	5
1.52226	1.08393	-1.90363	-1.22061	1.12E-60	8.27063	3	4	5	7	7.83996	7.21131	7.72352	8.06934	7.94909	7.45283	0.0211275	0.0187458	A4D1N9;P07	Bisphosphog	BPGM	4
-3.90064	-4.44247	-4.2536	-3.56976	9.82E-45	7.93938	1	1	2	2	7.573	7.55297	6.22691	7.69515	7.68175	6.17791	3.85E-10	6.46E-08	Q4LE82;B0U.	Complement	C4A variant p	23
2.22435	1.63222	-1.70618	-1.1935	3.57E-05	6.84415	1	2	1	3	6.29743	5.56177	6.20922	6.69907	6.59719	6.01945	0.0188489	0.178892	Q8N3F0;B8Z.	UPF0452 pro	C7orf41	2
1.6427	1.05561	-3.22729	-2.57184	2.55E-48	7.94642	1	2	2	2	7.50453	6.78245	7.41322	7.75157	7.70671	6.74347	0.0335834	1.04E-06	Q9Y2V2;H3B	Calcium-regu	CARHSP1	5
2.1331	1.74507	-2.70369	-1.961	7.47E-34	8.47756	2	4	5	6	8.03989	7.31767	7.9486	8.28031	8.20809	7.46541	0.00047251	0.00020739	P42574	Caspase-3;Ca	CASP3	1
1.75719	1.27691	-1.79872	-1.13217	3.43E-12	7.9453	0	2	1	4	7.01068	6.35715	6.90163	7.89163	7.77428	7.26597	0.0555932	0.0289067	P55210;B4DU	Caspase-7;Ca	CASP7	5
2.97882	2.48063	-3.48684	-2.7314	3.29E-77	8.80789	8	10	9	11	8.33161	7.46762	8.26773	8.63137	8.56697	7.77075	4.07E-08	1.03E-07	A4D1G0;Q00	Cyclin-depen	CDK6	3
2.54374	2.00928	-3.65634	-3.00069	1.46E-86	8.30895	2	3	8	8	7.88309	7.10209	7.80447	8.10476	8.07936	6.85905	7.14E-05	2.07E-08	P42771;D1LY	Cyclin-depen	CDKN2A	7
-1.64395	-2.12218	-3.18298	-2.37864	3.60E-26	7.66251	1	1	1	1	7.00143	6.89175	6.35011	7.55559	7.50556	6.5922	0.0378447	0.00031347	Q14D22;Q9B	Charged mul	CHMP4A	6
1.58775	1.18948	-2.42314	-1.82741	0	9.51172	11	17	59	71	9.2257	8.62522	9.10023	9.19512	9.13373	8.31528	0.00554931	3.72E-06	P02452;H9C5	Collagen alph	COL1A1	5
1.55483	1.28865	-1.9155	-1.21424	6.23E-146	8.78116	12	16	14	20	8.39582	7.70126	8.2978	8.55071	8.21903	8.27827	0.00488328	0.0165462	D2JYH5;P024	Collagen alph	COL3A1	5
2.10802	1.74519	-1.96196	-1.20616	1.18E-17	7.86639	3	6	2	6	7.45082	6.68744	7.36862	7.65591	7.5499	6.99155	0.00087808	0.0671789	P05997;H7C1	Collagen alph	COL5A2	5
-1.58066	-2.18639	-2.51837	-1.80935	2.71E-05	6.72244	1	1	1	1	6.57009	6.3982	6.08443	6.19357	6.11398	5.41752	0.0316123	0.0469887	C9J8T6;Q140	Cytochrome	COX17	2
2.77093	2.27828	-4.21982	-3.4313	1.41E-21	7.74495	1	4	1	2	6.8779	5.06513	6.87117	7.68155	7.67391	5.92309	0.00169997	2.04E-07	A4D1M3;Q9U	Carboxypept	CPA4	8
2.29563	1.8143	-4.66863	-4.01059	2.33E-134	8.99599	11	8	30	20	8.77788	8.0187	8.6948	8.59238	8.56336	7.40286	5.46E-05	6.85E-15	P29373;Q5SY	Cellular retin	CRABP2	5
2.55144	2.2158	-1.90136	-1.21223	4.29E-06	6.87015	1	2	1	2	6.57726	5.55141	6.53429	6.56081	6.44837	5.91896	0.00219997	0.17266	Q9NZV1;B4D	Cysteine-rich	CRIM1	3
2.4522	1.88881	-3.26701	-2.58294	4.00E-39	8.82904	4	4	10	12	8.43072	7.59777	8.36173	8.60743	8.52916	7.8247	6.06E-05	4.77E-07	Q96CG8;G3V	Collagen trip	CTHRC1	4
1.94343	1.45586	-1.96607	-1.37142	6.26E-10	7.26274	2	1	2	1	7.0593	6.37694	6.95812	6.83563	6.73799	6.1396	0.018568	0.126087	Q9NTM9;Q5	Copper home	CUTC;RP11-4	3
1.67911	1.41868	-2.41519	-1.92994	1.00E-19	7.52304	5	3	4	1	7.4208	6.65859	7.33836	6.8448	6.82955	5.3827	0.02137	0.0346726	Q5T8F1;Q59	Death-associ	DAPK1	6
3.15903	2.64745	-1.619	-1.17251	3.15E-27	9.20868	3	1	4	1	9.2082	7.38707	9.20162	6.2447	6.1117	5.66596	3.29E-10	0.186076	Q58WW2;Q9	DDB1- and C	DCAF6;IQWD	3
2.83788	2.25396	-2.92128	-2.39585	7.72E-09	7.35866	1	2	1	3	6.67043	5.67817	6.6238	7.25902	7.20483	6.32832	0.00188068	0.00017803	Q96PD2;B7Z	Discoidin, CU	DCBLD2	2
1.67021	1.35541	-2.10843	-1.54768	2.19E-138	8.35593	17	22	14	21	8.09219	7.42606	7.98668	8.01406	7.90495	7.36082	0.00519926	0.00304045	Q96BY6;B3FI	Dedicator of	DOCK10;DOC	14
1.9871	1.53107	-3.2125	-2.58424	0.0011537	6.92005	1	1	1	1	6.51915	5.74767	6.43862	6.70017	6.66645	5.57344	0.0260902	0.00519812	Q9NRW4	Dual specifici	DUSP22	1
2.42315	1.83992	-2.44136	-1.59275	0	8.76288	7	10	10	14	8.43311	7.57646	8.36806	8.4888	8.4176	7.66842	9.12E-05	0.0017702	Q9NZN3;B4D	EH domain-c	EHD3	2
1.44932	1.08862	-1.83862	-1.18197	1.69E-115	8.83994	5	4	34	36	8.63843	8.05331	8.50769	8.40958	8.2917	7.78561	0.0156881	0.0195734	Q9HC35;B5M	Echinoderm	EML4	7
NaN	NaN	-3.01952	-2.46522	3.63E-90	7.27967	0	3	0	2	NaN	NaN	NaN	7.27967	7.23371	6.28165	1	0.000116	B1AHL2;B4DUV1;B1AHL4;I		FBLN1	11
2.03298	1.76507	-2.11166	-1.66004	6.79E-34	7.66539	3	4	3	2	7.49105	6.7616	7.40144	7.18475	7.14485	6.12798	0.00077501	0.00872761	Q4L180;C9JN	Filamin A-int	FILIP1L	3
2.39259	1.89619	-2.30579	-1.64079	1.57E-71	8.42334	7	9	7	11	8.17246	7.48959	8.0714	8.06562	7.96939	7.36391	2.40E-05	0.00170012	Q8NF12;Q13	Calcyphosin	FLJ00390;CA	3
1.94849	1.53262	-3.27286	-2.68529	1.29E-102	8.0887	7	9	5	9	7.89252	7.12672	7.81082	7.64917	7.57094	6.86623	0.00184928	4.75E-05	Q6ZNI9;H0Y2	Unconventio	FLJ00395;MY	8
1.91972	1.41792	-1.9578	-1.21961	7.24E-33	8.41427	6	10	12	11	7.95766	7.26224	7.85985	8.22755	8.12927	7.53394	0.00364971	0.0183846	Q12841;A8K5	Follistatin-rel	FSTL1	7
1.60051	1.32705	-1.86912	-1.27638	3.98E-23	7.20099	2	1	4	1	7.13994	6.47502	7.03411	6.31867	6.21809	5.6341	0.0298927	0.152544	Q86V85	Integral mem	GPR180	1
-1.54932	-2.09339	-6.11649	-5.41261	3.75E-20	8.19772	2	1	3	1	7.44846	7.31706	6.86523	8.1125	8.10745	6.17563	0.00475431	1.46E-23	P26583;Q5U	High mobility	HMGB2	3
1.67911	1.24513	-1.60007	-0.864596	0	9.34147	12	17	23	25	9.03926	8.37053	8.93444	9.04163	8.9144	8.44643	0.00486439	0.0581935	P54652;B3KU	Heat shock-r	HSPA2	2
-1.49716	-1.99666	-3.84801	-3.12759	2.68E-203	8.3069	1	1	1	1	7.99578	7.83775	7.48011	8.01574	7.97867	6.92858	0.00456946	3.12E-09	E7ESP4;E7EMF1;E9PB77		ITGA2	3
1.76333	1.45275	-1.78433	-1.16576	5.92E-174	9.20583	18	18	29	32	8.95275	8.34036	8.8312	8.85088	8.73343	8.22554	0.00111177	0.0102942	P26006;B4EC	Integrin alph	ITGA3	7
1.87606	1.50757	-1.97256	-1.33076	1.22E-212	8.91666	14	18	26	23	8.72009	8.06315	8.612	8.4778	8.33433	7.92702	0.00115089	0.00876077	P05106;B4D1	Integrin beta	ITGB3	11
-3.02938	-3.35004	-4.00666	-3.36943	7.55E-255	7.39023	0	0	2	1	7.35769	7.32461	6.22287	6.24886	6.235	4.74637	6.95E-06	0.0003023	H6VRG2;B4DL32;H0YI76;H		KRT1	4
2.85084	2.25988	-2.51911	-1.86828	2.53E-08	6.85424	1	2	1	2	6.44163	5.57737	6.37782	6.64189	6.58429	5.73608	0.00183517	0.0405739	Q9BT23	LIM domain-	LIMD2	1
1.67645	1.2506	-2.13397	-1.41001	6.50E-22	8.33457	5	7	6	10	7.7049	6.97585	7.61519	8.21848	8.11787	7.53404	0.0137057	0.0068303	P51884;Q53I	Lumican	LUM	3
1.56686	1.19415	-1.81345	-1.18164	0	9.32952	16	20	28	34	9.06251	8.44419	8.94288	8.99158	8.88576	8.3265	0.00679594	0.00928975	Q9UNF1;H7E	Melanoma-a	MAGED2	21
-2.18167	-2.30187	-1.72275	-0.830614	5.45E-15	7.59589	2	1	3	1	7.4128	7.32976	6.65333	7.13245	6.99914	6.55461	0.00272581	0.175879	Q14566;Q4Z	DNA replicati	MCM6	3
1.53595	0.949273	-1.99074	-1.30893	1.97E-177	8.90264	6	11	12	15	8.32836	7.79723	8.17696	8.76803	8.6707	8.07074	0.029587	0.0039136	B3KRC2;F5H1	Monoglyceri	MGLL	12
1.6352	1.16524	-1.60112	-0.822069	9.78E-182	8.83834	11	9	13	10	8.54388	7.95881	8.41313	8.53064	8.4101	7.9151	0.0102067	0.100874	Q9BV20	Methylthioril	MRI1	1
-1.70792	-2.09634	-2.10024	-1.48173	0	8.1805	1	1	2	2	7.90988	7.77479	7.33694	7.84678	7.74083	7.18219	0.00276256	0.00451143	D3DTH7		MYO1C	1
-1.35638	-1.6765	1.63483	2.35282	5.69E-09	6.59832	2	3	2	2	6.32934	6.18887	5.77075	6.26269	5.63341	6.14647	0.116031	0.0134001	B4DQP1;E7E	NADH dehyd	NDUFA13	5
1.80095	1.46148	-1.83331	-1.12828	5.36E-58	7.69407	4	3	4	3	7.42719	6.79281	7.31252	7.35597	7.23565	6.73972	0.018174	0.0704551	Q9C002;H0Y	Normal muc	NMES1;C15o	2

-1.41074	-1.77448	1.81824	2.38225	7.70E-28	7.71423	5	3	6	3	7.64488	7.50598	7.08221	6.88326	6.1813	6.7871	0.0184398	0.0122381	Q13423;Q2T[illegible]	NAD(P) trans	NNT	9
1.51485	1.29748	-2.01526	-1.37778	1.24E-17	7.16587	2	4	1	4	6.4442	5.82379	6.32521	7.07445	6.98452	6.34641	0.052458	0.0284737	Q13219;B4D	Pappalysin-1	PAPPA	6
-1.40354	-1.71227	-1.82941	-1.4079	5.32E-07	7.13906	2	3	2	2	6.88335	6.67619	6.46241	6.78743	6.67758	6.13669	0.106926	0.116938	Q3YEC7;G8JL	Rab-like prot	PARF;C9orf8	10
2.61565	2.08257	-2.34512	-1.57248	0	9.65351	0	0	42	68	9.27114	8.47614	9.19526	9.42095	9.32775	8.70679	8.48E-07	7.72E-05	B4DRT3;E7E[illegible]	Pyruvate kin	PKM2	3
NaN	NaN	-2.93832	-2.11866	4.13E-219	7.41868	0	1	0	2	NaN	NaN	NaN	7.41868	7.36524	6.48237	1	0.00132424	E7ETU9;B4DHG3;B3KWS3;		PLOD2	6
1.82244	1.25635	-2.42973	-1.80591	8.57E-300	8.78436	8	9	13	11	8.46112	7.82859	8.3459	8.50444	8.42886	7.70788	0.00595468	0.00040434	B2R6X6;P30	Peptidyl-prol	PPIF	13
2.55159	2.20402	-2.89218	-2.19621	1.07E-53	8.34974	6	9	10	16	7.9222	6.99217	7.86792	8.14656	8.08714	7.25324	1.55E-05	3.49E-05	P26022	Pentraxin-rel	PTX3	1
1.69737	1.27471	-1.71317	-1.01162	1.55E-63	8.56378	3	4	15	15	8.45643	7.80347	8.34721	7.90421	7.76884	7.33201	0.00532229	0.0501373	Q92930;H0Y	Ras-related p	RAB8B	10
2.47902	2.01489	-3.0308	-2.37452	6.72E-48	8.14891	2	5	2	4	7.23277	6.4755	7.14931	8.09276	8.03687	7.17464	0.00165224	8.05E-06	A8K3J8;P550	GTP-binding	RRAD	5
1.37995	0.823749	-2.22553	-1.57192	3.53E-82	9.46984	6	5	66	53	9.23454	8.69557	9.08636	9.09132	9.00711	8.33754	0.0584186	0.0005176	D3DV26;P60	Protein S100	S100A10	3
1.56866	1.1036	-1.95137	-1.23416	1.06E-07	6.91916	2	1	2	1	6.85284	6.26309	6.72372	6.0703	5.96909	5.38803	0.0886865	0.165572	P29034;Q5RI	Protein S100	S100A2	2
1.69995	1.14841	-2.09486	-1.45918	1.88E-40	7.88545	6	5	6	6	7.64803	7.04187	7.52445	7.50986	7.41469	6.80391	0.0222084	0.0268856	P33764	Protein S100	S100A3	1
1.83435	1.47566	-2.50611	-1.83259	2.04E-224	9.46046	2	2	37	43	8.8674	8.12898	8.77982	9.33248	9.24878	8.57624	0.00093411	3.48E-06	B2R7Y0;H7BYS2;H7C004;E		SERPINB10	4
2.74966	2.17702	-3.35868	-2.65608	1.87E-169	8.14882	2	1	4	2	7.91954	7.04021	7.85804	7.76178	7.72702	6.64779	1.92E-05	4.62E-07	P05120;E7EP	Plasminogen	SERPINB2	3
2.31136	1.79086	-2.21632	-1.49387	9.21E-29	8.32595	7	6	8	7	7.96501	7.26724	7.8678	8.07755	7.95839	7.45761	0.00034582	0.00419985	P05121;F8W	Plasminogen	SERPINE1	7
1.50294	0.987757	-2.43893	-1.48201	1.34E-184	8.72054	11	18	17	20	8.20542	7.60987	8.07828	8.56227	8.48048	7.79689	0.0238949	0.00358171	Q9H788;H0Y	SH2 domain-	SH2D4A	2
-1.82337	-2.19106	2.06533	2.77964	1.28E-18	7.90064	3	5	3	5	7.10195	7.01812	6.34633	7.82545	7.28344	7.6785	0.00452962	3.99E-05	D9HTE9;Q6L[illegible]	Tricarboxylat	SLC25A1	4
1.70867	1.40746	-1.65676	-1.18761	5.37E-06	6.97997	1	1	1	1	6.79385	6.1002	6.6956	6.52222	6.39291	5.93303	0.0380997	0.180887	B7ZLQ5;P283	Probable glol	SMARCA1	7
1.67224	1.20069	-2.01841	-1.24646	0	9.49624	19	24	30	43	9.0844	8.3987	8.98408	9.28341	9.16679	8.65549	0.00651447	0.00199033	P52788;B4DE	Spermine syr	SMS	3
1.43563	0.91311	-2.16961	-1.495	4.22E-10	7.33157	3	2	4	2	7.14476	6.58583	7.00445	6.87512	6.77938	6.17143	0.113026	0.0971982	P37840;H6U	Alpha-synucl	SNCA	6
2.93172	2.45375	-3.74101	-2.83516	1.26E-12	7.02284	3	3	3	3	6.80456	5.65401	6.7727	6.61954	6.55229	5.77626	0.00080037	0.00224189	O76070;Q6F	Gamma-synu	SNCG	4
1.63338	1.32343	-1.80682	-1.18187	3.57E-06	7.37146	2	1	3	1	7.19769	6.54683	7.08789	6.8896	6.82061	6.05664	0.0302831	0.182846	D2JYI1;A3QN	TGF-beta rec	TGFBR2	5
1.4197	1.00813	-3.20051	-2.67355	0.00028262	6.86025	1	1	1	1	6.56071	5.98134	6.42792	6.55775	6.52842	5.3726	0.112813	0.00388153	Q96HP8;H7C	Transmembr	TMEM176A	4
-1.58701	-2.04258	1.98939	2.6893	4.54E-09	7.79993	2	2	3	2	7.63155	7.47387	7.11508	7.30698	6.61731	7.20772	0.00597974	0.00024261	Q99785;E9PI	Tumor prote	TP53I11	13
1.49978	0.965175	-2.01392	-1.36702	4.00E-233	9.25249	16	24	25	45	8.66165	8.10332	8.5211	9.12375	9.0089	8.48994	0.0299951	0.00257331	Q16222;B1A	UDP-N-acety	UAP1	2
-1.52891	-1.65381	-1.9143	-1.2137	1.38E-06	7.03104	1	1	1	2	6.20874	6.06187	5.66644	6.96019	6.84334	6.3329	0.122118	0.172177	P63146;H0Y	Ubiquitin-co	UBE2B	3
1.72583	1.49093	-3.07534	-2.31058	0.00046673	7.19218	1	1	1	1	7.06002	6.38714	6.95633	6.61111	6.55672	5.68195	0.0162255	0.0121038	P17029;B3K	Zinc finger pr	ZKSCAN1	5

Table S2. List of reagents used in this study (Related to Figures 1-7)

Primer sequences for qPCR analyses used in this study

HUMAN		
**Target**	**Forward primer**	**Reverse primer**
RPS14	CTGCGAGTGCTGTCAGAGG	TCACCGCCCTACACATCAAACT
INK4A	CGGTCGGAGGCCGATCCAG	GCGCCGTGGAGCAGCAGCAGCT
TP53	TGGCCATCTACAAGCAGTCA	GGTACAGTCAGAGCCAACCT
CBX7	AACTCCATCACCGTCACCTT	CCCCAACCCATCCCTATCTC
NDUFA13	CTACGGGCACTGGAGCATAA	AGCGGGTTGTGTGGAACA
S100A3	CAGATTGGTAAACACCCGAAC	ACAAAGTCCACCTCGCAGTC
TGFBR2	CGGCTCCCTAAACACTACCA	TATGTCACCCACTCCCTGCT
AIM1	TCCCCAGAAAGTGAAGGAAA	TGTTGGAAGAGCAGCGTATG
DUSP22	GAAAGGGGAGTGTGGCTGTA	GCGGCTGTGAAGAAAGAACA
RAB8B	GCACATCAGTGTTAGCCTTTCC	TGAACCAGACCAAATACCCTTT
NNT	GATACTGGGTTTGGGCATTG	CCCTGAGAAGTTGTGGAAGG
ITGB3	GGGGTAGGTTGGGAGAATGT	TCTGGGACAAAGGCTAAGGA
SMS	GCACAGCGAAGACTGCTAAA	GGGGAAAGAAACACCATCAA
SNCA	TTCTGGGGCATAGTCATTTCT	TTCCTCCTTCCTTCCTCACC
ZKSCAN1	CCATTTCCCCCTTTTGTTTC	TGCGTGTGCTTTTCTCTTGT
ITGA2	GGATTTGTTTGGCTGACTGG	GATAACTTTGGACCGCTGGA
EHD3	CCCACCACAGACTCCTTCAT	GCTCTCCAGCACAGGGTTAG
MAGED2	CAGGCATACTGGGAACGACT	GCTATTGGGGACTCTGGCATA
S100A2	AAGAGGGCGACAAGTTCAAG	TGATGAGTGCCAGGAAAACA
ARMC8	TCCTCTCCACTCGTCCTCAT	GTTGTCCCATCCGCTATGTT
DAPK1	GAAGCAAGGGGGTGATGTAG	CCACAGACAACGGAATGAGA
SH2D4A	ATGCCCTGTCCTATCTGTCG	TGGAGGCTGTCACTCAAACA
FILIP1L	AAACGCCTCCATAACACCAG	AACCAGTCACAGCCAAAACC
GRO alpha	GAAAGCTTGCCTCAATCCTG	CACCAGTGAGCTTCCTCCTC
GCP2	AGAGCTGCGTTGCACTTGTT	GCAGTTTACCAATCGTTTTGGGG
CCL2	AGCTCGCACTCTCGCCTCCAG	GGCATTGATTGCATCTGGCTGAGC
IL-1 beta	TGCACGCTCCGGGACTCACA	CATGGAGAACACCACTTGTTGCTCC
IL-8	GAGTGGACCACACTGCGCCA	TCCACAACCCTCTGCACCCAGT
IL-6	CCAGGAGCCCAGCTATGAAC	CCCAGGGAGAAGGCAACTG
IL-11	CCTGGCTCTTCCCCATCTAG	CAGCTCTCAGACAAATCGCC
TGFB1	TAC TAC GCC AAG GAG GTC AC	GCT GAG GTA TCG CCA GGA AT
TGFB2	TTT GGA AGT TTG TGT TCT GTT TG	TGT TGT TGT TGT CGT TGT TCA C
TGFB3	ACA CAC AAG CAA CAA ACC TCA C	TCC TCT AAC CAA ACC CAC ACT T
SMAD2	GCAAAAAGGTTCACACTAAAGGA	AGCAAAGGTTGAGGAAGGAGATA
SMAD3	TGATGTTAGAGGGAGATGGAGAG	GAGGAAGTGAGGGGTTTTGTATT
SMAD4	ACCCCAGCTCTGTTAGCCCCA	TGGCAGGCTGACTTGTGGAAGC
TGFBR1 (ALK5)	TCTGCCACAACCGCACTGTCA	GGTAAACCTGAGCCAGAACCTGACG
LTBP1	GAG TGC TGC TGT CTG TAT GGA G	AAACGGTCTTGGATGAAGTAGG
LTBP3	GGA GGA GAA GAG CCT GTG TTT	GTG GGA GGT GAG AAT GTG GTA T
CDKN2B (p15INK4B)	ACCAGATAGCAGAGGGGTAAGAG	GTGTGTGTGTGTGTGTGTGAAAG
CDKN1B (p27KIP)	CCAGTCCATTTGATCAGCGG	ACATCTTTCTCCCGGGTCTG
MMP1	CAAATGCAGGAATTCTTTGGGC	GTAGGTCAGATGTGTTTGCTCC
MMP9	AACTTTGACAGCGACAAGAAGT	ATTCACGTCGTCCTTATGCAAG
ITGB1	TGTGGTTGCTGGAATTGTTCTT	ATTCAGTGTTGTGGGATTTGCA
ITGB2	ATGTGGATGAGAGCCGAGAG	ACTGGGACTTGAGCTTCTCC
ITGB4	ACTACACCCTCACTGCAGAC	TCTGGCTTGCTCCTTGATGA
ITGB5	AACCAGAGCGTGTACCAGAA	AGGAGAAGTTGTCGCACTCA
ITGB6	GAAGGGGTGACTGCTACTGT	TGCACACACATTCACCACAG
ITGB7	AAGTTGGGCGGCATTTTCAT	CCCCAACTGCAGACTTAGGA
ITGB8	CCCAGAATCACTCCAACCCT	GTGAACCCTAATTGCGCCAT
**MOUSE**		
Ink4a	GTGTGCATGACGTGCGGG	GCAGTTCGAATCTGCACCGTAG
Rps14	GACCAAGACCCCTGGACCT	CCCCTTTTCTTCGAGTGCTA
Itgb3	AACCACTACTCTGCCTCCAC	ACTGTGGTCCCAGGAATGAG
Tp53	AAACGCTTCGAGATGTTCCG	GTAGACTGGCCCTTCTTGGT
Cdkn1a (p21CIP)	TCCCGACTCTTGACATTGCT	TGCAGAAGGGGAAGTATGGG
Cxcl1	CTGGGATTCACCTCAAGAACATC	CAGGGTCAAGGCAAGCCTC
Inhibin A	GATCATCACCTTTGCCGAGT	TGGTCCTGGTTCTGTTAGCC
Il-1 alpha	CGCTTGAGTCGGCAAAGAAAT	TGGCAGAACTGTAGTCTTCGT
Il1- beta	TGCCACCTTTTGACAGTGATG	TGATGTGCTGCTGCGAGATT
Il-6	TGATTGTATGAACAACGATGATGC	GGACTCTGGCTTTGTCTTTCTTGT
Cbx7	TGCGGAAGGGCAAAGTTGAAT	ACAAGGCGAGGGTCCAAGA

Primer sequences for ChIP-qPCR analyses used in this study

Target	Forward primer	Reverse primer
ARF	GTGGGTCCCAGTCTGCAGTTA	CCTTTGGCACCAGAGGTGAG
ACTB	CCGTTCCGAAAGTTGCCTT	CGCCGCCGGGTTTTATA
INK4A	ACCCCGATTCAATTTGGCAG	AAAAAGAAATCCGCCCCCG
ITGB3 TSS	CTGAAGACACGTGCCCAAGG	TCCGTCTCTAACCTGGAAGTCC
ITGB3 Coding	GTGCAGTGGTGTGATCCCTG	AGCATTTTGGGAAGCCGAGG
SNCA	GGAGTCGGAGTTGTGGAGAA	GGGACAAGTACTCACCTCCC
ZKSCAN	AAAAGTAAATCTGGCCGGGC	TCCCAGGTTCAAGCGATTCT
S100A2	CCCATCCTTCCAGACACCTT	TGAGAGAGAAGCAACCTGGG
ITGA2	TGCTGGAAATTTGTGGCCAA	TGAGAGCCCATACATGCACT
ARMC8	AAACTCCCAGTGCCTGTCTT	ATGGGGCAGAACATAACCCT
DUSP22	CCGCTGACTTGTTGACACTG	TGTTCATCCCATTCCCCATG
S100A3	GAAGGGGACAGTGGAAGTGG	AAGTTGGGGTTCATCTCACC
TGFBR2	GCGCTGAGTTGAAGTTGAGT	AGATGTGCGGGCCAGATG
SH2D4A	GTCTCCTTCCTTCCAGCCTT	ATCTGCAGATCTGGGCCTTT
AIM1	CGGTCGTGATTACTCCCAGA	CCCGCGGAGATTTCACTTTC
FILIP1L	CAAAGGTGGAAGGTGCACTC	TCCCAAATCCCATCCTTCCC
RAB8B	CTCTCCACCGCCTCCTCT	GGTGGAGATGAAGGTGGTGT
DAPK1	CTTCGGAGTGTGAGGAGGAC	GGGAACACAGCTAGGGAGTG

RNAi sequences used in this study

siRNA	
**Target**	**Sequence**
siTP53_7	CAGCATCTTATCCGAGTGGAA
siITGB3_3	CTCTCCTGATGTTAGCACTTAA
siITGB3_4	CAAGCTGAACCTAATAGCCAT
siITGB3_5	CACGTGTGGCCTGTTCTTCTA
siTGFBR2_2	TCGCTTTGCTGAGGTCTATAA
siTGFBR2_7	TCGGTTAATAACGACATGATA
**shRNA**	
**Target**	**Sequence**
TP53	GTAGATTACCACTGGAGTC
CBX7	CGGAAGGGTAAAGTCGAGT
ITGB3	GATGCAGTGAATTGTACCTAT

Antibodies used in this study

Target	Catalogue n. - Clone	Application	Concentration
CBX7	Ab21873	ChIP, WB	1/200
CBX8	A300-882A	ChIP	1/200
RING1B	Ab3832	ChIP	1/200
IgG	Ab18443 - MOPC21	ChIP	1/200
p16INK4A	sc-56330 - JC-8	WB	1/200
β-tubulin	sc-9104	WB	1/500
ITGB3 or β3	Ab179473 - ERP17507	WB, IF	1/200
β-Actin	sc-47778	WB	1/500
p21CIP	Ab109520	IF	1/200
BrdU	A21303	IF	1/200
αvβ3	LM609	blocking Ab	10μg/ml
p53	sc-126 - DOI	IF	1/200
Pan TGFB 1-3	AB-100-NA	blocking Ab/WB	10μg/ml
IgG blocking antibodies	ab18413	blocking Ab	10μg/ml
pST/Q	9607	IF	1/200
Ki67	ab15580	IF	1/500
SMAD2/3	5678S	IF	1/200
CXCL6/IL-6	AB-206-NA	WB/IF	1:250/1:200
CXCL8/IL-8	6217	WB/IF	1:500/1:200

Inhibitors used in this study

Name	Target	Catalogue n.	Concentration
PD98059	MEK1/2	99005	40 μM
SB202190	p38MAPK	sc-222294	20 μM
Torin-2	mTOR	14185	100 nM
Tgf-B Ri Kinase Inhibitor I	Tgf-B Ri Kinase Inhibitor I	61645	4 μM
Vegfr-2/Flt3/C-Kit	Vegfr-2/Flt3/C-Kit	676500	8 μM
Etoposide	DNA damage	341205	100 μM
PD0332991 (Palbociclib)	CDK4/6	A8316	200 nM
GSK429286A	ROCK1/2	Ab1466581	150 nM
ILK Inhibitor, Cpd22	ILK	40733	50 nM
Cilengitide	αvβ3/αvβ5	A12372	50 nM
