# Supplementary material for: Integrin Beta 3 Regulates Cellular Senescence by Activating the TGF-β Pathway
Source: Cell Rep. 2017 Mar 7;18(10):2480–93. doi: 10.1016/j.celrep.2017.02.012 (PMC5357738; doi:10.1016/j.celrep.2017.02.012)
Supplement: Document S2. Article plus Supplemental Information [file mmc2.pdf]

Cell Reports

Article

Integrin Beta 3 Regulates Cellular Senescence by Activating the TGF-β Pathway

Graphical Abstract

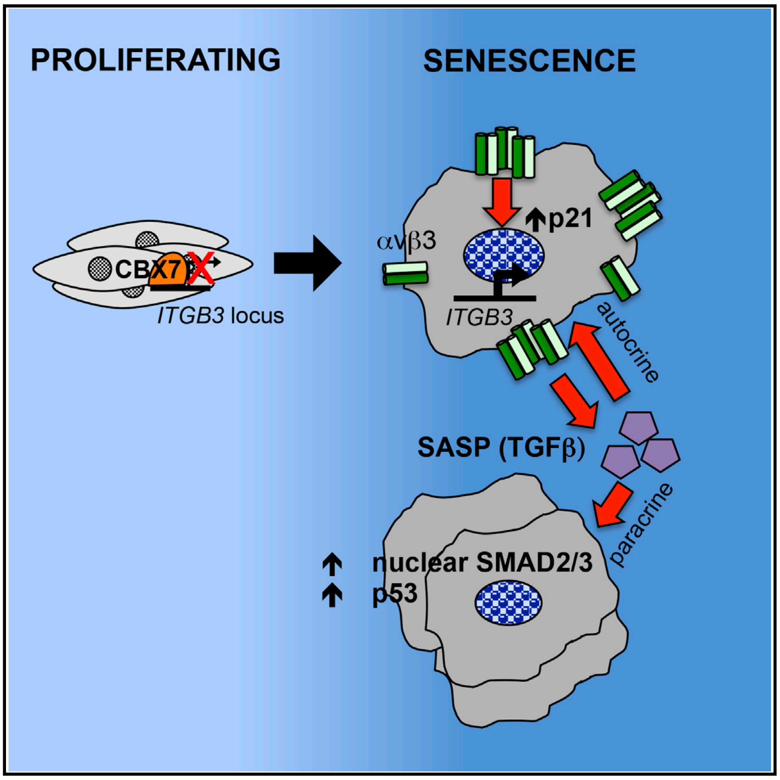

Highlights

- *ITGB3* (integrin β3) is regulated by the Polycomb protein CBX7
- β3 regulates senescence by activating TGF-β in a paracrine and autocrine fashion
- β3 is highly expressed in OIS and induces senescence via ligand-independent pathway
- There is a positive correlation between β3 levels and aging in different tissues

Authors

Valentina Rapisarda, Michela Borghesan, Veronica Miguela, Vesela Encheva, Ambrosius P. Snijders, Amaia Lujambio, Ana O'Loghlen

Correspondence

a.ologhlen@qmul.ac.uk

In Brief

Rapisarda et al. show that integrin β3 subunit expression induces senescence by activating TGF-β, while β3 knockdown overcomes senescence. β3 is dynamically upregulated in OIS and has ligand-independent activity. They also find a positive correlation between β3 levels and aging in a subset of tissues.

Accession Numbers

PXD005717

CrossMark
Rapisarda et al., 2017, Cell Reports *18*, 2480–2493
March 7, 2017 © 2017 The Author(s).
http://dx.doi.org/10.1016/j.celrep.2017.02.012

CellPress

OPEN ACCESS
CellPress

Cell Reports
Article

Integrin Beta 3 Regulates Cellular Senescence by Activating the TGF-β Pathway

Valentina Rapisarda,[1] Michela Borghesan,[1] Veronica Miguela,[2] Vesela Encheva,[3] Ambrosius P. Snijders,[3] Amaia Lujambio,[2] and Ana O'Loghlen[1,4,*]
[1]Epigenetics & Cellular Senescence Group, Blizard Institute, Barts and The London School of Medicine and Dentistry, Queen Mary University of London, 4 Newark Street, London E1 2AT, UK
[2]Department of Oncological Sciences, Icahn School of Medicine at Mount Sinai, 1470 Madison Avenue, New York, NY 10029, USA
[3]Protein Analysis and Proteomics Group, The Francis Crick Institute, South Mimms EN6 3LD, UK
[4]Lead Contact
*Correspondence: a.ologhlen@qmul.ac.uk
http://dx.doi.org/10.1016/j.celrep.2017.02.012

SUMMARY

Cellular senescence is an important in vivo mechanism that prevents the propagation of damaged cells. However, the precise mechanisms regulating senescence are not well characterized. Here, we find that *ITGB3* (integrin beta 3 or β3) is regulated by the Polycomb protein CBX7. β3 expression accelerates the onset of senescence in human primary fibroblasts by activating the transforming growth factor β (TGF-β) pathway in a cell-autonomous and non-cell-autonomous manner. β3 levels are dynamically increased during oncogene-induced senescence (OIS) through CBX7 Polycomb regulation, and downregulation of β3 levels overrides OIS and therapy-induced senescence (TIS), independently of its ligand-binding activity. Moreover, cilengitide, an αvβ3 antagonist, has the ability to block the senescence-associated secretory phenotype (SASP) without affecting proliferation. Finally, we show an increase in β3 levels in a subset of tissues during aging. Altogether, our data show that integrin β3 subunit is a marker and regulator of senescence.

INTRODUCTION

Cellular senescence is characterized by a proliferative arrest induced to prevent the propagation of damaged cells in a tissue. This arrest is mainly driven by the activation of two important pathways, p53/p21^{CIP} and RB/p16^{INK4A}. The senescence program can be triggered by a number of stressors, like the activation of oncogenes (termed oncogene-induced senescence; OIS), drug treatment (therapy-induced senescence; TIS) or deregulation of Polycomb Repressive Complex 1 (PRC1) proteins, including the polycomb protein chromobox 7 (CBX7) (Gil and O'Loghlen, 2014; Salama et al., 2014). Although arrested, senescent cells are metabolically and transcriptionally functional, and they actively communicate with their surroundings (Pérez-Mancera et al., 2014). In fact, senescent cells secrete an array of inflammatory proteins, growth factors, and metalloproteases that collectively constitute the SASP (senescence-associated secretory phenotype). The SASP recruits the immune system in order to eliminate senescent cells and induces changes in the extracellular matrix (ECM), thus facilitating tissue homeostasis and regeneration. The presence of senescent cells has been found in vivo in preneoplastic lesions (Muñoz-Espín and Serrano, 2014), in wound healing (Jun and Lau, 2010), during embryonic development (Muñoz-Espín et al., 2013; Storer et al., 2013), and in different tissues throughout aging (Baker et al., 2016; Krishnamurthy et al., 2004; Ressler et al., 2006). Interestingly, a recent study has demonstrated that p16^{INK4A}-positive cells accumulate during aging and contribute to age-related dysfunctions in different tissues. Thus, the elimination of senescent cells reverses the aging phenotype and stimulates tissue regeneration, demonstrating that the activation of senescence is a direct cause of aging and opening avenues for targeting senescent cells as a therapy to extend healthy lifespan (Baker et al., 2016).

Senescence activation is governed by intracellular and extracellular signals and highly depends on the interaction of the cells with ligands in the ECM (Gutiérrez-Fernández et al., 2015; Jun and Lau, 2010; Parrinello et al., 2005). The most common ECM-receptor interaction proteins are integrins, which are heterodimeric cell-surface transmembrane receptors that provide cellular adhesion (Hynes, 2002). Upon ligand binding, intracellular proteins are recruited to clusters of integrin heterodimers in the plasma membrane, forming focal adhesion (FA) complexes, which mediate downstream signals to the cell. Integrin signaling affects numerous cellular processes, including cell adhesion, migration, proliferation, survival, and differentiation (Seguin et al., 2015) and, thus, has key roles during development, immune responses, and different pathologies such as cancer (Legate et al., 2009). Interestingly, integrins can also mediate signaling cascades independent of ligand binding. In fact, integrin αvβ3 can induce apoptosis (Stupack et al., 2001), tumor progression (Desgrosellier et al., 2009), and tumor stemness (Seguin et al., 2014) in an anchorage-independent manner.

Here, we establish integrin beta 3 (β3) subunit as a marker and regulator of senescence. Our findings highlight the importance of the β3 subunit signaling in regulating senescence by activating the transforming growth factor β (TGF-β) pathway in an autocrine and paracrine fashion. We found that β3 levels are dynamically upregulated during OIS, by CBX7 transcriptional regulation,

 © 2017 The Author(s).
This is an open access article under the CC BY license (http://creativecommons.org/licenses/by/4.0/).

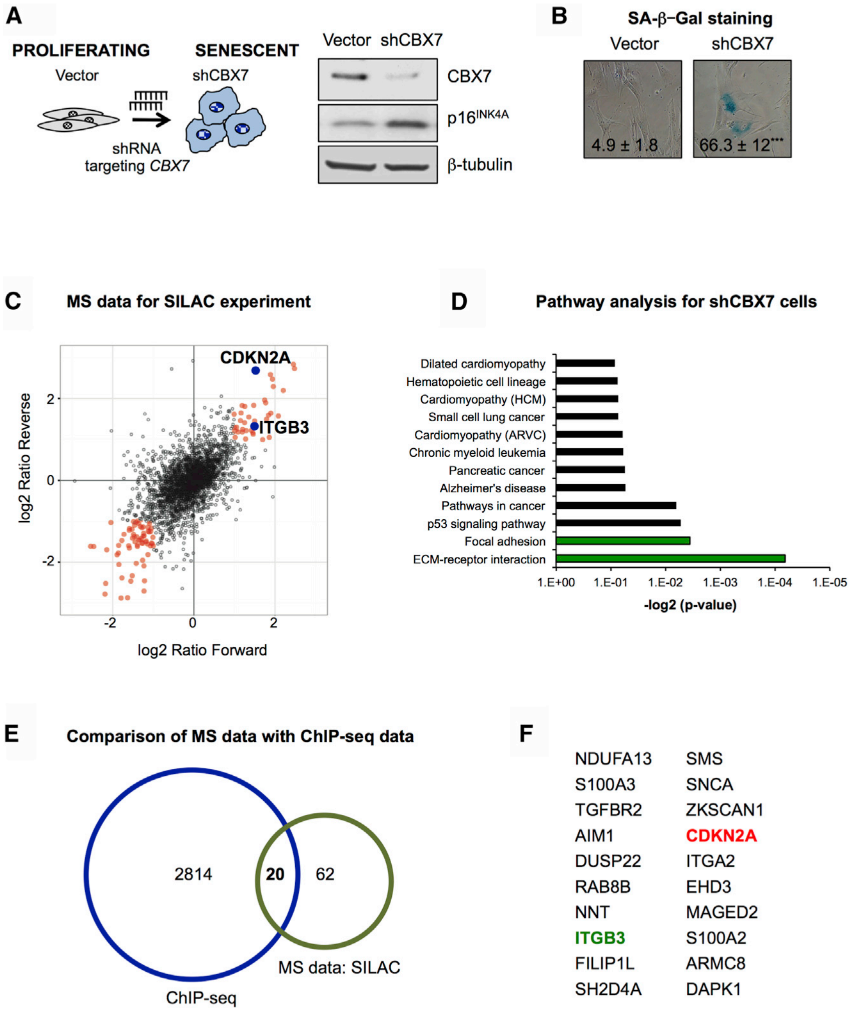

Figure 1. SILAC Screen Identifies Putative Regulators of Senescence

(A) Left panel: schematic representation of the senescence model used in the SILAC screen. Human primary breast fibroblasts (BFs) were transduced with a lentivirus harboring an shRNA targeting *CBX7* (shCBX7). Right panel: immunoblot showing CBX7 knockdown efficiency and an increase in p16^{INK4A} protein levels. β-tubulin is used as loading control.

(B) Senescence induced upon shCBX7 is shown by an increase in the percentage of cells staining positive for SA-β-galactosidase (SA-β-Gal) activity. Quantification of two to three independent experiments is shown.

(C) Scatterplot of mass spectrometry (MS) results from both forward and reverse SILAC experiments. A 2-fold difference in expression upon shCBX7 is indicated with orange circles, outlining CDKN2A and ITGB3 in blue. Gray circles represent unchanged proteins.

(D) Pathway analyses (KEGG) show that proteins with a 2-fold difference in expression fall within the categories of the extracellular matrix (ECM)-interacting and FA pathways.

(E) Comparison of the proteins significantly deregulated in the SILAC experiment with a published dataset for genes regulated by CBX proteins in human diploid fibroblasts (Pemberton et al., 2014).

(F) List of 20 proteins in the SILAC screen whose genes could be regulated by CBX proteins, highlighting CDKN2A (red) and ITGB3 (green).

and that β3 regulates OIS independently of its ligand-binding activity. Importantly, an αvβ3 antagonist exerts dual activity by regulating interleukin (IL)-6/IL-8 secretion but not the growth arrest during OIS. Additionally, we found an increase in the levels of β3 during aging in a subset of mouse tissue and human samples, where the manipulation of β3 levels in fibroblasts derived from old human donors overcomes the accumulation of different markers of senescence and aging. Our results demonstrate the importance of cellular adhesion during senescence and identify integrins as potential therapeutic targets during early carcinogenesis and aging.

RESULTS

SILAC Screen Identifies Proteins Grouped in the ECM-Receptor Interaction Pathway as Putative Regulators of Senescence

We have previously shown that CBX7 loss of function by short hairpin RNA (shRNA) in primary cells induces cellular senescence (Gil and O'Loghlen, 2014; O'Loghlen et al., 2015a, 2015b) (Figures 1A and 1B). This has been primarily attributed to the derepression of the *CDKN2A* locus, which encodes the cell-cycle inhibitor p16^{INK4A}. As PRC1 targets multiple genes, and we see additional markers of senescence present upon *CBX7* knockdown (Figures 1B and S1A), we hypothesized that other unknown regulators could be inducing senescence in this model. To this end, we performed a quantitative proteomic analysis to determine changes occurring upon *CBX7* knockdown in human primary fibroblasts, taking advantage of the SILAC (stable isotope labeling by amino acids in culture) technology. Human primary breast fibroblasts (BFs) transduced with an shRNA targeting *CBX7* (shCBX7) were grown in media supplemented with "heavy" (forward experiment)- or "light" (reverse experiment)-labeled amino acids and compared with BFs expressing an empty vector (Figure S1B). Combination of the results from both forward and reverse experiments show 82 proteins with a 2-fold expression difference in both experiments, including CDKN2A (Figure 1C). Annotation of differentially expressed proteins into functional pathways (Kyoto Encyclopedia of Genes and Genomes; KEGG) shows the ECM-receptor-interacting and focal adhesion pathways upregulated upon *CBX7* knockdown (Figure 1D).

As the SILAC screen was performed in a CBX7-depleted background, we hypothesized that the genes encoding the proteins found in the SILAC screen could be regulated by CBX7. Thus, we compared the SILAC data with a published

genome-wide binding profile for CBX proteins (chromatin immunoprecipitation sequencing; ChIP-seq) in human fibroblasts (Pemberton et al., 2014). We found 20 proteins whose genes are potential targets for CBX proteins, including *CDKN2A* (Figures 1E and 1F). In fact, knockdown of *CBX7* led to more than 2-fold upregulation of the mRNA levels of the majority of the 20 genes, as shown by qPCR (Figures 2A and S1C), while overexpression of murine Cbx7 resulted in gene silencing and transcriptional repression (Figures 2B and S1D). This was repeated using a different strain of human fibroblast, IMR-90 (Figure S1E). To further confirm that these genes are regulated by CBX7, we performed ChIP for endogenous CBX7 in BFs and analyzed the enrichment of CBX7 at the transcription start site (TSS) of the 20 genes. Our data show enrichment for CBX7 at the TSS of the analyzed genes, including *INK4A* (encoding $p16^{INK4A}$), but not the negative controls *ARF* (encoding $p14^{ARF}$) or *ACTB* (encoding β-actin) (Figure 2C).

ITGB3 Is Regulated by PRC1

Among the potential inducers of senescence regulated by CBX7, we focused on *ITGB3* (which encodes for the integrin β3 subunit) because: (1) it is a component of the two most representative pathways upon *CBX7* knockdown in the SILAC; (2) we found CBX7 at the TSS of *ITGB3* in the ChIP-seq dataset; and (3) it is the gene that is most upregulated upon *CBX7* knockdown. Interestingly, two additional PRC1 proteins, CBX8 and RING1B, were also found at the TSS of the *ITGB3* locus (Figure 2D), further supporting the regulation of *ITGB3* by PRC1. To confirm whether the latter changes at the mRNA level correlate with protein levels, we checked the levels of the integrin heterodimer αvβ3 by immunofluorescence (IF) (Figure 2E) and the β3 subunit by immunoblot upon *CBX7* knockdown (Figure S1F) or Cbx7 ectopic expression (Figure 2F). Importantly, we observed that shCBX7 increases the number of cells presenting αvβ3-stained FA complexes by IF (Figure 2E). The regulation of β3 protein levels by CBX7 was also confirmed in IMR-90 fibroblasts (Figure S1G). Altogether, these data show that the *ITGB3* locus is regulated by the Polycomb protein CBX7 in human primary fibroblasts.

ITGB3 Induction of Senescence Is Dependent on the $p53/p21^{CIP}$ Pathway

The aforementioned findings suggest that ITGB3 could be a regulator of cellular senescence. Indeed, expression of a retroviral vector encoding ITGB3 in BFs reduces their proliferation rate, quantified by measuring the percentage of cells incorporating bromodeoxyuridine (BrdU) (Figure 3A). A retroviral construct expressing the oncogene $H\text{-}Ras^{G12V}$ (RAS) was used as a positive control (Serrano et al., 1997). Concomitant with the growth arrest, we observed an increase in the protein levels of the cell-cycle inhibitors $p21^{CIP}$ and p53 by IF (Figure 3B) and *CDKN2B* mRNA levels by qPCR, with no changes observed in *CDKN2A* or *CDKN1B* (Figure 3C). Consistent with the activation of senescence, ITGB3 expression led to an increase in the number of cells staining positive for senescence-associated β-galactosidase (SA-β-Gal) activity (Figure 3D), an accumulation of reactive oxygen species (ROS) (Figure 3E), and a mild increase in the mRNA levels of different SASPs (Figure 3F). However, we failed to observe a DNA-damage response upon ITGB3 expression (Figure S2A). Importantly, ITGB3 expression also induced senescence in IMR-90 fibroblasts, indicating that this response is not strain specific (Figures S2B–S2D). We confirmed that the activation of senescence by ITGB3 expression in BFs is dependent on the p53 pathway. Using a previously characterized shRNA targeting *TP53* (shp53) (Acosta et al., 2008), we impaired not only the proliferation arrest induced by *ITGB3* (Figure 3G, left panel) but also the increase in SA-β-Gal activity (Figure 3G, right panel). The use of a short interfering RNA (siRNA) targeting *TP53* (sip53) also impaired the growth arrest induced by ITGB3 expression (Figure S2E). Thus, ITGB3 ectopic expression induces senescence in human primary fibroblasts, which is dependent on the $p53/p21^{CIP}$ pathway.

ITGB3 mRNA Levels Are Dynamically Regulated during Senescence

We next decided to determine whether *ITGB3* was endogenously regulated during senescence. As OIS is a potent tumor suppressor mechanism both in vitro and in vivo (Muñoz-Espín and Serrano, 2014), we used BFs stably expressing the oncogene RAS. Staining for αvβ3 by IF shows a substantial increase in its expression levels upon RAS induction. In addition, αvβ3 co-localizes with F-actin, indicating that it is part of FA complexes during RAS activation (Figure 4A). We confirmed that β3 protein and transcript levels are endogenously upregulated upon RAS expression compared to the vector control in an additional strain of human and also in mouse fibroblasts (Figure S3A). We also observed the upregulation of β3 in BFs during DNA-damage-induced senescence (DDIS) induced by etoposide treatment (Figures 4B and S3B). Moreover, treatment of two different cancer cell lines, MCF7 (breast) and SK-HEP-1 (liver) (Bollard et al., 2016), with a CDK4/6 inhibitor (Palbociclib or Palbo), mimicking TIS, also triggered endogenous upregulation of β3 (Figures 4C, S3C, and S3D). Intriguingly, the upregulation of *ITGB3* mRNA levels in SK-HEP-1 cells could only be observed after 10 days of treatment with Palbo, concomitant with the establishment of senescence. To determine the temporal changes of *ITGB3* during senescence, we took advantage of IMR-90 fibroblasts expressing an endoplasmic reticulum (ER):RAS fusion protein (ER:RAS). Upon treatment with 4-hydroxytamoxifen (4OHT), senescence is progressively established, displaying an initial mitotic arrest followed by full senescence induction after 4–6 days treatment. Though the mRNA levels of *ITGB3* at early timepoints of the induction of senescence were downregulated, we did observe a consistent upregulation of *ITGB3* during the establishment of senescence (Figure S3E). This pattern highly resembles the recently described temporal changes induced by NOTCH1 during OIS (Hoare et al., 2016).

As integrins play a predominant role in cellular signaling and adhesion, we next investigated whether other integrin beta subunits were deregulated during OIS. To avoid the confounding effects of integrin changes during the initial phases of the establishment of senescence, we decided to measure the mRNA expression levels of *ITGB1-8* using BFs stably expressing RAS. Our data show a noticeable deregulation of integrin beta subunits during OIS, with upregulation of *ITGB1*, *3*, *4*, and *6* during

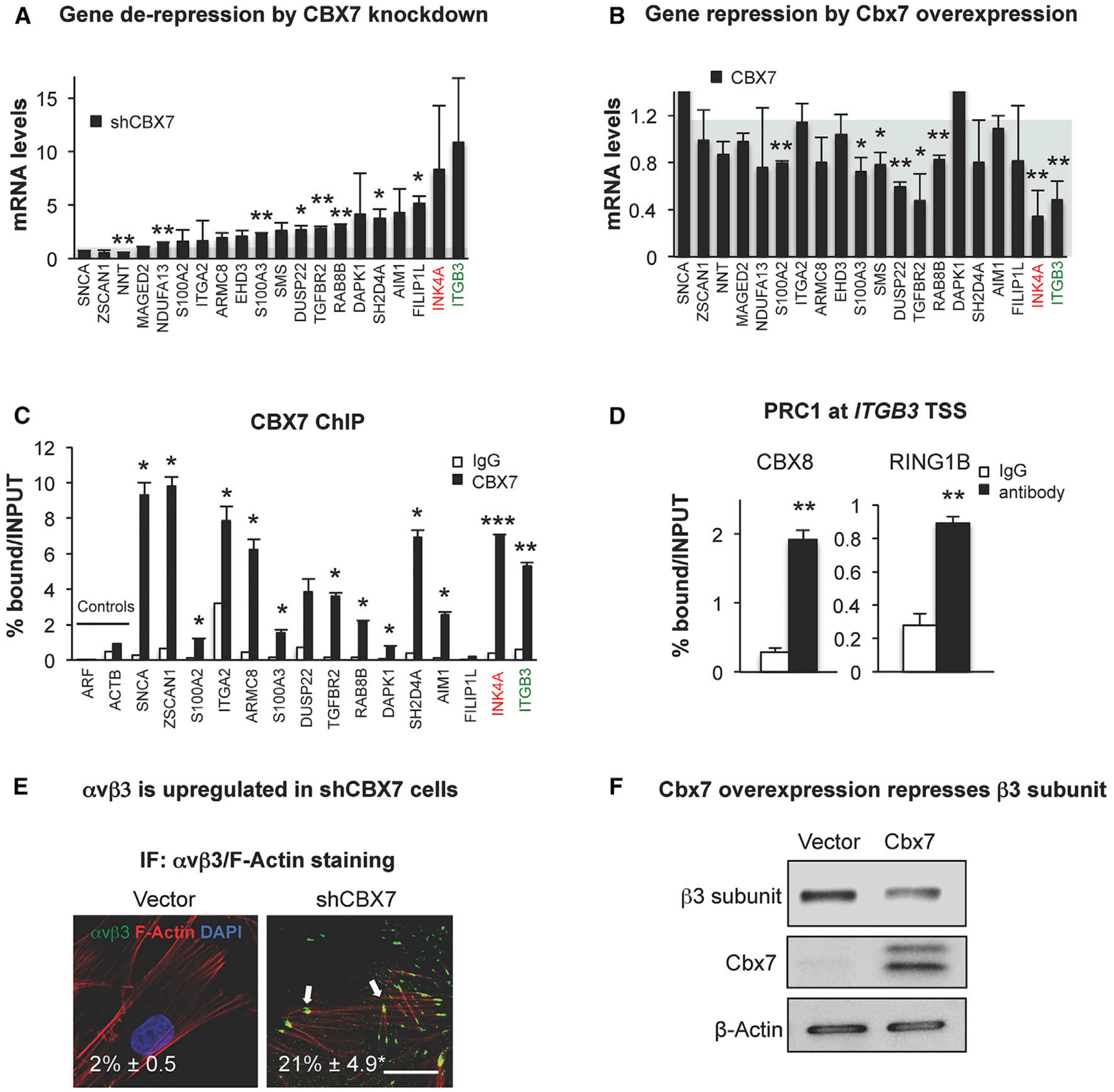

Figure 2. The Genes Encoding the Proteins Found in the SILAC Screen Are Regulated by CBX7

(A) qPCR analyses show the relative mRNA levels of the selected genes upon shCBX7. *INK4A* is highlighted in red as a known CBX7-regulated gene, and *ITGB3* is highlighted in green as a potentially new gene regulated by CBX7. Data are normalized to the control, shown as a gray shade, and represent the mean ± SD of two independent experiments.

(B) Overexpression of Cbx7 reduces the expression of its target genes. Relative mRNA levels are shown by qPCR. Data are normalized to the control, shown as a gray shade, and represent the mean ± SD of two independent experiments.

(C) ChIP for endogenous CBX7 (black bars) shows enrichment at the transcription start site (TSS) of its target genes, in comparison with immunoglobulin G (IgG) control (white bars). There is no CBX7 enrichment at the TSS of non-PRC1 target genes (Controls): *ARF* (encoding $p14^{ARF}$) and *ACTB* (β-actin). Data represent the mean ± SD of a representative experiment.

(D) ChIP for other PRC1 proteins (CBX8 and RING1B) show enrichment at the TSS of *ITGB3*, in comparison with IgG (white bars). A representative experiment is shown.

(E) Representative images showing integrin αvβ3 (green) and F-actin (red) staining in fibroblasts expressing empty vector or shCBX7. The formation of αvβ3-stained FA complexes can be observed only in cells harboring shCBX7 (white arrows). The quantification indicates the percentage of cells positive for αvβ3 staining ± SD (three to five independent experiments). Scale bar, 20 μm.

(F) A representative blot for BFs overexpressing Cbx7 shows reduced levels of endogenous β3 subunit and mouse Cbx7 overexpression levels. β-actin is used as loading control.

*$p < 0.05$; **$p < 0.01$; ***$p < 0.001$.

RAS expression, *ITGB3* being the subunit most upregulated (Figure 4D). Therefore, we show that *ITGB3* mRNA levels increase concomitantly with the establishment of senescence.

CBX7 Regulates *ITGB3* Locus during OIS

Since *ITGB3* locus is regulated by CBX7 (Figure 2), we reasoned that the endogenous upregulation of *ITGB3* mRNA upon RAS

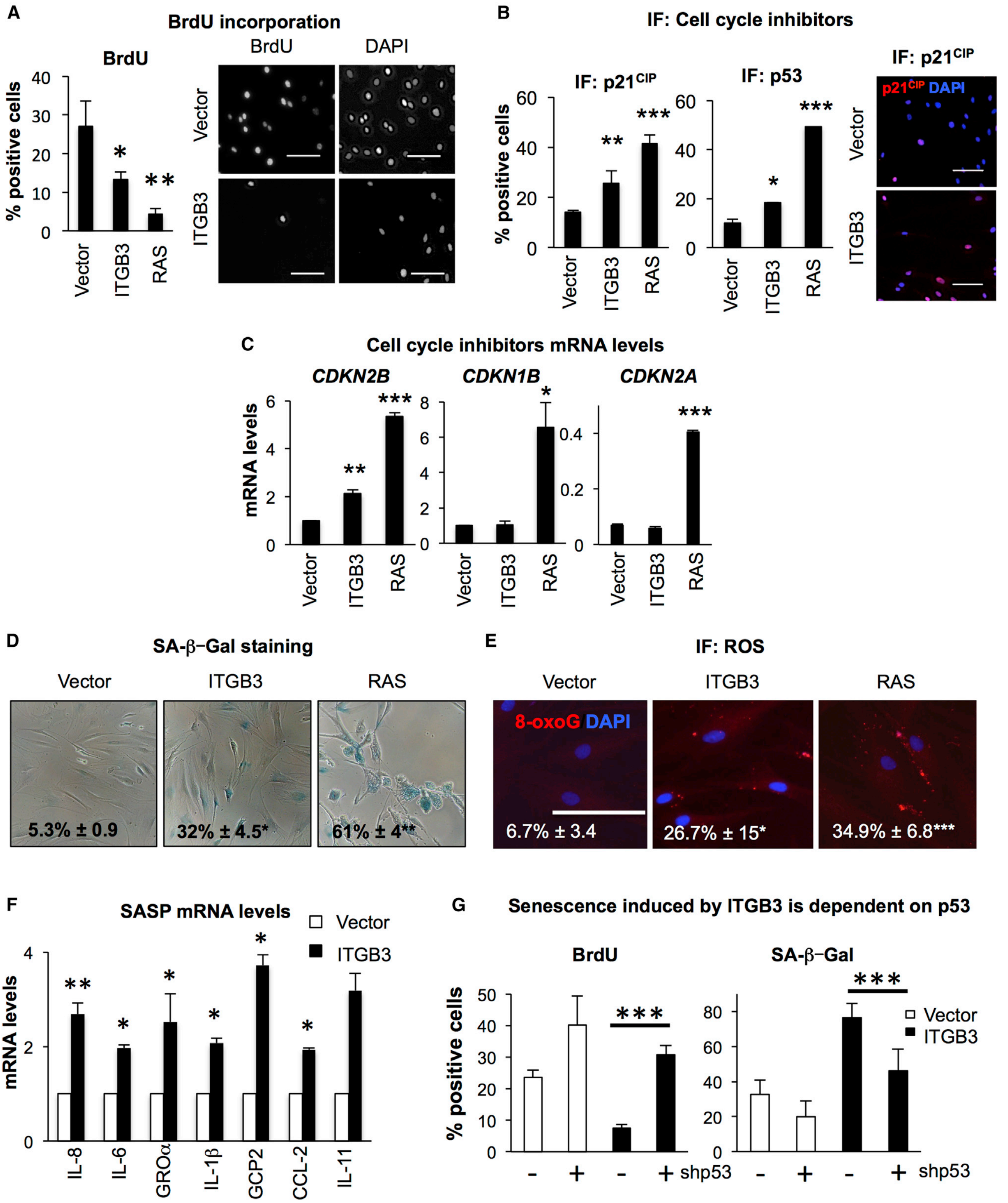

Figure 3. ITGB3 Ectopic Expression Induces Senescence via $p21^{CIP}$/p53 Pathway

(A–F) Overexpression of a retroviral construct encoding ITGB3 in BFs induces senescence. H-Ras^{G12V} (RAS) is used as a positive control for inducing senescence. (A) We show a reduction in proliferation in BFs expressing ITGB3 by measuring the percentage of cells incorporating BrdU (left panel: quantification levels; right panel: representative pictures). Proliferation was assessed 4–5 days after plating. BFs expressing ITGB3 show (B) an increase in $p21^{CIP}$ and p53 protein

(legend continued on next page)

expression could be due to epigenetic regulation by CBX7. To test this hypothesis, we performed ChIP for endogenous CBX7 on the *ITGB3* locus in BFs transduced with vector or RAS. Our data show a reduced binding of CBX7 to the *ITGB3* TSS during OIS (Figure 4E), suggesting that the endogenous upregulation of *ITGB3* during OIS is due to the transcriptional deregulation of the locus by the loss of CBX7 binding. As expected, we did not observe changes in CBX7 binding in vector or RAS BFs in a control-coding region (Figure 4E).

β3 Regulates Senescence Independently of Its Binding Activity

To test whether β3 has a functional role during senescence, we manipulated *ITGB3* mRNA levels during OIS and TIS, once senescence was fully established. The transduction of BFs expressing RAS with an shRNA targeting *ITGB3* (shITGB3) or transfection with two different siRNAs (siITGB3) impaired the proliferation arrest induced by RAS (Figures 4F, S3F, left panel, and S3G) and partially reverted the increase in the cell-cycle inhibitor $p21^{CIP}$ induced upon OIS, as shown by IF (Figure S3F, right panel). Furthermore, ablation of *ITGB3* mRNA by siRNA also overcame the proliferation arrest induced by Palbo treatment in MCF7 cells (Figure 4G). We next treated BFs stably expressing RAS with the αvβ3/αvβ5 antagonist cilengitide, as we reasoned that inhibiting αvβ3 could be a therapeutic treatment to overcome senescence. Surprisingly, treatment of BFs expressing RAS with cilengitide could not reverse the proliferation arrest (Figure 4H) or the upregulation of $p21^{CIP}$ or $p16^{INK4A}$ protein levels (Figure S4A). This suggested that β3 induces senescence independently of its ligand-binding activity, which was further confirmed by the ectopic expression of a mutant β3, defective for the ligand-binding domain ($ITGB3^{D119A}$) (Loftus et al., 1990) that also induced senescence (Figures S4B–S4E). Excitingly, although cilengitide treatment during OIS could not reverse the proliferation arrest induced by RAS or the upregulation of $p16^{INK4A}$ o $p21^{CIP}$ protein levels, we did observe a significant reduction of the SASP, as shown by measuring the levels of IL-8 and IL-6 secreted to the supernatant by immunoblot (Figure 4I). Therefore, αvβ3 inhibition is able to uncouple the SASP release from the proliferation arrest in OIS.

β3 Regulates Cellular Senescence in a Cell-Autonomous Fashion by Activating the TGF-β Pathway

To determine the pathway by which β3 induces senescence in BFs, we next used a panel of small molecule inhibitors. We assessed proliferation levels by quantifying BrdU incorporation and $p21^{CIP}$ levels by IF (schematic representation of the drug screen and timings are shown in Figures 5A and 5B). Out of all the chemical compounds used, we found that the inhibitors targeting TGF-β-receptor 1 (TGFBR1 or ALK5), αvβ3/αvβ5 integrin (cilengitide), Rho-associated kinases 1/2 (ROCK1/2), and integrin-linked kinase (ILK) were capable of reversing not only the proliferation arrest induced by β3 expression (Figure 5C) but also the upregulation of $p21^{CIP}$ (Figure S5A). Oddly, cilengitide did affect proliferation in this setting, which could be explained by compensation of αvβ5 upon ITGB3 overexpression (*ITGB5* is downregulated during OIS [Figure 4D] but not upon ITGB3 expression [data not shown]). As previous reports have shown that TGF-β regulates senescence (Acosta et al., 2013; Muñoz-Espín et al., 2013) and integrins are known to activate TGF-β (Asano et al., 2005; Margadant and Sonnenberg, 2010), we thought it would be interesting to determine whether β3 regulates senescence via activation of the TGF-β pathway. To further investigate this possibility, we analyzed the effect of a scramble siRNA (Scr) and two independent siRNAs targeting TGF-β-receptor 2 (siTR2_2 and siTR2_7) on the proliferation of BFs expressing vector or ITGB3. Our data demonstrate that both siRNAs against *TGFBR2* overcome senescence induced by the overexpression of ITGB3, as shown by measuring the relative cell number and $p21^{CIP}$ levels by IF (Figures 5D and S5B). Interestingly, we could not reproduce these results using pan-specific neutralizing anti-TGF-β1–3 antibodies (data not shown), suggesting that ITGB3 regulates TGF-β, at least partially, in a cell-autonomous fashion.

The TGF-β superfamily comprises a number of molecular players, including receptors (TGFBR1 and -2), ligands (TGF-β1, -2, and -3), effectors (SMAD proteins), and ECM-binding proteins (LTBPs or latent TGF binding proteins). Upon binding of TGF-β ligands with TGFBRs, the pathway becomes active, and specific SMAD proteins translocate to the nucleus to control gene expression (Schmierer and Hill, 2007). We reasoned that, if β3 is inducing senescence by activating the TGF-β pathway, we should find differences in the expression levels of different members of this pathway. Indeed, qPCR analyses of a range of regulators implicated in the TGF-β pathway are upregulated in BFs expressing ITGB3 (Figure 5E). To further confirm that the pathway is active upon ITGB3 expression, we next measured the translocation of SMAD2/3 to the nucleus by IF. Indeed, BFs expressing ITGB3 showed a higher percentage of cells staining positive for nuclear SMAD2/3 (Figure 5F). Altogether, our data show that *ITGB3* regulates senescence via TGF-β activation.

Non-Cell-Autonomous Effect of β3 on Human Primary Fibroblasts

Integrins can activate TGF-β embedded in the ECM by increasing the expression of matrix-degrading enzymes (matrix metalloproteinases; MMPs) and the proteolytic release of TGF-β to the media. Therefore, we determined the expression

levels by IF 4–5 days after plating (left panel: percentage of cells stained positive for $p21^{CIP}$ and p53; right panel: representative pictures for $p21^{CIP}$ staining) and (C) an increase in *CDKN2B* (encoding $p15^{INK4B}$) mRNA levels by qPCR. No changes were observed in *CDKN1B* (encoding $p27^{KIP1}$) or *CDKN2A* ($p16^{INK4A}$). (D) Expression of ITGB3 also induced an increase in senescence-associated β-galactosidase activity (SA-β-Gal). Data represent the percentage of cells staining positive for SA-β-Gal ± SD. Staining was performed 7–10 days after plating; (E) an increase in the levels of ROS, measured by 8-oxoG staining, and (F) a mild increase of the mRNA levels of different SASP by qPCR. Cells were subjected to analysis (either by IF or qPCR) 4–5 days after plating.

(G) An shRNA against p53 (shp53) prevents the activation of senescence induced by ITGB3 ectopic expression, as shown by the reversion in the percentage of BFs incorporating BrdU induced by ITGB3 (left graph) and the decrease in SA-β-Gal activity (right graph). BrdU was added 24 hr prior to fixing the cells for IF. Data represent the mean ± SD of more than two independent experiments. Scale bars, 100 μm. *$p < 0.05$; **$p < 0.01$; ***$p < 0.001$.

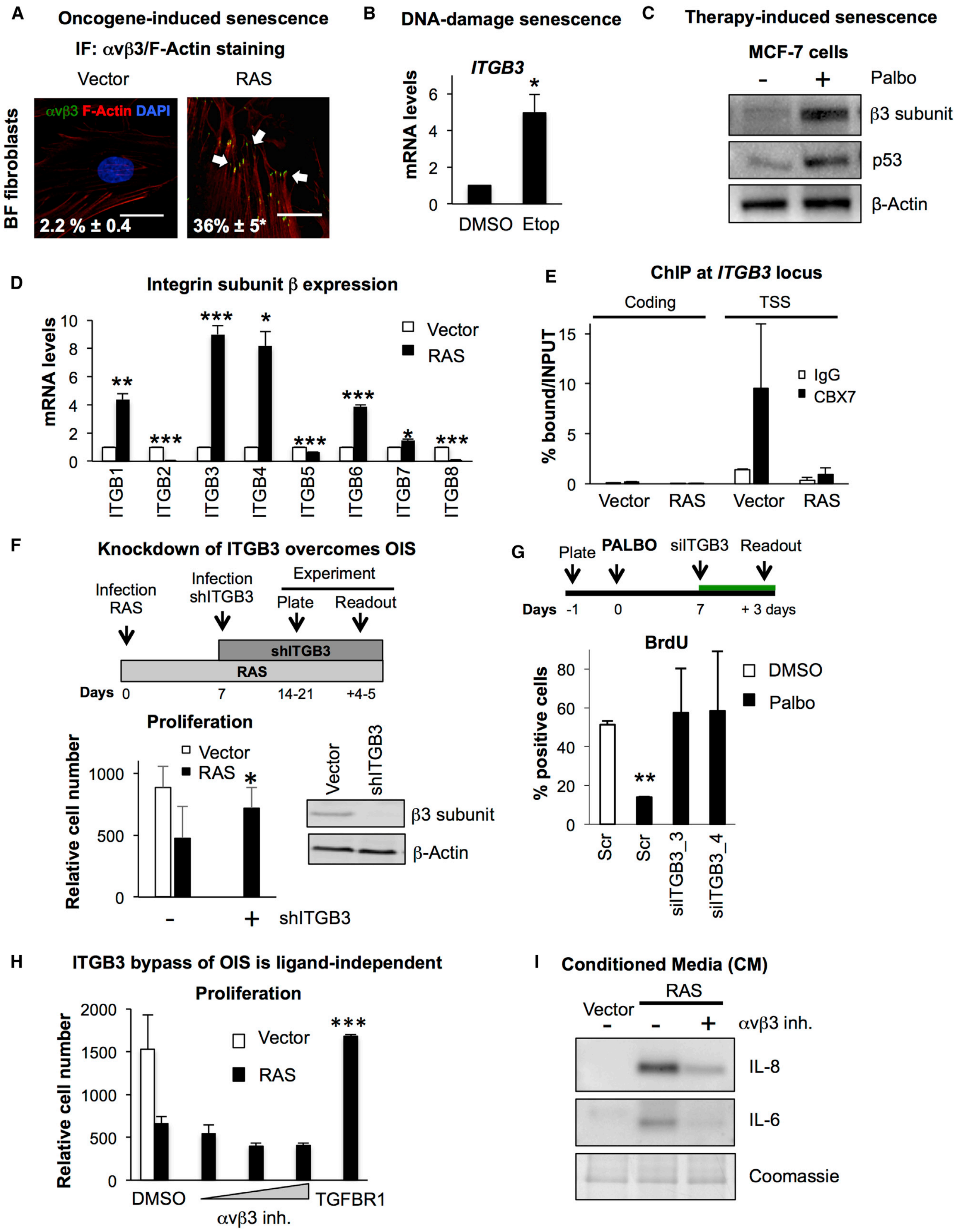

(legend on next page)

levels of different MMPs in BFs expressing ITGB3 and found upregulation of the mRNA levels of *MMP1* and *MMP9*, indicating that ITGB3 expression can directly activate the TGF-β pathway (Figure 5G). We next tried to determine whether TGF-β was being released to the supernatant in BFs expressing ITGB3 and had a non-cell-autonomous role on surrounding cells. While we could not detect TGF-β in the supernatant (data not shown), we did find that the conditioned media (CM) from cells expressing ITGB3 had an effect on normal BFs by inducing the stabilization of p53 protein (Figure 5H), the nuclear translocation of SMAD2/3 (Figure 5I), and a reduced proliferation rate (Figure S5C) in normal BFs. Furthermore, treatment with a pan-specific neutralizing anti-TGF-β1-3 antibody (Figure 5H) or an inhibitor for TGFBR1 (Figure 5I) abrogated the effect of the CM from ITGB3 cells, suggesting that the non-cell-autonomous effect of ITGB3 cells is dependent on TGF-β.

β3 Subunit Expression Increases during Replicative Senescence

Activation of senescence has been described in a variety of physiological and pathological conditions, including aging. In fact, the activation of cellular senescence is considered one of the hallmarks of aging (López-Otín et al., 2013). In order to determine whether the β3 subunit is upregulated during aging, we used a retroviral construct encoding the dominant-negative allele of the telomeric repeat binding factor 2 ($TRF2^{\Delta B \Delta M}$). Expression of $TRF2^{\Delta B \Delta M}$ in primary fibroblasts rapidly mimics the process of replicative senescence and aging (Karlseder et al., 1999). Similar to our previous results, where β3 is upregulated in senescence, $TRF2^{\Delta B \Delta M}$-expressing BFs presented an increase in β3 subunit (Figure 6A). This was further confirmed in murine hepatic stellate cells (mHSCs) extracted from an adult mouse harboring a doxycycline (Dox)-inducible construct to express shp53 (Lujambio et al., 2013). Upon Dox withdrawal, senescence is induced by re-expression of p53 and mHSCs showed an increase in a number of markers of senescence (Figure S6A), concomitant with the accumulation of *Itgb3* (Figure 6B).

ITGB3 mRNA Levels Are Dynamically Upregulated during Aging in Mice

To determine whether *Itgb3* expression is changed during aging in vivo, we extracted RNA from liver tissue of C57BL/6J female mice aged 4, 19, and 25 months. Livers from mice aged 19 months presented a dynamic increase at the mRNA levels of *Cdkn2a* and *Itgb3*, but the highest increase was observed in 25-month-old mice, where additional markers of senescence were observed (Figures 6C and S6B). This is in agreement with our in vitro data that show a concomitant upregulation of *ITGB3* with *CDKN2A* mRNA levels (Figure S3E). Upregulation of *Itgb3* mRNA and other markers of senescence were also observed in kidney (Figures 6D and S6B) and, to a lesser extent, in the intestine in 25-month-old mice (Figures S6B and S6C).

β3 Subunit and TGF-β Components Are Highly Expressed in Fibroblasts Derived from Old Human Donors

Next, we took advantage of primary skin fibroblasts derived from young (~10 years old) and old (~80 years old) human donors and tested whether a correlation between senescence and β3 subunit existed during aging (Figure S6D). In order to confirm that fibroblasts derived from old donors behaved as senescent cells, we analyzed different senescence markers. We could, indeed, observe that fibroblasts derived from old donors presented a reduced proliferative capacity measured by relative cell number (Figure S6E) and had a significant increase in $p16^{INK4A}$ and $p21^{CIP}$ protein levels, as measured by IF, compared to young donor cells (Figure S6F). We then analyzed the expression levels of β3 subunit between fibroblasts from young and old donors and observed an increase in β3 by immunoblotting (Figure 6E), an increase in the percentage of αvβ3 staining in FA complexes by IF (Figures 6F and S6G), and an increase in *ITGB3* at the RNA

Figure 4. ITGB3 Regulates Senescence Independently of Its Binding Activity

(A) Endogenous αvβ3 expression increases during OIS upon RAS expression in BFs. Representative pictures for αvβ3 (green) and F-actin (red) staining by IF in vector and RAS cells are shown. αvβ3-stained FA complexes are indicated with white arrows. Data represent the percentage of cells positive for αvβ3 staining. Scale bar, 20 μm.

(B) *ITGB3* is endogenously upregulated during DNA-damage-induced senescence (DDIS). BFs were treated with 100 μM etoposide (Etop) for 2 days and replaced with fresh media for 5 days.

(C) MCF7 breast cancer cells were treated with 200 nM palbociclib (Palbo) for 7 days, after which cells were lysed for immunobloting. An increase in β3 subunit and p53 can be observed after Palbo treatment.

(D) mRNA analyses for *ITGB* subunits 1–8 during RAS-induced senescence in BFs.

(E) CBX7 binding to *ITGB3* TSS is reduced during OIS. ChIP for CBX7 enrichment (black bars) versus IgG control (white bars) at an *ITGB3* TSS and a coding region in BFs expressing vector or RAS.

(F) Schematic representation of the timings used to determine the role for ITGB3 overcoming OIS (top panel). Lower left panel: relative cell number in RAS-expressing BFs transduced with a vector or an shRNA targeting *ITGB3* (shITGB3). Lower right panel: representative immunoblot showing β3 subunit knockdown efficiency.

(G) Top panel shows the experimental planning. Senescence was induced by Palbo treatment, after which siITGB3 was transfected (green bar). Two independent siRNAs targeting ITGB3 (siITGB3) overcame the senescence arrest induced by treating MCF7 cells with Palbo for 7 days. BrdU was added 24 hr before the end of the experiment.

(H) Cells expressing RAS were treated with DMSO or αvβ3 inhibitor (cilengitide) for 48 hr, and the relative cell number was calculated. Increasing concentrations of cilengitide (10, 25, and 50 nM) show no reversion of the proliferation arrest induced by RAS. An inhibitor for TGF-β-receptor 1 (TGFBR1, 4 μM) was used as positive control.

(I) Immunoblot for the conditioned media (CM) from cells expressing either vector or RAS treated with or without 50 nM of αvβ3 inhibitor (cilengitide) for 48 hr, followed by a 72-hr incubation in fresh media. Coomassie staining is shown as loading control.

*$p < 0.05$; **$p < 0.01$; ***$p < 0.001$.

OPEN
ACCESS
CellPress

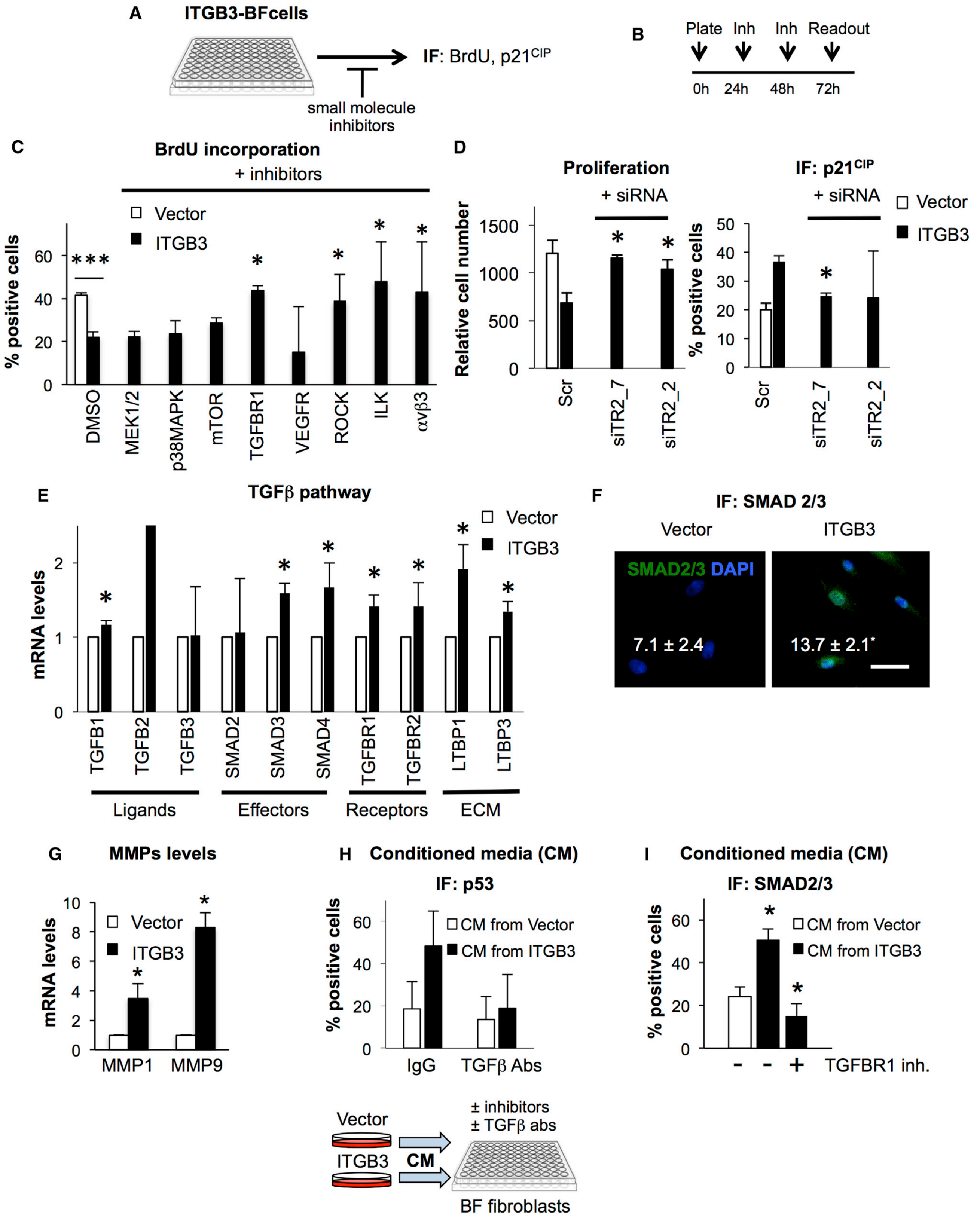

(legend on next page)

level (Figure S6H) in fibroblasts from old compared to young donors. We have previously shown that activation of senescence by β3 is dependent on the TGF-β pathway. To determine whether the same activation pathway applies to fibroblasts derived from human donors, we analyzed the expression levels of different regulators of the TGF-β pathway. Interestingly, we could also observe an increase in the mRNA levels of different regulators of the TGF-β pathway, including TGF-β receptors 1 and 2 and SMAD3 and 4 (Figure 6G). Altogether, these data show the existence of a positive correlation between senescence and the expression levels of β3 subunit and different regulators of the TGF-β pathway in aging.

ITGB3 Plays a Role in Aging in Fibroblasts Derived from Old Human Donors

We next decided to manipulate the expression levels of ITGB3 in fibroblasts derived from young and old donors to identify whether *ITGB3* plays a role in this model. As expected, ectopic expression of either ITGB3 or RAS in fibroblasts derived from two different young donors induced senescence-like growth arrest, as observed by a reduction in the percentage of cells incorporating BrdU (Figure 7A) and an upregulation of cells staining positive for $p21^{CIP}$ (Figure 7B). We next decided to determine whether reducing the endogenous levels of *ITGB3* mRNA in cells derived from old donors could attenuate aging. To this end, we chose the two fibroblasts from old donors that expressed the highest levels of β3, and we reduced *ITGB3* expression levels using RNAi. Transfection with two different siRNAs (siITGB3) and sip53, overcame the proliferation arrest characteristic of old fibroblasts (Figure 7C) and partially reverted the increase in the cell-cycle inhibitor $p21^{CIP}$ (Figure 7D). This was further confirmed using shITGB3 (Figure S7A). As our previous data show that ITGB3 induces senescence independently of its ligand-binding activity, we investigated whether this mechanism was conserved during aging. Treatment of old donor cells with cilengitide could not attenuate senescence, neither the proliferation arrest nor $p21^{CIP}$ upregulation (Figures 7E and 7F), suggesting that the role for ITGB3 in aging in human primary fibroblasts is independent of its ligand-binding activity. Altogether, these data suggest that ITGB3 is a regulator of aging in the human primary fibroblasts derived from old donors in this study.

DISCUSSION

Intercellular communication is an important feature to maintain tissue homeostasis, where the activation of cellular senescence plays a crucial role. In fact, previous reports have found ECM remodeling to regulate fibrosis by activating the senescence program (Jun and Lau, 2010; Krizhanovsky et al., 2008; Lujambio et al., 2013). Apart from inflammation and ECM remodeling, cells can communicate via the secretion of extracellular vesicles (Tkach and Théry, 2016), cell-cell contact (Hoare et al., 2016), or intercellular protein transfer (Biran et al., 2015). Here, we provide evidence that the integrin β3 subunit plays a role in senescence through activation of the TGF-β pathway.

A great deal of information exists regarding the biological function of integrins and their regulation of the microenvironment, but relatively little is known about the transcriptional regulation of integrins themselves. A recent report has found that MYC overexpression leads to a direct downregulation of *ITGB3*, inducing decreased motility and invasiveness (Liu et al., 2012). Our results further add PRC1 complex as a regulator of the *ITGB3* locus in normal fibroblasts and for CBX7 during OIS, where CBX7 binding to the *ITGB3* TSS is reduced. In fact, *ITGB3* has been previously identified as a Polycomb target by ChIP sequencing in other biological contexts, such as mouse embryonic stem cells (ESCs) (Morey et al., 2013) and human primary fibroblasts (Pemberton et al., 2014), suggesting that the *ITGB3* locus is epigenetically regulated in several biological contexts.

Integrin signaling regulates diverse functions in cancer, angiogenesis, stemness, and drug resistance (Desgrosellier and Cheresh, 2010). In addition, integrins also regulate fibrosis and wound healing (Margadant and Sonnenberg, 2010). Our findings establish the β3 subunit as a regulator for cellular senescence. We show that β3 subunit expression accelerates the onset of senescence in human primary fibroblasts, which is dependent on the

Figure 5. ITGB3 Induces Senescence by Activating the TGF-β Pathway in a Cell-Autonomous and Non-Cell-Autonomous Fashion

(A and B) Shown here are (A) a schematic representation and (B) timings of a miniscreen for small molecule inhibitors (Inh) used to determine the pathway activating senescence induced by β3.

(C) BFs expressing vector or ITGB3 were treated for 48 hr with a variety of drugs inhibiting different signaling pathways. Drugs were renewed every 24 hr, and BrdU was added 16 hr prior to fixing the cells. The percentage of BrdU-positive cells with or without the inhibitors is shown. The graph indicates the inhibitor's targets: 40 μM PD98059 (targeting MEK1/2), 20 μM SB202190 (p38MAPK), 100 nM TORIN2 (mammalian target of rapamycin; mTOR), 4 μM TGF-β-R1 (TGFBR1), 8 μM Vegfr-2/Flt3/C-Kit (VEGFR), 150 nM GSK429286A (ROCK1/2, Rho-associated kinase), 50 nM Cpd22 (ILK, integrin-linked kinase), and 50 nM cilengitide (αvβ3). Except when indicated, asterisks represent the statistical differences for cells expressing ITGB3 treated with DMSO or the different small molecule inhibitors.

(D) Knockdown of TGFBR2 overcomes senescence induced by β3. We measured the proliferation (left graph) and $p21^{CIP}$ levels (right graph) in BF fibroblasts transiently transfected with a scramble (Scr) or two independent siRNAs against *TGFBR2* (siTR2_2 and 7) for 4–5 days.

(E) qPCR analyses of different regulators of the TGF-β pathway: ligands (TGFB1, -2, and -3), effectors (SMAD2, -3, and -4), receptors (TGFBR1 or ALK5, TGFBR2), and ECM proteins (LTBP1, -3, or latent TGF binding proteins).

(F) Representative IF pictures for SMAD2/3 staining and quantification of the percentage of cells positive for nuclear SMAD2/3. Scale bar, 50 μm.

(G) *MMP1–MMP9* mRNA levels upon ITGB3 expression.

(H and I) Normal BFs were treated with conditioned media (CM) from BFs expressing vector or ITGB3. (H) Pan-specific neutralizing anti-TGF-β1-3 antibodies inhibit the stabilization of p53 induced by the CM taken from ITGB3 cells. A species matching IgG was used as negative control. Bottom panel: a diagram showing the experimental planning. CM was collected after 7 days and transferred to normal BFs with or without different treatments. (I) A TGFBR1 inhibitor (4 μM) blocks the nuclear translocation of SMAD2/3 induced by the CM taken from ITGB3 cells.

Data represent mean ± SD of 2–4 independent experiments. $^*p < 0.05$; $^{***}p < 0.001$.

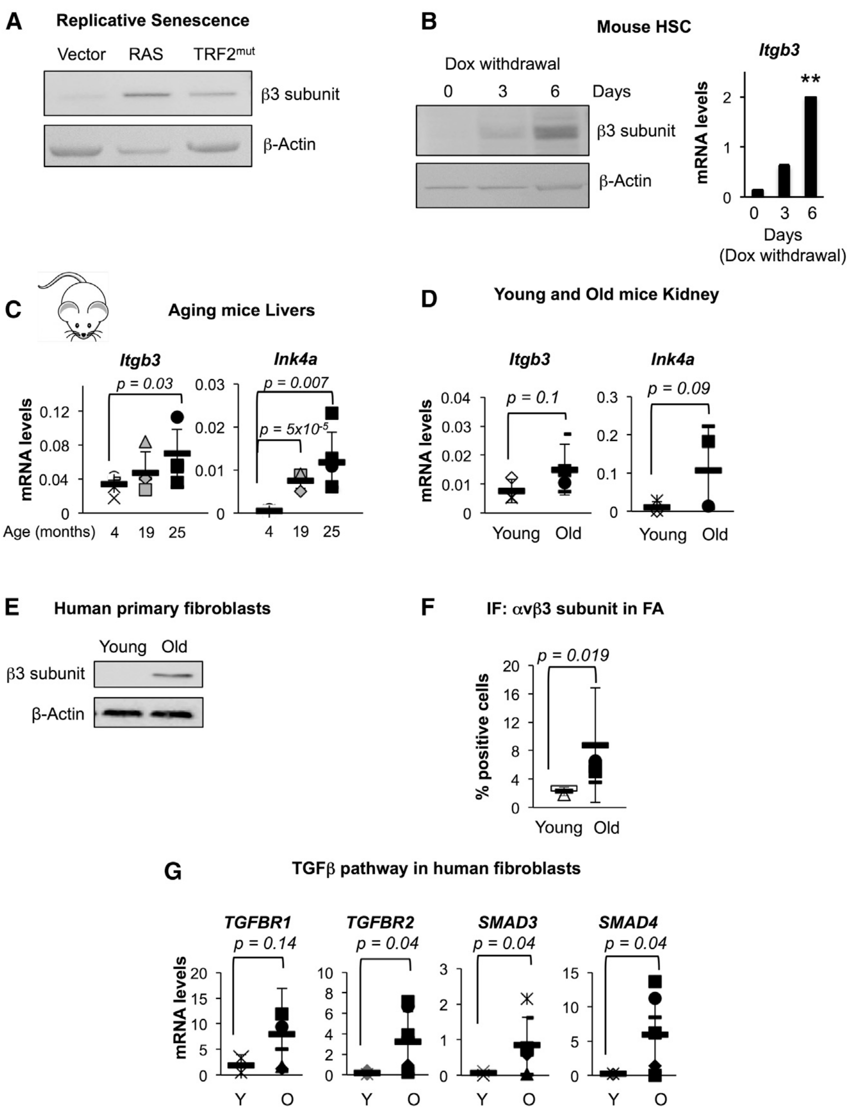

Figure 6. β3 Subunit Is Upregulated during Replicative Senescence and Aging in Human and Mouse

(A) Immunoblot for β3 subunit in BFs expressing empty vector, RAS or the dominant-negative telomeric repeat binding factor 2 ($TRF2^{\Delta B \Delta M}$), mimicking replicative senescence. β-actin is used as loading control.

(B) Immunoblot for β3 subunit (left panel) and qPCR analysis for *Itgb3* mRNA levels (right panel) in mouse hepatic stellate cells (mHSCs) upon different days of doxycycline (Dox) withdrawal.

(C) mRNA levels are shown for *Ink4a* and *Itgb3* in livers taken from C57BL/6J female mice aged 4, 19, and 25 months old.

(D) Kidneys from young (4 months) and old (25 months) C57BL/6J mice were subjected to qPCR to determine *Ink4a* and *Itgb3* mRNA expression levels.

(E) Representative immunoblot for β3 subunit in human primary fibroblasts derived from young and old donors. We observed similar results with other young and old samples. β-actin is the loading control.

(F) Quantification of the percentage of cells stained positive for αvβ3 in FA complexes by IF in young and old human fibroblasts. Data represent the mean ± SD of fibroblasts derived from young and old donors.

(G) qPCR analyses for TGF-β receptors 1 and 2 and SMAD3 and 4 in human fibroblasts from young (Y) and old (O) donors.

In (C) and (D), data represent the mean ± SD of 4–5 mice per condition. In (F) and (G), data represent the mean ± SD from fibroblasts from 2–4 young and 6–7 old donors.

activation of the $p21^{CIP}$/p53 pathways. This is complementary with a previous study, which shows activation of fibroblast senescence by the ECM protein CCN1 that binds to α6β1, activating ROS production (Jun and Lau, 2010). Our results also show a robust expression of β3 upon senescence activation induced by a variety of stimuli, while interference with its expression levels disrupts the senescence phenotype. In contrast, it was found that αvβ3 expression escapes OIS in glioblastoma by activating PAK4 (Franovic et al., 2015) and that mice expressing β1-deficient tumors show reduced tumor burden and activation of senescence (Kren et al., 2007). Furthermore, mice lacking β3 accelerate wound-healing closure, which could be by restricting the induction of senescence. However, in contrast with our findings, the authors observed an increase in TGF-β signaling in β3 null mice (Reynolds et al., 2005). All these seemingly paradoxical behaviors of integrin-signaling activity could be due to differences in the cellular and environmental contexts during senescence activation, as it has been previously described for $H\text{-}Ras^{G12V}$ (Serrano et al., 1997) and the chemokine receptor CXCR2 (Acosta et al., 2008).

Cellular adhesion is a key feature of senescence. In agreement with our results, several reports have found differential expression of integrins during cellular senescence activation. Analysis of published datasets show that the "cellular adhesion" pathway and integrins are differentially expressed during senescence activation (Fridman and Tainsky, 2008; Storer et al., 2013). Likewise, a number of studies have found that TGF-β ligands are part of the SASP and play an important role in senescence through $p21^{CIP}$ regulation, in agreement with our data (Acosta et al., 2013; Hoare et al., 2016; Muñoz-Espín et al., 2013; Storer et al., 2013). The TGF-β superfamily controls numerous cellular and biological processes, such as development, regeneration, fibrosis, and cancer (Macias et al., 2015). Accumulating evidence indicates that a cross-talk between integrins and TGF-β exists, in particular to regulate fibrosis, wound healing, and cancer (Asano et al., 2005; Margadant and Sonnenberg, 2010). However, even if senescence is known to regulate all these biological processes, none of these studies have reported the existence of a cross-talk between integrins and TGF-β in senescence or aging. Our data show that β3 regulates senescence by activating TGF-β via cell-autonomous and

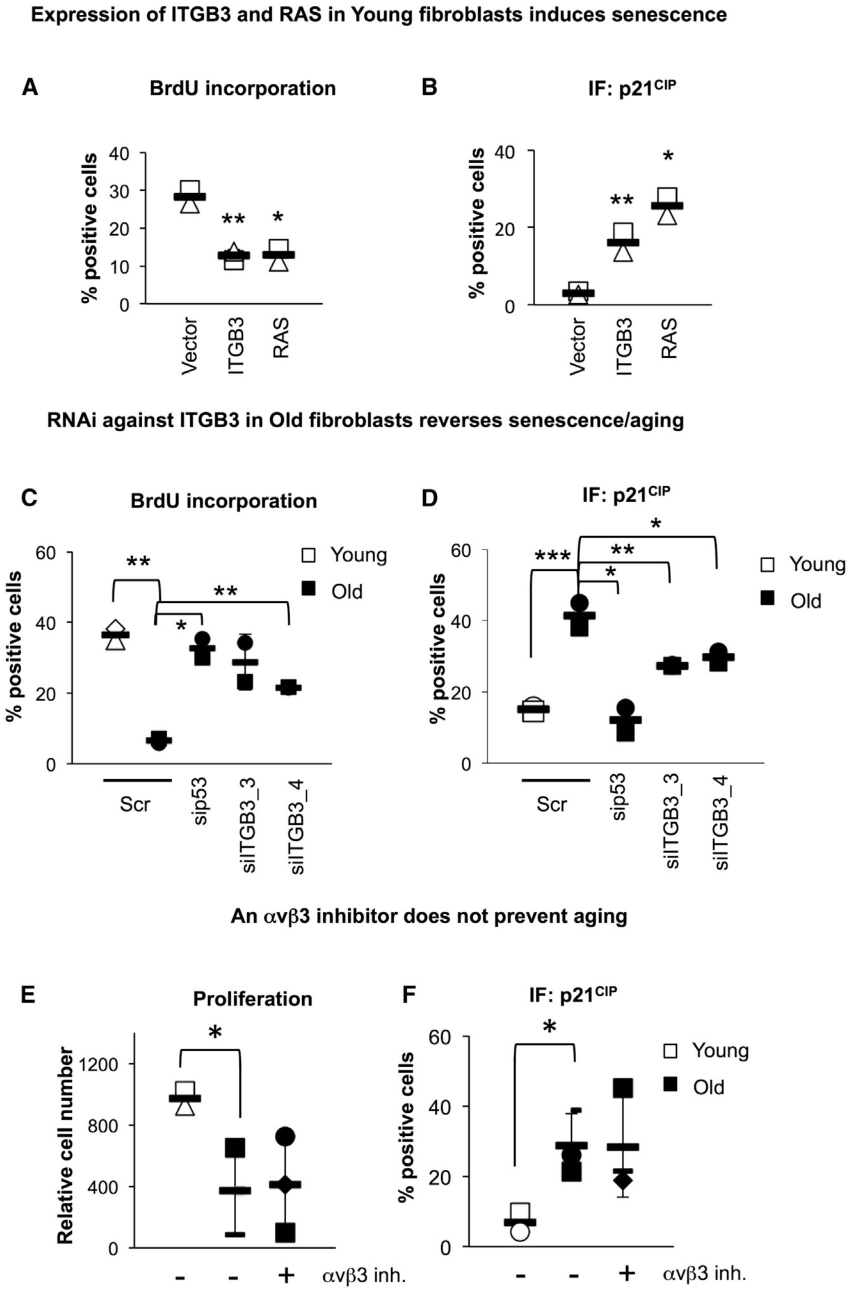

Figure 7. Changes in the Expression Levels of *ITGB3* Affect Aging and Senescence Cellular Features

(A and B) Ectopic expression of a construct encoding either RAS or ITGB3 induces a senescence-like arrest in human fibroblasts derived from two independent young donors. (A) Percentage of BrdU-positive cells and (B) $p21^{CIP}$ protein levels quantified by IF are shown.

(C and D) RNAi targeting *ITGB3* in fibroblasts derived from old donors averts cellular features of aging and senescence. Cells derived from two young donors were used as controls (white filling). Fibroblasts from old donors (black filling) were transiently transfected with a scramble (Scr) or two independent siRNAs against *ITGB3* (siITGB3_3 and 4) for 4–5 days. sip53 was used as a control. (C) The percentage of cells staining positive for BrdU and (D) $p21^{CIP}$ were quantified by IF.

(E and F) Treatment of cells derived from old donors with cilengitide does not affect senescence/aging. Cells from two independent old donors (black filling) were treated with 50 nM cilengitide αvβ3 inhibitor for 2 days, and (E) the percentage of BrdU- and (F) $p21^{CIP}$-positive cells was assessed. inh., inhibitor.

All data represent the mean ± SD from cells derived from two independent young (A and B) or old (C–F) individuals. $^{*}p < 0.05$; $^{**}p < 0.01$; $^{***}p < 0.001$.

non-cell-autonomous mechanisms. The use of small molecule inhibitors, RNAi technology, and the analysis of the expression levels of various members of the TGFβ pathway authenticate a role for TGF-β during senescence induced by β3 expression.

Different reports have found that there is cross-talk between integrins and chemokine receptors (Desgrosellier and Cheresh, 2010). Although we could not detect any changes in the mRNA expression levels of *CXCR2* in cells expressing ITGB3 (data not shown), it would be interesting to further investigate a potential connection in senescence.

Senescence regulates tissue-regenerative capacity and homeostasis. In fact, αvβ3 expression is increased in a number of cell types undergoing tissue remodeling (Asselin-Labat et al., 2007; Brooks et al., 1994). Furthermore, integrins can direct specific stemness-related reprogramming, providing an important role during development independent of their ligand-binding activity (Seguin et al., 2015). Interestingly, developmental senescence is activated to promote tissue remodeling and stem cell renewal (Muñoz-Espín and Serrano, 2014). Our data show that senescence induced by β3 presents a similar pattern to developmental senescence (activation of $p21^{CIP}$, TGF-β/SMAD, and no DNA damage) and that it is independent of ligand binding. It would be interesting to investigate whether integrins also play a role in this context.

Our data show an increase in the expression levels of *Itgb3* mRNA concomitant with an increase in different markers of senescence in tissue from old mice. Upregulation of β3 and senescence/aging markers, including TGF-β members, was further observed in fibroblasts from old human donors. This is in accordance with previous reports, which have found that $p16^{INK4A}$ levels correlate with chronological age in most tissues analyzed, both in mice (Baker et al., 2016; Krishnamurthy et al., 2004) and in humans (Ressler et al., 2006). Interestingly, knockdown of *ITGB3* mRNA partially reversed the aging phenotype of fibroblasts derived from old human donors. However, the αvβ3 antagonist, cilengitide, could not reverse aging, suggesting that the role for β3 in this cellular system is independent of its ligand-binding

activity. Our data show that cilengitide has a diverse effect on the SASP and on the senescence growth arrest. As senescent cells accumulate during aging, causing chronic inflammation (van Deursen, 2014), cilengitide could be a potential therapeutic route to block inflammation without affecting proliferation in aging.

In summary, here, we provide evidence for the β3 subunit being a marker and regulator of senescence. Our results demonstrate the importance of FA complex formation regulating the microenvironment during senescence activation and identify integrins as potential therapeutic targets to promote healthy aging.

EXPERIMENTAL PROCEDURES

The care and use of mice were in accordance with the UK Home Office regulations and the UK Animals (Scientific Procedures) Act of 1986.

Cell Culture and Retroviral and Lentiviral Infections

MCF7, SK-HEP-1, and IMR-90 were obtained from the American Type Culture Collection. BFs were described previously (Pemberton et al., 2014). Donor primary human fibroblasts were obtained from the Coriell Cell Repository. Cells were maintained in DMEM (Invitrogen) with 10% fetal bovine serum (FBS) (PAA Laboratories) and 1% antibiotic-antimycotic solution (Invitrogen). Mouse hepatic stellate cells were maintained in the same media supplemented with 1 μg/mL Dox. Methods used for retrovirus and lentivirus production and infection have been previously described (O'Loghlen et al., 2012).

Treatment with Kinase Inhibitors

BFs were seeded at the same density in 96-well or 24-well plates. Inhibitors for different signaling pathways were added at the concentrations detailed in Table S2. BFs were incubated with the inhibitors for 48 hr, renewing after 24 hr. Cells were fixed 24 hr later.

Conditioned Media Experiments

The indicated cells were cultured for 7 days in DMEM in 0.5% FBS. The conditioned media (CM) were collected and supplemented to generate 10% FBS CM, and normal cells were treated with or without pan-specific TGF-β1-3 antibodies or the TGFBR1 inhibitor (4 μM) for 72 hr. For the immunoblotting, CM was concentrated using Amicon Ultra Centrifugal Filters (Millipore) and stained with IL-8/IL-6 antibodies.

Statistics

Results are expressed as the mean ± SD, and statistical analysis was performed using a Student's t test. A $p < 0.05$ was considered significant. *$p < 0.05$; **$p < 0.01$; ***$p < 0.001$.

ACCESSION NUMBERS

The accession number for the proteomics dataset reported in this paper is PRIDE: PXD005717.

SUPPLEMENTAL INFORMATION

Supplemental Information includes Supplemental Experimental Procedures, seven figures, and two tables and can be found with this article online at http://dx.doi.org/10.1016/j.celrep.2017.02.012.

AUTHOR CONTRIBUTIONS

V.R. performed most of the experiments, except where specified. A.O., V.E., and A.P.S. performed and analyzed the SILAC data. CDK4/6 inhibitor experiments were performed by M.B., V.M., and A.L. A.O. conceived and designed the study and analyzed most data. A.O. wrote and edited the manuscript, with input from all the authors.

ACKNOWLEDGMENTS

We are grateful to Sharon Brookes, Nadine Martin, John Marshall, Kairbaan Hodivala-Dilke, Jesus Gil, and Cleo Bishop for scientific discussions and critical reading of the manuscript. We thank the tissue bank ShARM, UK, for providing mouse tissue and the Coriell Institute for providing human primary fibroblasts from human donors. Scott Lowe provided the mHSCs. ITGB3 wild-type and mutant plasmids were a kind gift from Mark Ginsberg and William Schiemann. A.O.'s lab is supported by QMUL, MRC (MR/K501372/1) and BBSRC (BB/P000223/1).

Received: August 8, 2016
Revised: December 6, 2016
Accepted: January 31, 2017
Published: March 7, 2017

REFERENCES

Acosta, J.C., O'Loghlen, A., Banito, A., Guijarro, M.V., Augert, A., Raguz, S., Fumagalli, M., Da Costa, M., Brown, C., Popov, N., et al. (2008). Chemokine signaling via the CXCR2 receptor reinforces senescence. Cell *133*, 1006–1018.

Acosta, J.C., Banito, A., Wuestefeld, T., Georgilis, A., Janich, P., Morton, J.P., Athineos, D., Kang, T.W., Lasitschka, F., Andrulis, M., et al. (2013). A complex secretory program orchestrated by the inflammasome controls paracrine senescence. Nat. Cell Biol. *15*, 978–990.

Asano, Y., Ihn, H., Yamane, K., Jinnin, M., Mimura, Y., and Tamaki, K. (2005). Increased expression of integrin alpha(v)beta3 contributes to the establishment of autocrine TGF-beta signaling in scleroderma fibroblasts. J. Immunol. *175*, 7708–7718.

Asselin-Labat, M.L., Sutherland, K.D., Barker, H., Thomas, R., Shackleton, M., Forrest, N.C., Hartley, L., Robb, L., Grosveld, F.G., van der Wees, J., et al. (2007). Gata-3 is an essential regulator of mammary-gland morphogenesis and luminal-cell differentiation. Nat. Cell Biol. *9*, 201–209.

Baker, D.J., Childs, B.G., Durik, M., Wijers, M.E., Sieben, C.J., Zhong, J., Saltness, R.A., Jeganathan, K.B., Verzosa, G.C., Pezeshki, A., et al. (2016). Naturally occurring p16(Ink4a)-positive cells shorten healthy lifespan. Nature *530*, 184–189.

Biran, A., Perelmutter, M., Gal, H., Burton, D.G., Ovadya, Y., Vadai, E., Geiger, T., and Krizhanovsky, V. (2015). Senescent cells communicate via intercellular protein transfer. Genes Dev. *29*, 791–802.

Bollard, J., Miguela, V., Ruiz de Galarreta, M., Venkatesh, A., Bian, C.B., Roberto, M.P., Tovar, V., Sia, D., Molina-Sánchez, P., Nguyen, C.B., et al. (2016). Palbociclib (PD-0332991), a selective CDK4/6 inhibitor, restricts tumour growth in preclinical models of hepatocellular carcinoma. Gut, Published online November 14, 2016. http://dx.doi.org/10.1136/gutjnl-2016-312268.

Brooks, P.C., Montgomery, A.M., Rosenfeld, M., Reisfeld, R.A., Hu, T., Klier, G., and Cheresh, D.A. (1994). Integrin alpha v beta 3 antagonists promote tumor regression by inducing apoptosis of angiogenic blood vessels. Cell *79*, 1157–1164.

Desgrosellier, J.S., and Cheresh, D.A. (2010). Integrins in cancer: biological implications and therapeutic opportunities. Nat. Rev. Cancer *10*, 9–22.

Desgrosellier, J.S., Barnes, L.A., Shields, D.J., Huang, M., Lau, S.K., Prévost, N., Tarin, D., Shattil, S.J., and Cheresh, D.A. (2009). An integrin alpha(v)beta(3)-c-Src oncogenic unit promotes anchorage-independence and tumor progression. Nat. Med. *15*, 1163–1169.

Franovic, A., Elliott, K.C., Seguin, L., Camargo, M.F., Weis, S.M., and Cheresh, D.A. (2015). Glioblastomas require integrin αvβ3/PAK4 signaling to escape senescence. Cancer Res. *75*, 4466–4473.

Fridman, A.L., and Tainsky, M.A. (2008). Critical pathways in cellular senescence and immortalization revealed by gene expression profiling. Oncogene *27*, 5975–5987.

Gil, J., and O'Loghlen, A. (2014). PRC1 complex diversity: where is it taking us? Trends Cell Biol. *24*, 632–641.

Gutiérrez-Fernández, A., Soria-Valles, C., Osorio, F.G., Gutiérrez-Abril, J., Garabaya, C., Aguirre, A., Fueyo, A., Fernández-García, M.S., Puente, X.S., and López-Otín, C. (2015). Loss of MT1-MMP causes cell senescence and nuclear defects which can be reversed by retinoic acid. EMBO J. *34*, 1875–1888.

Hoare, M., Ito, Y., Kang, T.W., Weekes, M.P., Matheson, N.J., Patten, D.A., Shetty, S., Parry, A.J., Menon, S., Salama, R., et al. (2016). NOTCH1 mediates a switch between two distinct secretomes during senescence. Nat. Cell Biol. *18*, 979–992.

Hynes, R.O. (2002). Integrins: bidirectional, allosteric signaling machines. Cell *110*, 673–687.

Jun, J.I., and Lau, L.F. (2010). The matricellular protein CCN1 induces fibroblast senescence and restricts fibrosis in cutaneous wound healing. Nat. Cell Biol. *12*, 676–685.

Karlseder, J., Broccoli, D., Dai, Y., Hardy, S., and de Lange, T. (1999). p53- and ATM-dependent apoptosis induced by telomeres lacking TRF2. Science *283*, 1321–1325.

Kren, A., Baeriswyl, V., Lehembre, F., Wunderlin, C., Strittmatter, K., Antoniadis, H., Fässler, R., Cavallaro, U., and Christofori, G. (2007). Increased tumor cell dissemination and cellular senescence in the absence of beta1-integrin function. EMBO J. *26*, 2832–2842.

Krishnamurthy, J., Torrice, C., Ramsey, M.R., Kovalev, G.I., Al-Regaiey, K., Su, L., and Sharpless, N.E. (2004). Ink4a/Arf expression is a biomarker of aging. J. Clin. Invest. *114*, 1299–1307.

Krizhanovsky, V., Yon, M., Dickins, R.A., Hearn, S., Simon, J., Miething, C., Yee, H., Zender, L., and Lowe, S.W. (2008). Senescence of activated stellate cells limits liver fibrosis. Cell *134*, 657–667.

Legate, K.R., Wickström, S.A., and Fässler, R. (2009). Genetic and cell biological analysis of integrin outside-in signaling. Genes Dev. *23*, 397–418.

Liu, H., Radisky, D.C., Yang, D., Xu, R., Radisky, E.S., Bissell, M.J., and Bishop, J.M. (2012). MYC suppresses cancer metastasis by direct transcriptional silencing of αv and β3 integrin subunits. Nat. Cell Biol. *14*, 567–574.

Loftus, J.C., O'Toole, T.E., Plow, E.F., Glass, A., Frelinger, A.L., 3rd, and Ginsberg, M.H. (1990). A beta 3 integrin mutation abolishes ligand binding and alters divalent cation-dependent conformation. Science *249*, 915–918.

López-Otín, C., Blasco, M.A., Partridge, L., Serrano, M., and Kroemer, G. (2013). The hallmarks of aging. Cell *153*, 1194–1217.

Lujambio, A., Akkari, L., Simon, J., Grace, D., Tschaharganeh, D.F., Bolden, J.E., Zhao, Z., Thapar, V., Joyce, J.A., Krizhanovsky, V., and Lowe, S.W. (2013). Non-cell-autonomous tumor suppression by p53. Cell *153*, 449–460.

Macias, M.J., Martin-Malpartida, P., and Massagué, J. (2015). Structural determinants of Smad function in TGF-β signaling. Trends Biochem. Sci. *40*, 296–308.

Margadant, C., and Sonnenberg, A. (2010). Integrin-TGF-beta crosstalk in fibrosis, cancer and wound healing. EMBO Rep. *11*, 97–105.

Morey, L., Aloia, L., Cozzuto, L., Benitah, S.A., and Di Croce, L. (2013). RYBP and Cbx7 define specific biological functions of polycomb complexes in mouse embryonic stem cells. Cell Rep. *3*, 60–69.

Muñoz-Espín, D., and Serrano, M. (2014). Cellular senescence: from physiology to pathology. Nat. Rev. Mol. Cell Biol. *15*, 482–496.

Muñoz-Espín, D., Cañamero, M., Maraver, A., Gómez-López, G., Contreras, J., Murillo-Cuesta, S., Rodríguez-Baeza, A., Varela-Nieto, I., Ruberte, J., Collado, M., and Serrano, M. (2013). Programmed cell senescence during mammalian embryonic development. Cell *155*, 1104–1118.

O'Loghlen, A., Muñoz-Cabello, A.M., Gaspar-Maia, A., Wu, H.A., Banito, A., Kunowska, N., Racek, T., Pemberton, H.N., Beolchi, P., Lavial, F., et al. (2012). MicroRNA regulation of Cbx7 mediates a switch of Polycomb orthologs during ESC differentiation. Cell Stem Cell *10*, 33–46.

O'Loghlen, A., Brookes, S., Martin, N., Rapisarda, V., Peters, G., and Gil, J. (2015a). CBX7 and miR-9 are part of an autoregulatory loop controlling p16(INK) (4a). Aging Cell *14*, 1113–1121.

O'Loghlen, A., Martin, N., Krusche, B., Pemberton, H., Alonso, M.M., Chandler, H., Brookes, S., Parrinello, S., Peters, G., and Gil, J. (2015b). The nuclear receptor NR2E1/TLX controls senescence. Oncogene *34*, 4069–4077.

Parrinello, S., Coppe, J.P., Krtolica, A., and Campisi, J. (2005). Stromal-epithelial interactions in aging and cancer: senescent fibroblasts alter epithelial cell differentiation. J. Cell Sci. *118*, 485–496.

Pemberton, H., Anderton, E., Patel, H., Brookes, S., Chandler, H., Palermo, R., Stock, J., Rodriguez-Niedenführ, M., Racek, T., de Breed, L., et al. (2014). Genome-wide co-localization of Polycomb orthologs and their effects on gene expression in human fibroblasts. Genome Biol. *15*, R23.

Pérez-Mancera, P.A., Young, A.R., and Narita, M. (2014). Inside and out: the activities of senescence in cancer. Nat. Rev. Cancer *14*, 547–558.

Ressler, S., Bartkova, J., Niederegger, H., Bartek, J., Scharffetter-Kochanek, K., Jansen-Dürr, P., and Wlaschek, M. (2006). p16INK4A is a robust in vivo biomarker of cellular aging in human skin. Aging Cell *5*, 379–389.

Reynolds, L.E., Conti, F.J., Lucas, M., Grose, R., Robinson, S., Stone, M., Saunders, G., Dickson, C., Hynes, R.O., Lacy-Hulbert, A., and Hodivala-Dilke, K. (2005). Accelerated re-epithelialization in beta3-integrin-deficient- mice is associated with enhanced TGF-beta1 signaling. Nat. Med. *11*, 167–174.

Salama, R., Sadaie, M., Hoare, M., and Narita, M. (2014). Cellular senescence and its effector programs. Genes Dev. *28*, 99–114.

Schmierer, B., and Hill, C.S. (2007). TGFbeta-SMAD signal transduction: molecular specificity and functional flexibility. Nat. Rev. Mol. Cell Biol. *8*, 970–982.

Seguin, L., Kato, S., Franovic, A., Camargo, M.F., Lesperance, J., Elliott, K.C., Yebra, M., Mielgo, A., Lowy, A.M., Husain, H., et al. (2014). An integrin β_3-KRAS-RalB complex drives tumour stemness and resistance to EGFR inhibition. Nat. Cell Biol. *16*, 457–468.

Seguin, L., Desgrosellier, J.S., Weis, S.M., and Cheresh, D.A. (2015). Integrins and cancer: regulators of cancer stemness, metastasis, and drug resistance. Trends Cell Biol. *25*, 234–240.

Serrano, M., Lin, A.W., McCurrach, M.E., Beach, D., and Lowe, S.W. (1997). Oncogenic ras provokes premature cell senescence associated with accumulation of p53 and p16INK4a. Cell *88*, 593–602.

Storer, M., Mas, A., Robert-Moreno, A., Pecoraro, M., Ortells, M.C., Di Giacomo, V., Yosef, R., Pilpel, N., Krizhanovsky, V., Sharpe, J., and Keyes, W.M. (2013). Senescence is a developmental mechanism that contributes to embryonic growth and patterning. Cell *155*, 1119–1130.

Stupack, D.G., Puente, X.S., Boutsaboualoy, S., Storgard, C.M., and Cheresh, D.A. (2001). Apoptosis of adherent cells by recruitment of caspase-8 to unligated integrins. J. Cell Biol. *155*, 459–470.

Tkach, M., and Théry, C. (2016). Communication by extracellular vesicles: where we are and where we need to go. Cell *164*, 1226–1232.

van Deursen, J.M. (2014). The role of senescent cells in ageing. Nature *509*, 439–446.

Cell Reports, Volume *18*

Supplemental Information

Integrin Beta 3 Regulates Cellular Senescence by Activating the TGF-β Pathway

Valentina Rapisarda, Michela Borghesan, Veronica Miguela, Vesela Encheva, Ambrosius P. Snijders, Amaia Lujambio, and Ana O'Loghlen

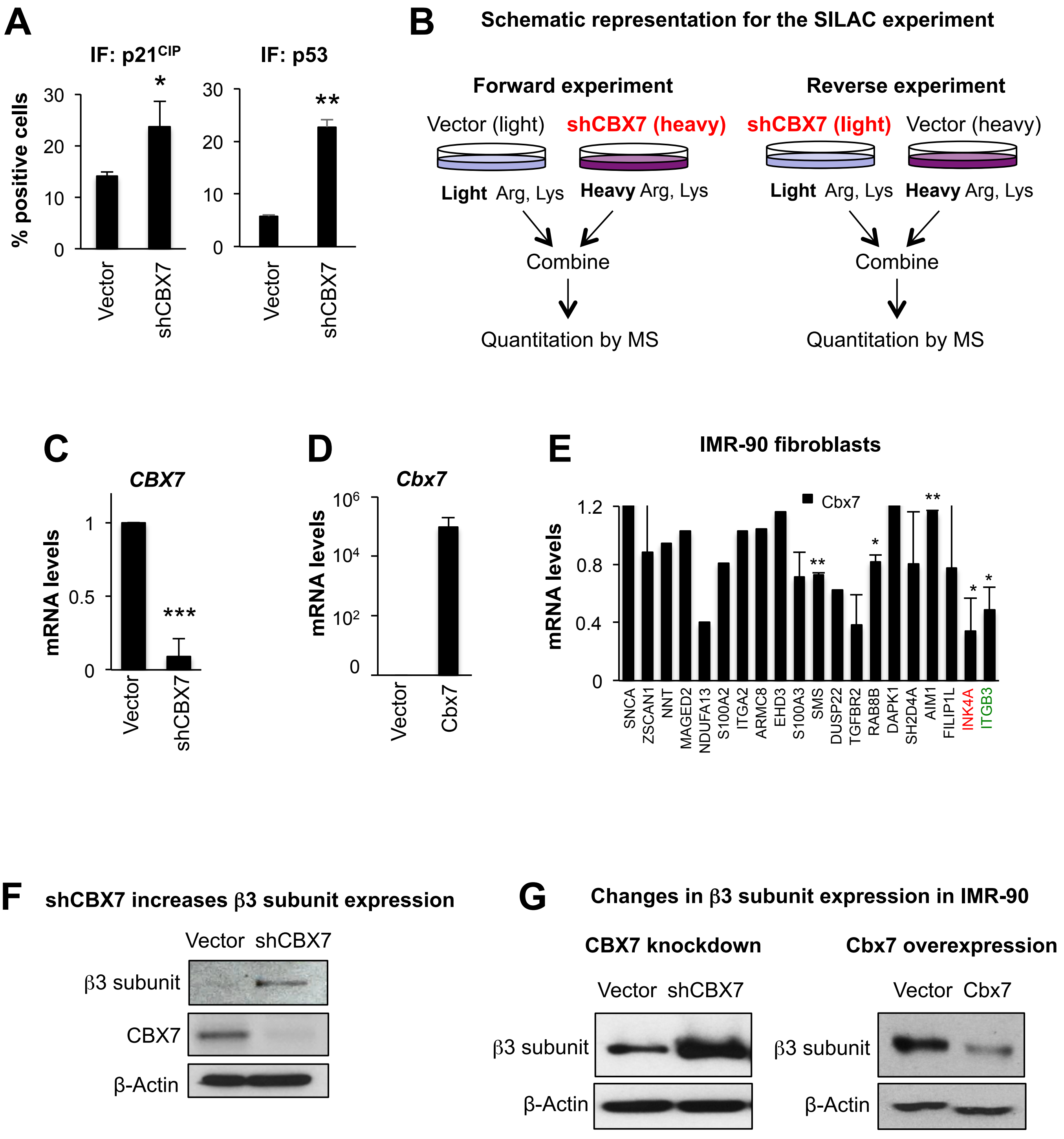

A
IF: p21CIP
IF: p53
% positive cells
Vector
shCBX7
B
Schematic representation for the SILAC experiment
Forward experiment
Reverse experiment
Vector (light)
shCBX7 (heavy)
shCBX7 (light)
Vector (heavy)
Light Arg, Lys
Heavy Arg, Lys
Combine
Quantitation by MS
C
CBX7
mRNA levels
D
Cbx7
E
IMR-90 fibroblasts
SNCA
ZSCAN1
NNT
MAGED2
NDUFA13
S100A2
ITGA2
ARMC8
EHD3
S100A3
SMS
DUSP22
TGFBR2
RAB8B
DAPK1
SH2D4A
AIM1
FILIP1L
INK4A
ITGB3
F
shCBX7 increases β3 subunit expression
Vector shCBX7
β3 subunit
CBX7
β-Actin
G
Changes in β3 subunit expression in IMR-90
CBX7 knockdown
Cbx7 overexpression
Vector Cbx7

Figure S1. CBX7 knockdown induces senescence in human primary breast fibroblasts (Related to Figures 1 and 2)

(A) Knockdown of *CBX7* (shCBX7) in BF fibroblasts induces an increase in the percentage of cells staining positive for p53 and p21^{CIP}. (B) Scheme for the strategy followed to perform the SILAC experiment. In the forward experiment, we grow BF infected with shCBX7 in the media containing "heavy aminoacids", whereas in the reverse experiment we culture shCBX7 cells in the media with "light aminoacids". (C) qPCR showing *CBX7* knockdown efficiency at the mRNA level in BF transduced with shCBX7. (D) Ectopic expression of mouse Cbx7 by retroviral transduction shows an increase in Cbx7 mRNA by qPCR. (E) qPCR analyses show that Cbx7 expression downregulates the genes of the SILAC proteins in IMR-90 fibroblasts. Data is normalized to the control, and represent the mean ± SD of 1-3 independent experiments. (F) Representative immunoblot showing an increase in β3 subunit levels, concomitant with a decrease in CBX7 protein levels in BF transduced with shCBX7. β-Actin is used as loading control. (G) Representative immunoblot showing changes in β3 subunit levels upon CBX7 knockdown or overexpression in IMR-90 fibroblasts. β-Actin is used as loading control.

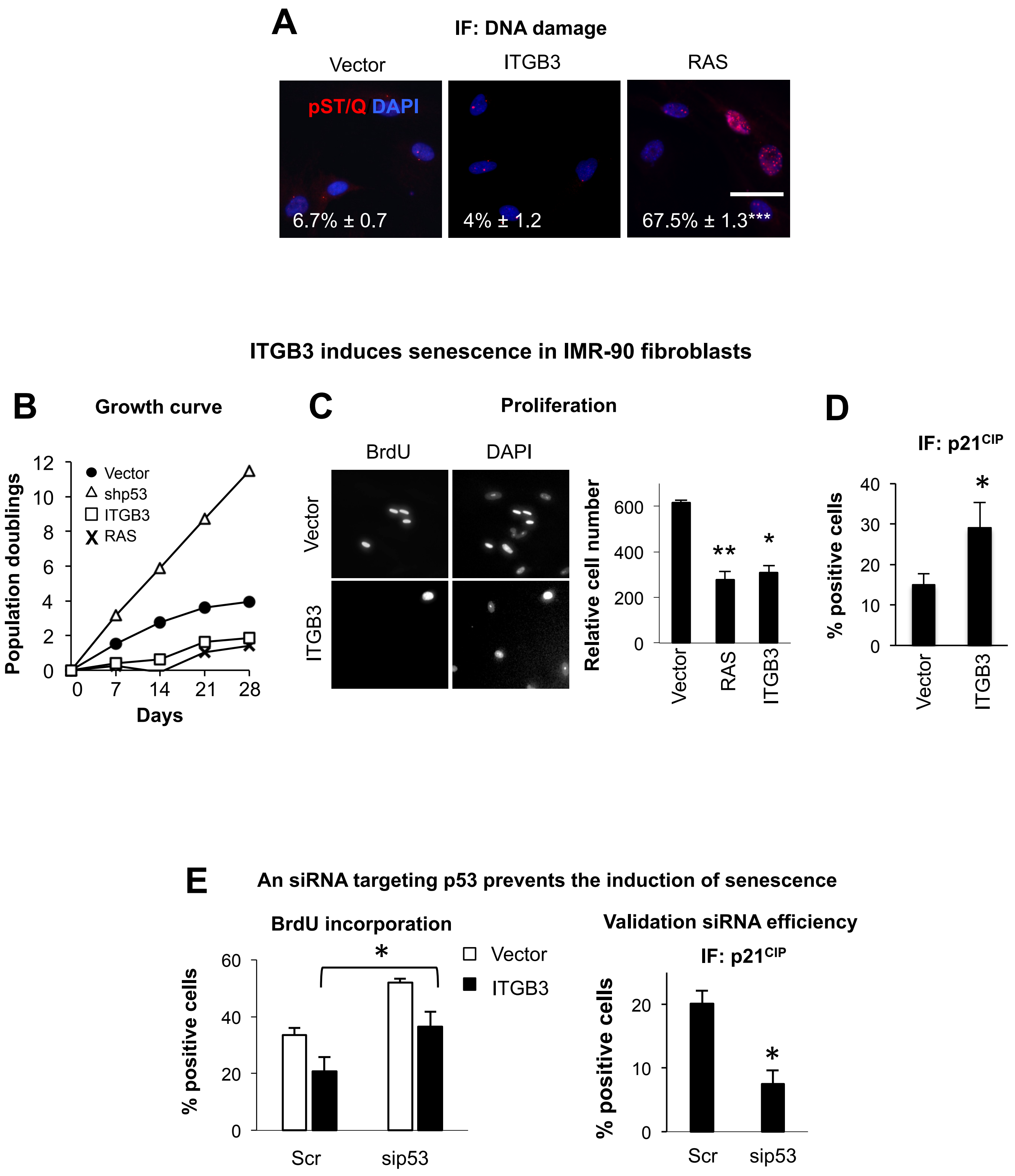
A
IF: DNA damage
Vector
ITGB3
RAS
pST/Q DAPI
6.7% ± 0.7
4% ± 1.2
67.5% ± 1.3***
ITGB3 induces senescence in IMR-90 fibroblasts
B
Growth curve
Population doublings
Days
Vector
shp53
ITGB3
RAS
C
Proliferation
BrdU
DAPI
Vector
ITGB3
Relative cell number
Vector
RAS
ITGB3
D
IF: p21CIP
% positive cells
Vector
ITGB3
E
An siRNA targeting p53 prevents the induction of senescence
BrdU incorporation
% positive cells
Vector
ITGB3
Scr
sip53
Validation siRNA efficiency
IF: p21CIP
% positive cells
Scr
sip53

Figure S2. ITGB3 induces senescence dependent on the p53 pathway (Related to Figure 3)
(A) BF expressing ITGB3 do not display DNA damage, measured by pST/Q staining, and calculated by quantifying the percentage of cells positive for pST/Q staining. RAS was used as a positive control for DNA-damage in senescence. (B) IMR-90 primary fibroblasts transduced with a vector encoding ITGB3 show reduced proliferation, measured by calculating the population doubling growth curve. H-Ras^{G12V} (RAS) and shp53 expressing fibroblasts were used as positive and negative regulators of senescence, respectively. (C) A reduction in the proliferation rate is also shown in representative pictures for BrdU incorporation (left panel) and by measuring the relative cell numbers (right panel). (D) IMR-90 fibroblasts expressing ITGB3 also show an increase in the percentage of cells staining positive for p21^{CIP} protein by IF. (E) Disruption of *TP53* mRNA prevents the activation of senescence induced by the overexpression of ITGB3. BF expressing either Vector or ITGB3 were reverse-transfected with a Scramble (Scr) RNAi or an siRNA targeting p53 (sip53). Left panel: proliferation was quantified by measuring the percentage of cells incorporating BrdU. Right panel: Knockdown efficiency for sip53 was quantified by measuring the levels of p21^{CIP}, a target of p53. Data represents the mean ± SD of 2-3 independent experiments. Scale bar, 50μm

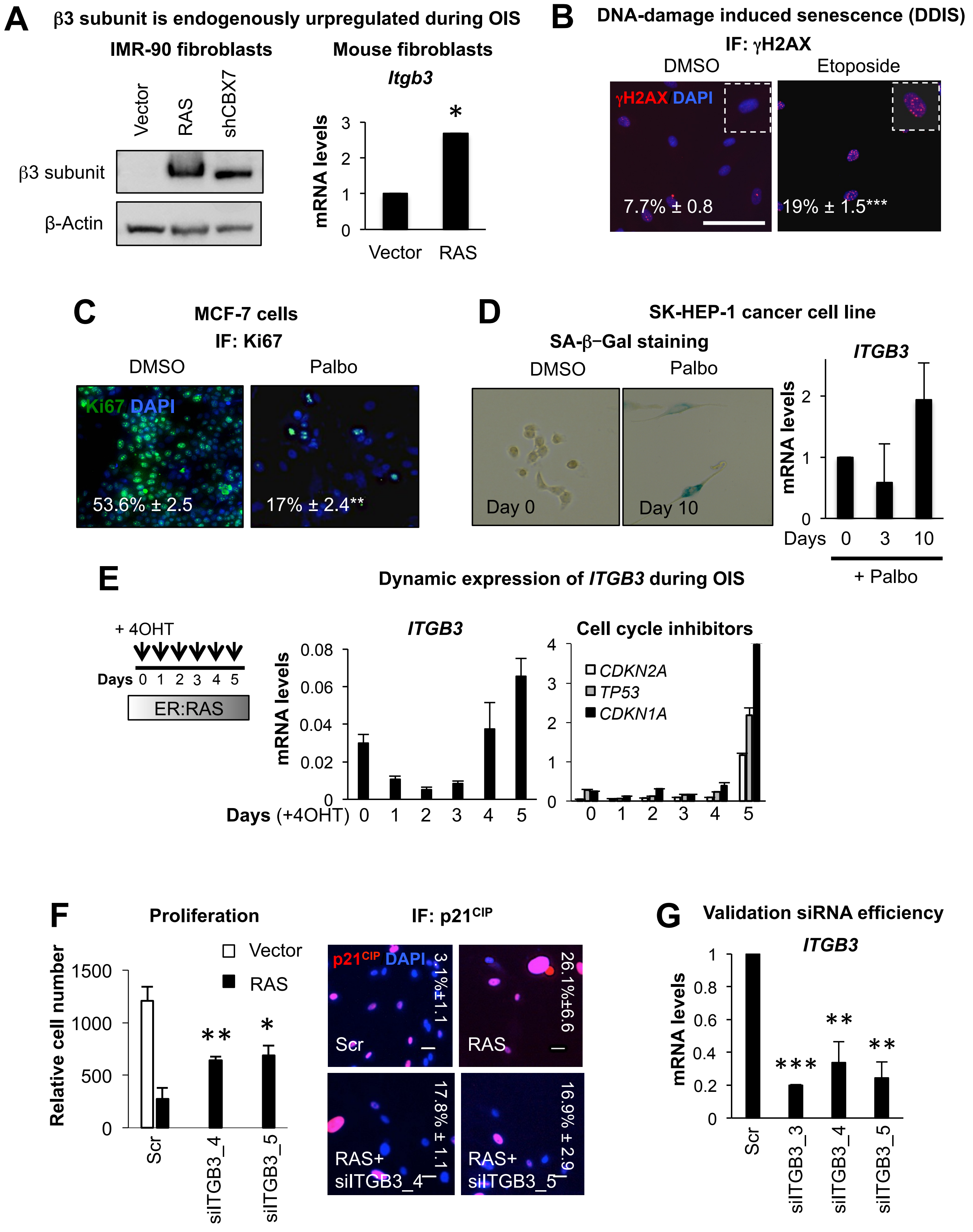
A
β3 subunit is endogenously urpregulated during OIS
IMR-90 fibroblasts
Vector
RAS
shCBX7
β3 subunit
β-Actin
Mouse fibroblasts
Itgb3
mRNA levels
*
Vector
RAS
B
DNA-damage induced senescence (DDIS)
IF: γH2AX
DMSO
Etoposide
γH2AX DAPI
7.7% ± 0.8
19% ± 1.5***
C
MCF-7 cells
IF: Ki67
DMSO
Palbo
Ki67 DAPI
53.6% ± 2.5
17% ± 2.4**
D
SK-HEP-1 cancer cell line
SA-β-Gal staining
DMSO
Palbo
Day 0
Day 10
ITGB3
mRNA levels
Days
+ Palbo
E
Dynamic expression of ITGB3 during OIS
+ 4OHT
Days
ER:RAS
ITGB3
mRNA levels
Days (+4OHT)
Cell cycle inhibitors
CDKN2A
TP53
CDKN1A
F
Proliferation
Vector
RAS
Relative cell number
Scr
siITGB3_4
siITGB3_5
IF: p21CIP
p21CIP DAPI
3.1%±1.1
26.1%±6.6
17.8% ± 1.1
16.9% ± 2.9
Scr
RAS
RAS+ siITGB3_4
RAS+ siITGB3_5
G
Validation siRNA efficiency
ITGB3
mRNA levels
Scr
siITGB3_3
siITGB3_4
siITGB3_5

Figure S3. ***ITGB3*** **is a regulator of cellular senescence (Related to Figure 4)**
(A) Left panel shows immunoblot for β3 subunit in IMR-90 fibroblasts expressing RAS and shCBX7. β−Actin is used as loading control; right panel shows relative *Itgb3* mRNA levels upon RAS expression in mouse embryonic fibroblasts (MEFs) measured by qPCR. A representative qPCR is shown. (B) BF treated with etoposide show an increase in DNA-damage. This was measured by staining with a phospho-γ-H2AX antibody by IF. The quantification represents the percentage of cells with positive staining. Scale bar, 50μm. (C) MCF7 breast cancer cells were treated with 200nM Palbociclib for 7 days. Proliferation was measured by staining with a Ki67 antibody. Data shows the percentage of cells staining positive for Ki67. (D) SK-HEP-1 hepatocellular carcinoma cells were treated with 1μM Palbo for 3 or 10 days and stained for SA-β-Gal (left) or subjected to qPCR analyses for *ITGB3* mRNA levels (right panel). (E) *ITGB3* is dynamically regulated during cellular senescence. Time-course treatment with 200nM 4-Hydroxytamoxifen (4OHT) of fibroblasts harboring an ER:RAS construct. RNA was collected at different time points and subjected to qPCR analysis to determine *ITGB3*, *CDKN2A*, *TP53* and *CDKN1A* levels. (F) BF expressing vector or RAS were transfected with a scramble siRNA (Scr) or two different siRNA against ITGB3 (siITGB3_4 and 5). 4 days after transfection, BrdU was added and 24h later BrdU incorporation and $p21^{CIP}$ levels were assessed. Left panel: relative cell number. Right panel: representative images for $p21^{CIP}$ staining by IF. Scale bar, 100μm. (G) qPCR showing mRNA levels for *ITGB3* in BF fibroblasts transfected with three independent siRNA targeting *ITGB3* (siITGB3).

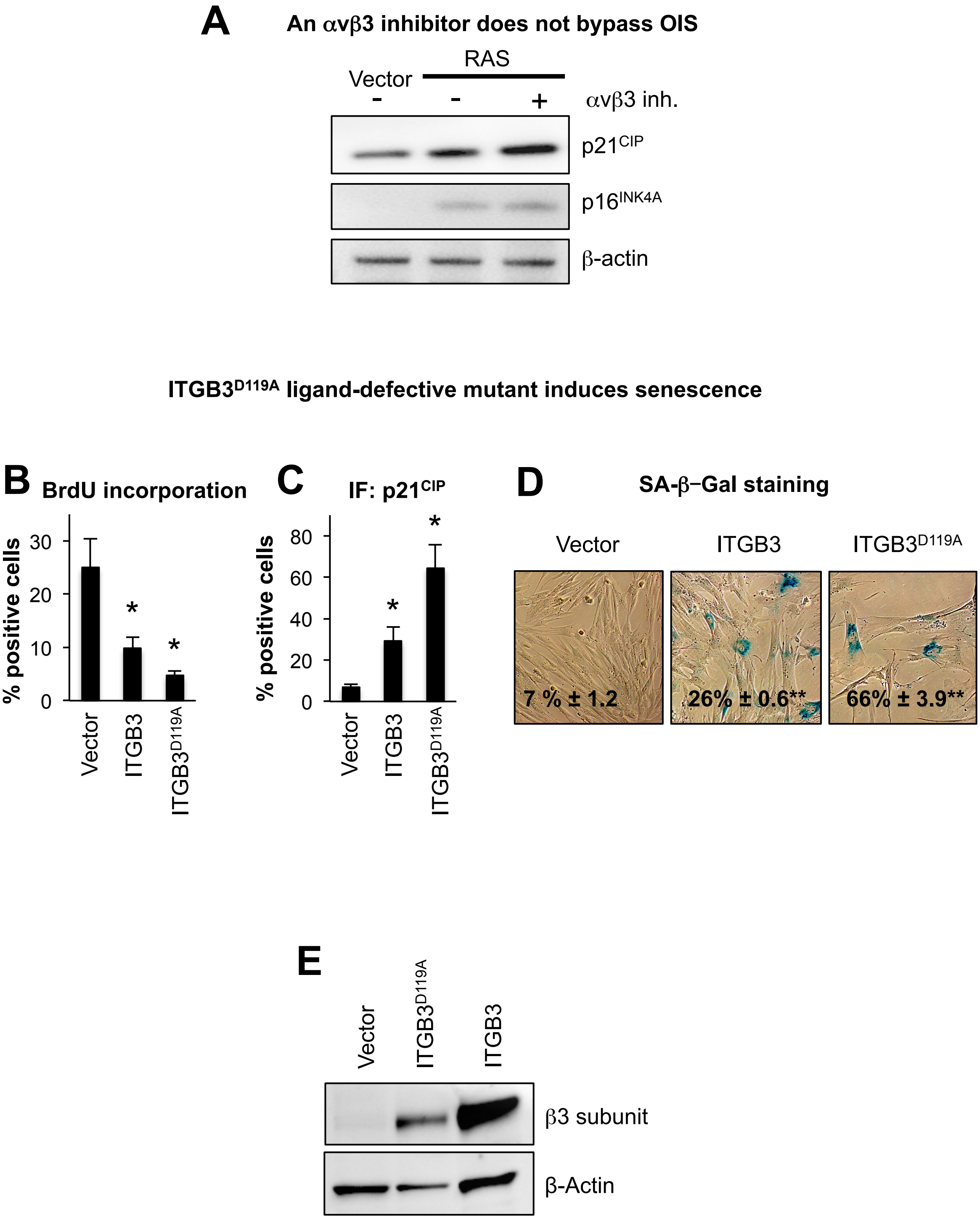
A
An αvβ3 inhibitor does not bypass OIS
RAS
Vector
- - +
αvβ3 inh.
p21CIP
p16INK4A
β-actin
ITGB3D119A ligand-defective mutant induces senescence
B
BrdU incorporation
% positive cells
30
20
10
0
Vector
ITGB3
ITGB3D119A
*
*
C
IF: p21CIP
% positive cells
80
60
40
20
0
Vector
ITGB3
ITGB3D119A
*
*
D
SA-β−Gal staining
Vector
ITGB3
ITGB3D119A
7 % ± 1.2
26% ± 0.6**
66% ± 3.9**
E
Vector
ITGB3D119A
ITGB3
β3 subunit
β-Actin

Figure S4. ITGB3 induces senescence independently of its ligand-binding activity (Related to Figure 4)

(A) Immunoblot for $p21^{CIP}$ and $p16^{INK4A}$ protein levels in BF cells expressing either Vector or RAS. RAS cells were treated with DMSO or 50nM of an $\alpha v\beta 3$ inhibitor (cilengitide) for 48h, washed and followed by 72h of fresh media incubation. β-actin is shown as loading control. (B-D) Expression of an ITGB3 mutant construct ($ITGB3^{D119A}$), defective for ligand-binding activity, induces senescence in BF fibroblasts. ITGB3 wild type is used as control. (B) BrdU proliferation is reduced in cells expressing $ITGB3^{D119A}$, while (C) $p21^{CIP}$ protein levels and the (D) the percentage of cells staining positive for SA-β-Gal are increased. (E) Immunoblot for β3 subunit shows the expression levels for ITGB3 wild-type and mutant ($ITGB3^{D119A}$) construct.

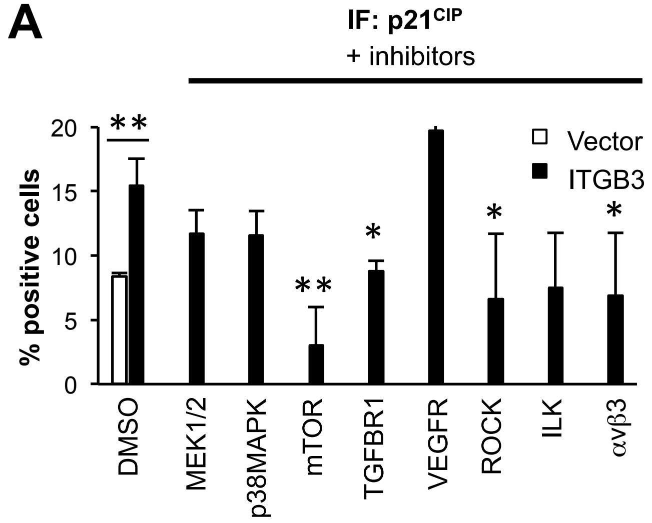
A
IF: p21CIP
+ inhibitors
% positive cells
20
15
10
5
0
**
Vector
ITGB3
**
*
*
*
DMSO
MEK1/2
p38MAPK
mTOR
TGFBR1
VEGFR
ROCK
ILK
αvβ3

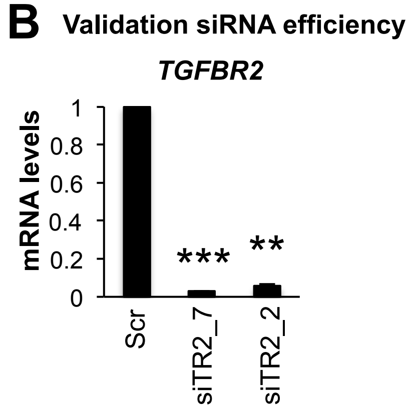
B
Validation siRNA efficiency
TGFBR2
mRNA levels
1
0.8
0.6
0.4
0.2
0
***
**
Scr
siTR2_7
siTR2_2

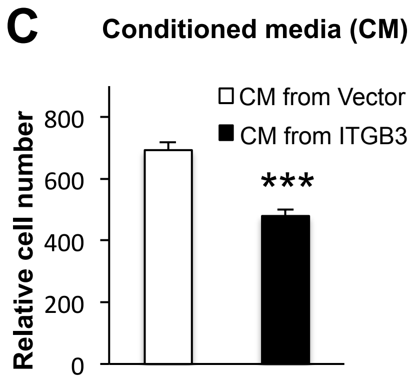
C
Conditioned media (CM)
Relative cell number
800
600
400
200
0
CM from Vector
CM from ITGB3
***

Figure S5. ITGB3 induces senescence in a cell and non-cell autonomous fashion by activating the TGFβ pathway (Related to Figure 5)
(A) BF expressing vector or ITGB3 were treated for 48h with a small molecule drug screen. The percentage of $p21^{CIP}$ positive cells with or without the inhibitors is shown. The graph indicates the inhibitor's targets. Inhibitor's details are: 40μM PD98059 (targeting MEK1/2), 20μM SB202190 (p38MAPK), 100nM TORIN2 (mTOR), 4μM TGFβ-R1 (TGFBR1), 8μM Vegfr-2/Flt3/C-Kit (VEGFR), 150nM GSK429286A (ROCK1/2, Rho-associated kinase), 50nM Cpd22 (ILK, integrin-linked kinase) and 50nM Cilengitide (αvβ3). (B) qPCR analyses to show the mRNA knockdown efficiency of the RNAi targeting *TGFBR2* (siTR2_7 and siTR_2). (C) Conditioned media (CM) from BF expressing either Vector or ITGB3 was used to treat normal BF cells. Conditioned media was produced in 0.5% FBS for 7 days. The relative cell number was calculated after treating the cells for 72h with the conditioned media.

Rapisarda *et al.* **Figure S6**

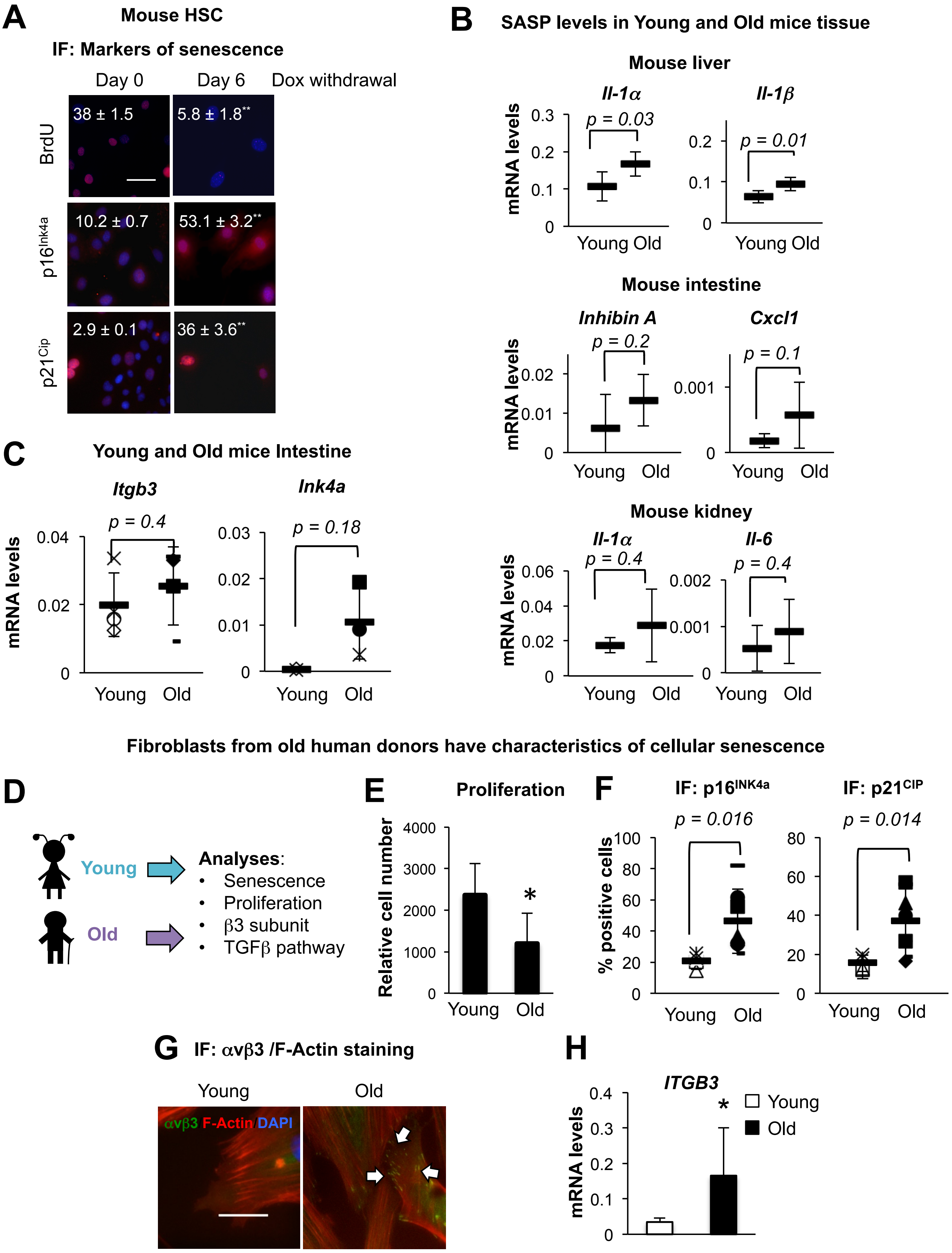

Figure S6. ITGB3 is expressed during replicative senescence and aging in human and mouse (Related to Figure 6)

(A) IF analysis for senescence markers (BrdU, $p16^{Ink4a}$ and $p21^{Cip}$) in mouse Hepatic Stellate Cells (mHSC) upon different days of doxycycline (Dox) withdrawal. Quantification represents the percentage of cells staining positive for each antibody. (B) Additional markers of senescence/aging - different SASP mRNAs - were determined in different tissues (liver, intestine, and kidney) from mice aged 4 (Young) and 25 (Old) months. (C) Intestines from young (4 months) and old (25 months) C57BL/6J mice were subjected to qPCR to determine *Ink4a* and *Itgb3* mRNA expression levels. (D) Schematic representation of the strategy followed to determine the implication of the β3 subunit in aging in human fibroblasts. We analyzed primary fibroblasts from young (∼10 years) and old (∼80 years) human donors to check for markers of senescence, β3 subunit and regulators of TGFβ. (E) Fibroblasts from old donors (n=7 donors) show a lower proliferation rate than fibroblasts from young donors (n=4 donors). (F) Fibroblasts from old donors present different markers for senescence. Graphs represent the percentage of cells stained positive for $p16^{INK4A}$ and $p21^{CIP}$ in young and old fibroblasts. Data represents the mean ± SD of fibroblasts derived from 4 young and 7 old donors. (G) Representative images for αvβ3 (green) and F-Actin (red) staining in young and old donor fibroblasts. The formation of FA is shown with white arrows in the old fibroblasts. Scale bar, 100 μm. (H) qPCR analysis for *ITGB3* mRNA levels in fibroblasts from young and old donors.

A **RNAi targeting ITGB3 reverses aging**

IF: Markers of senescence

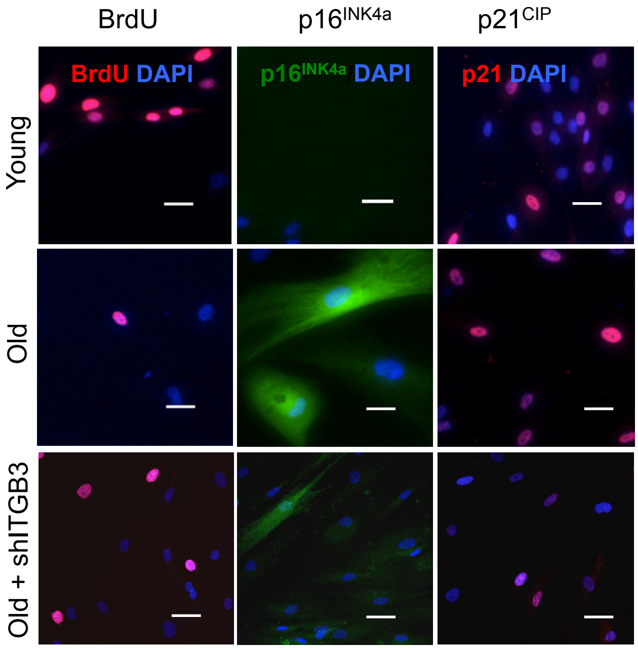

Figure S7. Knockdown of *ITGB3* using a specific shRNA (shITGB3) averts senescence/aging markers in fibroblasts derived from old donors (Related to Figure7)

(A) IF analysis for different markers of senescence/aging (BrdU, $p16^{INK4A}$ and $p21^{CIP}$) in fibroblasts from old donors expressing an shRNA targeting *ITGB3* (shITGB3). Cells from young donors were used as controls.

SUPPLEMENTAL EXPERIMENTAL PROCEDURES

Antibodies for immunofluorescence, immunoblotting and ChIP.
Details of the antibodies used can be found in the Supplemental Table S2.

qPCR, immunoblotting and immunofluorescence
Total RNA was extracted using Trizol Reagent (Invitrogen) according to the manufacturer's protocol. cDNA was generated using the High Capacity cDNA Reverse transcription kit (Invitrogen). For specific primers see the Supplemental Table S2. Protein extracts were processed and analyzed as before (O'Loghlen et al., 2012). Immunofluorescence was performed using an InCell Analyzer 1000 (GE). Image processing and quantification was performed using InCell Investigator software (GE) (Acosta et al., 2008). Primers and antibodies used in this study are listed in the Supplemental Table S2.

RNA interference experiments
Indicated cells were transfected with 30nM of siRNA in a 96-well plate or 6-well plate. A 3.5% solution of HiPerFect transfection reagent (QIAGEN) was prepared in serum-free DMEM and then mixed with the siRNA. The mixture was incubated for 30 minutes at room temperature and then added to the cells. The medium was changed after 24 hours and cells were incubated for additional 24 hours before being processed for IF analysis or RNA/protein isolation. For RNAi targeting integrins, the forward transfection method was use, where the cells are plated first, senescence was induced and the siRNA/HiPerFect mixture was added after senescence was established. Experiments using an siRNA targeting p53 were reverse transfected, where the siRNA/HiPerFect mixture was added at the same time as cells were plated.

SA-β-Galactosidase staining
Cells were seeded at the same density and after 72h fixed with 0.5% Glutaraldehyde, washed with 1mM $MgCl_2$ pH 6.0 and stained with X-Gal staining solution (1mM $MgCl_2$ solution, 1X KC solution and X-Gal). Cells were incubated from 4h up to overnight at 37°C and stained with DAPI. The analysis of the percentage of SA-β-galactosidase positive cells was performed using the plugin Cell Counter in the ImageJ software. SA-β-Gal positive cells were determined as the percentage of cells staining blue (light or dark blue) with respect to the total amount of cells.

Chromatin immunoprecipitation (ChIP)
ChIP assay was performed using BF. Cells were cross-linked in 1% paraformaldehyde for 10 minutes at room temperature. Fixed cells were lysed in Lysis Buffer [50 mM HEPES (pH 7.5), 140 mM NaCl, 1 mM EDTA, 0.1% IGEPAL 630 (Sigma-Aldrich)], containing 0.05% Triton X100, 2.5 % glycerol and supplemented with 1X protease inhibitor cocktail (Roche) for 30 minutes on ice, followed by centrifugation incubation in Buffer 2 [0.1 M Tris HCl (pH 8) and 200 mM NaCl with protease inhibitors] for 30 minutes at room temperature. Chromatin was sonicated as follows: three cycles of 10 minutes each (30 seconds on followed by 30 seconds off). Crosslinked DNA after sonication was precipitated with 5 μg of anti-CBX7, anti-CBX8 and anti-RING1B antibodies or non-immune mouse/rabbit IgG (Abcam) overnight at 4°C. Chromatin/antibody complex was pulled down with Dynal Protein G or DynaI Protein A magnetic beads (Invitrogen) and washed in the low- and high-salt buffers. After de-crosslinking (65°C for 4 hours) and Proteinase K treatment, chromatin was purified by phenol-chloroform extraction and isopropanol precipitation. The antibodies used and qPCR primers are listed in the Supplemental Table S2.

SILAC (Stable Isotope Labeling with Aminoacids in Culture) Mass Spectrometry.
SILAC-labeled vector and shCBX7 fibroblasts were generated as before (O'Loghlen et al., 2012). Protein extracts were separated by SDS-PAGE and subjected to overnight in gel trypsin digestion. Peptide extracts were analyzed using a Q-Exactive mass spectrometer coupled to an Ultimate3000 LC (both

Thermo Fisher) using an Easy Spray Nano-source. The instrument was operated in data dependent acquisition mode selecting the 10 most intense precursor ions for fragmentation. Raw data was processed using MaxQuant/Andromeda as previously described (Cox and Mann, 2008). Outlier detection was performed using the significance B option available in Perseus software with a p-value cut-off of 0.05 for significance. Proteins were selected as significant when a two-fold difference in expression levels was observed. Proteomic data can be found in the Supplemental Table S1.

Pathways analysis KEGG

KEGG pathway analysis was performed on upregulated and downregulated proteins using DAVID Functional Annotation Bioinformatics Microarray Analysis (Huang da et al., 2009).

Mice tissue

All tissues (liver, kidney and intestine) come from female C57BL/6J mice. All mice (4, 19 and 25 month-old) were maintained in the same housing with identical environmental conditions. Tissues were provided by the Tissue Bank provider ShARMUK.

SUPPLEMENTAL REFERENCES

Cox, J., and Mann, M. (2008). MaxQuant enables high peptide identification rates, individualized p.p.b.-range mass accuracies and proteome-wide protein quantification. Nature biotechnology *26*, 1367-1372.

Huang da, W., Sherman, B.T., and Lempicki, R.A. (2009). Systematic and integrative analysis of large gene lists using DAVID bioinformatics resources. Nature protocols *4*, 44-57.

Table S1. Proteins differentially expressed upon shCBX7 (related to Figure 1)

Ratio H/L CB	Ratio H/L no	Ratio H/L CB	Ratio H/L no	PEP	Intensity	Unique pept	Unique pept	Ratio H/L co	Ratio H/L co	Intensity CB	Intensity L C	Intensity H C	Intensity CB	Intensity L C	Intensity H C	p-value FOR	p-value REV	Protein IDs	Protein nam	Gene names	Proteins
1.44631	0.951215	-2.49395	-1.74826	2.40E-63	8.53158	7	9	8	13	8.16035	7.62597	8.01026	8.29097	8.17397	7.66414	0.0292742	0.00089415	P00813;F5G\	Adenosine d	ADA	4
1.71048	1.49687	-1.93838	-1.44957	2.30E-31	7.97017	9	6	9	7	7.87365	7.27788	7.74659	7.26968	7.2029	6.42349	0.00229544	0.021418	Q9Y4K1;B4D	Absent in me	AIM1	9
2.23327	1.77167	-2.64647	-1.89421	8.65E-64	8.72874	10	13	9	13	8.17371	7.51204	8.06696	8.58692	8.45588	8.00268	7.67E-05	0.00020935	P12429;D6R/	Annexin A3;/	ANXA3	6
-1.62629	-2.02421	-2.09456	-1.43546	0	8.086	0	0	2	3	7.81066	7.69509	7.17924	7.7577	7.64607	7.11307	0.00398523	0.00590048	Q86X54;F8WDL0		AP1B1	2
2.37843	1.95832	-3.0517	-2.29301	7.19E-168	8.80721	7	13	6	13	8.32901	7.99498	8.05865	8.63166	8.58	7.68147	1.31E-05	7.60E-06	P02649;H0Y7	Apolipoprote	APOE	11
1.53297	1.05207	-1.85173	-1.29764	4.74E-17	7.85644	4	6	4	8	7.34604	6.78691	7.2058	7.69607	7.56633	7.10813	0.074796	0.0489709	Q8IUR7;A8M	Armadillo rep	ARMC8	17
3.09222	2.55748	1.62138	2.32984	9.01E-05	7.90019	1	1	1	1	7.70547	6.62175	7.66809	7.45808	6.80791	7.34805	2.11E-06	0.00130588	A8K5B0;Q9U	Armadillo rep	ARMCX3	2
2.08726	1.73231	-1.7238	-1.0803	1.49E-06	7.45596	1	2	1	2	6.53507	5.86674	6.43014	7.40045	7.25833	6.84621	0.0134794	0.0826409	A8K9U2;Q9N	Ankyrin repe	ASB6;DKFZp6	4
-1.72681	-2.17456	1.89426	2.51695	1.07E-42	8.13893	6	6	7	6	7.92982	7.81197	7.30576	7.72115	7.04879	7.61734	0.00183179	0.0005141	Q53HH2;P48	ATP synthase	ATP5O	5
1.52226	1.08393	-1.90363	-1.22061	1.12E-60	8.27063	3	4	5	7	7.83996	7.21131	7.72352	8.06934	7.94909	7.45283	0.0211275	0.0187458	A4D1N9;P07	Bisphosphog	BPGM	4
-3.90064	-4.44247	-4.2536	-3.56976	9.82E-45	7.93938	1	1	2	2	7.573	7.55297	6.22691	7.69515	7.68175	6.17791	3.85E-10	6.46E-08	Q4LE82;B0U:	Complement	C4A variant p	23
2.22435	1.63222	-1.70618	-1.1935	3.57E-05	6.84415	1	2	1	3	6.29743	5.56177	6.20922	6.69907	6.59719	6.01945	0.0188489	0.178892	Q8N3F0;B8Z	UPF0452 pro	C7orf41	2
1.6427	1.05561	-3.22729	-2.57184	2.55E-48	7.94642	1	2	2	2	7.50453	6.78245	7.41322	7.75157	7.70671	6.74347	0.0335834	1.04E-06	Q9Y2V2;H3B	Calcium-regu	CARHSP1	5
2.1331	1.74507	-2.70369	-1.961	7.47E-34	8.47756	2	4	5	6	8.03989	7.31767	7.9486	8.28031	8.20809	7.46541	0.00047251	0.00020739	P42574	Caspase-3;Ca	CASP3	1
1.75719	1.27691	-1.79872	-1.13217	3.43E-12	7.9453	0	2	1	4	7.01068	6.35715	6.90163	7.89163	7.77428	7.26597	0.0555932	0.0289067	P55210;B4D(	Caspase-7;Ca	CASP7	5
2.97882	2.48063	-3.48684	-2.7314	3.29E-77	8.80789	8	10	9	11	8.33161	7.46762	8.26773	8.63137	8.56697	7.77075	4.07E-08	1.03E-07	A4D1G0;Q00	Cyclin-depen	CDK6	3
2.54374	2.00928	-3.65634	-3.00069	1.46E-86	8.30895	2	3	8	8	7.88309	7.10209	7.80447	8.10476	8.07936	6.85905	7.14E-05	2.07E-08	P42771;D1LY	Cyclin-depen	CDKN2A	7
-1.64395	-2.12218	-3.18298	-2.37864	3.60E-26	7.66251	1	1	1	1	7.00143	6.89175	6.35011	7.55559	7.50556	6.5922	0.0378447	0.00031347	Q14D22;Q9B	Charged mul	CHMP4A	6
1.58775	1.18948	-2.42314	-1.82741	0	9.51172	11	17	59	71	9.2257	8.62522	9.10023	9.19512	9.13373	8.31528	0.00554931	3.72E-06	P02452;H9C!	Collagen alpl	COL1A1	5
1.55483	1.28865	-1.9155	-1.21424	6.23E-146	8.78116	12	16	14	20	8.39582	7.70126	8.2978	8.55071	8.21903	8.27827	0.00488328	0.0165462	D2JYH5;P024	Collagen alpl	COL3A1	5
2.10802	1.74519	-1.96196	-1.20616	1.18E-17	7.86639	3	6	2	6	7.45082	6.68744	7.36862	7.65591	7.5499	6.99155	0.00087808	0.0671789	P05997;H7C:	Collagen alpl	COL5A2	5
-1.58066	-2.18639	-2.51837	-1.80935	2.71E-05	6.72244	1	1	1	1	6.57009	6.3982	6.08443	6.19357	6.11398	5.41752	0.0316123	0.0469887	C9J8T6;Q140	Cytochrome	COX17	2
2.77093	2.27828	-4.21982	-3.4313	1.41E-21	7.74495	1	4	1	2	6.8779	5.06513	6.87117	7.68155	7.67391	5.92309	0.00169997	2.04E-07	A4D1M3;Q9(	Carboxypept	CPA4	8
2.29563	1.8143	-4.66863	-4.01059	2.33E-134	8.99599	11	8	30	20	8.77788	8.0187	8.6948	8.59238	8.56336	7.40286	5.46E-05	6.85E-15	P29373;Q5S\	Cellular retin	CRABP2	5
2.55144	2.2158	-1.90136	-1.21223	4.29E-06	6.87015	1	2	1	2	6.57726	5.55141	6.53429	6.56081	6.44837	5.91896	0.00219997	0.17266	Q9NZV1;B4D	Cysteine-rich	CRIM1	3
2.4522	1.88881	-3.26701	-2.58294	4.00E-39	8.82904	4	4	10	12	8.43072	7.59777	8.36173	8.60743	8.52916	7.8247	6.06E-05	4.77E-07	Q96CG8;G3V	Collagen trip	CTHRC1	4
1.94343	1.45586	-1.96607	-1.37142	6.26E-10	7.26274	2	1	2	1	7.0593	6.37694	6.95812	6.83563	6.73799	6.1396	0.018568	0.126087	Q9NTM9;Q5	Copper hom	CUTC;RP11-4	3
1.67911	1.41868	-2.41519	-1.92994	1.00E-19	7.52304	5	3	4	1	7.4208	6.65859	7.33836	6.8448	6.82955	5.3827	0.02137	0.0346726	Q5T8F1;Q59	Death-associ	DAPK1	6
3.15903	2.64745	-1.619	-1.17251	3.15E-27	9.20868	3	1	4	1	9.2082	7.38707	9.20162	6.2447	6.1117	5.66596	3.29E-10	0.186076	Q58WW2;Q	DDB1- and C	DCAF6;IQWD	3
2.83788	2.25396	-2.92128	-2.39585	7.72E-09	7.35866	1	2	1	3	6.67043	5.67817	6.6238	7.25902	7.20483	6.32832	0.00188068	0.00017803	Q96PD2;B7Z	Discoidin, CU	DCBLD2	2
1.67021	1.35541	-2.10843	-1.54768	2.19E-138	8.35593	17	22	14	21	8.09219	7.42606	7.98668	8.01406	7.90495	7.36082	0.00519926	0.00304045	Q96BY6;B3F	Dedicator of	DOCK10;DOC	14
1.9871	1.53107	-3.2125	-2.58424	0.0011537	6.92005	1	1	1	1	6.51915	5.74767	6.43862	6.70017	6.66645	5.57344	0.0260902	0.00519812	Q9NRW4	Dual specifici	DUSP22	1
2.42315	1.83992	-2.44136	-1.59275	0	8.76288	7	10	10	14	8.43311	7.57646	8.36806	8.4888	8.4176	7.66842	9.12E-05	0.0017702	Q9NZN3;B4D	EH domain-c	EHD3	2
1.44932	1.08862	-1.83862	-1.18197	1.69E-115	8.83994	5	4	34	36	8.63843	8.05331	8.50769	8.40958	8.2917	7.78561	0.0156881	0.0195734	Q9HC35;B5M	Echinoderm	EML4	7
NaN	NaN	-3.01952	-2.46522	3.63E-90	7.27967	0	3	0	2	NaN	NaN	NaN	7.27967	7.23371	6.28165	1	0.000116	B1AHL2;B4DUV1;B1AHL4;		FBLN1	11
2.03298	1.76507	-2.11166	-1.66004	6.79E-34	7.66539	3	4	3	2	7.49105	6.7616	7.40144	7.18475	7.14485	6.12798	0.00077501	0.00872761	Q4L180;C9JN	Filamin A-int	FILIP1L	3
2.39259	1.89619	-2.30579	-1.64079	1.57E-71	8.42334	7	9	7	11	8.17246	7.48959	8.0714	8.06562	7.96939	7.36391	2.40E-05	0.00170012	Q8NF12;Q13	Calcyphosin	FLJ00390;CA	3
1.94849	1.53262	-3.27286	-2.68529	1.29E-102	8.0887	7	9	5	9	7.89252	7.12672	7.81082	7.64917	7.57094	6.86623	0.00184928	4.75E-05	Q6ZNI9;H0Y	Unconventio	FLJ00395;MY	8
1.91972	1.41792	-1.9578	-1.21961	7.24E-33	8.41427	6	10	12	11	7.95766	7.26224	7.85985	8.22755	8.12927	7.53394	0.00364971	0.0183846	Q12841;A8K	Follistatin-re	FSTL1	7
1.60051	1.32705	-1.86912	-1.27638	3.98E-23	7.20099	2	1	4	1	7.13994	6.47502	7.03411	6.31867	6.21809	5.6341	0.0298927	0.152544	Q86V85	Integral mem	GPR180	1
-1.54932	-2.09339	-6.11649	-5.41261	3.75E-20	8.19772	2	1	3	1	7.44846	7.31706	6.86523	8.1125	8.10745	6.17563	0.00475431	1.46E-23	P26583;Q5U	High mobility	HMGB2	3
1.67911	1.24513	-1.60007	-0.864596	0	9.34147	12	17	23	25	9.03926	8.37053	8.93444	9.04163	8.9144	8.44643	0.00486439	0.0581935	P54652;B3K	Heat shock-r	HSPA2	2
-1.49716	-1.99666	-3.84801	-3.12759	2.68E-203	8.3069	1	1	1	1	7.99578	7.83775	7.48011	8.01574	7.97867	6.92858	0.00456946	3.12E-09	E7ESP4;E7EMF1;E9PB77		ITGA2	3
1.76333	1.45275	-1.78433	-1.16576	5.92E-174	9.20583	18	18	29	32	8.95275	8.34036	8.8312	8.85088	8.73343	8.22554	0.00111177	0.0102942	P26006;B4E	Integrin alph	ITGA3	7
1.87606	1.50757	-1.97256	-1.33076	1.22E-212	8.91666	14	18	26	23	8.72009	8.06315	8.612	8.4778	8.33433	7.92702	0.00115089	0.00876077	P05106;B4D	Integrin beta	ITGB3	11
-3.02938	-3.35004	-4.00666	-3.36943	7.55E-255	7.39023	0	0	2	1	7.35769	7.32461	6.22287	6.24886	6.235	4.74637	6.95E-06	0.0003023	H6VRG2;B4DL32;H0YI76;H		KRT1	4
2.85084	2.25988	-2.51911	-1.86828	2.53E-08	6.85424	1	2	1	2	6.44163	5.57737	6.37782	6.64189	6.58429	5.73608	0.00183517	0.0405739	Q9BT23	LIM domain-	LIMD2	1
1.67645	1.2506	-2.13397	-1.41001	6.50E-22	8.33457	5	7	6	10	7.7049	6.97585	7.61519	8.21848	8.11787	7.53404	0.0137057	0.0068303	P51884;Q53	Lumican	LUM	3
1.56686	1.19415	-1.81345	-1.18164	0	9.32952	16	20	28	34	9.06251	8.44419	8.94288	8.99158	8.88576	8.3265	0.00679594	0.00928975	Q9UNF1;H7E	Melanoma-a	MAGED2	21
-2.18167	-2.30187	-1.72275	-0.830614	5.45E-15	7.59589	2	1	3	1	7.4128	7.32976	6.65333	7.13245	6.99914	6.55461	0.00272581	0.175879	Q14566;Q4Z	DNA replicati	MCM6	3
1.53595	0.949273	-1.99074	-1.30893	1.97E-177	8.90264	6	11	12	15	8.32836	7.79723	8.17696	8.76803	8.6707	8.07074	0.029587	0.0039136	B3KRC2;F5H	Monoglyceri	MGLL	12
1.6352	1.16524	-1.60112	-0.822069	9.78E-182	8.83834	11	9	13	10	8.54388	7.95881	8.41313	8.53064	8.4101	7.9151	0.0102067	0.100874	Q9BV20	Methylthiori	MRI1	1
-1.70792	-2.09634	-2.10024	-1.48173	0	8.1805	1	1	2	2	7.90988	7.77479	7.33694	7.84678	7.74083	7.18219	0.00276256	0.00451143	D3DTH7		MYO1C	1
-1.35638	-1.6765	1.63483	2.35282	5.69E-09	6.59832	2	3	2	2	6.32934	6.18887	5.77075	6.26269	5.63341	6.14647	0.116031	0.0134001	B4DQP1;E7E	NADH dehyd	NDUFA13	5
1.80095	1.46148	-1.83331	-1.12828	5.36E-58	7.69407	4	3	4	3	7.42719	6.79281	7.31252	7.35597	7.23565	6.73972	0.018174	0.0704551	Q9C002;H0Y	Normal muc	NMES1;C15o	2

-1.41074	-1.77448	1.81824	2.38225	7.70E-28	7.71423	5	3	6	3	7.64488	7.50598	7.08221	6.88326	6.1813	6.7871	0.0184398	0.0122381	Q13423;Q2T	NAD(P) trans	NNT	9
1.51485	1.29748	-2.01526	-1.37778	1.24E-17	7.16587	2	4	1	4	6.4442	5.82379	6.32521	7.07445	6.98452	6.34641	0.052458	0.0284737	Q13219;B4D	Pappalysin-1	PAPPA	6
-1.40354	-1.71227	-1.82941	-1.4079	5.32E-07	7.13906	2	3	2	2	6.88335	6.67619	6.46241	6.78743	6.67758	6.13669	0.106926	0.116938	Q3YEC7;G8JL	Rab-like prot	PARF;C9orf8	10
2.61565	2.08257	-2.34512	-1.57248	0	9.65351	0	0	42	68	9.27114	8.47614	9.19526	9.42095	9.32775	8.70679	8.48E-07	7.72E-05	B4DRT3;E7EU	Pyruvate kin	PKM2	3
NaN	NaN	-2.93832	-2.11866	4.13E-219	7.41868	0	1	0	2	NaN	NaN	NaN	7.41868	7.36524	6.48237	1	0.00132424	E7ETU9;B4DHG3;B3KWS3;		PLOD2	6
1.82244	1.25635	-2.42973	-1.80591	8.57E-300	8.78436	8	9	13	11	8.46112	7.82859	8.3459	8.50444	8.42886	7.70788	0.00595468	0.00040434	B2R6X6;P304	Peptidyl-prol	PPIF	13
2.55159	2.20402	-2.89218	-2.19621	1.07E-53	8.34974	6	9	10	16	7.9222	6.99217	7.86792	8.14656	8.08714	7.25324	1.55E-05	3.49E-05	P26022	Pentraxin-rel	PTX3	1
1.69737	1.27471	-1.71317	-1.01162	1.55E-63	8.56378	3	4	15	15	8.45643	7.80347	8.34721	7.90421	7.76884	7.33201	0.00532229	0.0501373	Q92930;H0YI	Ras-related p	RAB8B	10
2.47902	2.01489	-3.0308	-2.37452	6.72E-48	8.14891	2	5	2	4	7.23277	6.4755	7.14931	8.09276	8.03687	7.17464	0.00165224	8.05E-06	A8K3J8;P550	GTP-binding	RRAD	5
1.37995	0.823749	-2.22553	-1.57192	3.53E-82	9.46984	6	5	66	53	9.23454	8.69557	9.08636	9.09132	9.00711	8.33754	0.0584186	0.0005176	D3DV26;P60	Protein S100	S100A10	3
1.56866	1.1036	-1.95137	-1.23416	1.06E-07	6.91916	2	1	2	1	6.85284	6.26309	6.72372	6.0703	5.96909	5.38803	0.0886865	0.165572	P29034;Q5RI	Protein S100	S100A2	2
1.69995	1.14841	-2.09486	-1.45918	1.88E-40	7.88545	6	5	6	6	7.64803	7.04187	7.52445	7.50986	7.41469	6.80391	0.0222084	0.0268856	P33764	Protein S100	S100A3	1
1.83435	1.47566	-2.50611	-1.83259	2.04E-224	9.46046	2	2	37	43	8.8674	8.12898	8.77982	9.33248	9.24878	8.57624	0.00093411	3.48E-06	B2R7Y0;H7BYS2;H7C004;E		SERPINB10	4
2.74966	2.17702	-3.35868	-2.65608	1.87E-169	8.14882	2	1	4	2	7.91954	7.04021	7.85804	7.76178	7.72702	6.64779	1.92E-05	4.62E-07	P05120;E7EP	Plasminogen	SERPINB2	3
2.31136	1.79086	-2.21632	-1.49387	9.21E-29	8.32595	7	6	8	7	7.96501	7.26724	7.8678	8.07755	7.95839	7.45761	0.00034582	0.00419985	P05121;F8W	Plasminogen	SERPINE1	7
1.50294	0.987757	-2.43893	-1.48201	1.34E-184	8.72054	11	18	17	20	8.20542	7.60987	8.07828	8.56227	8.48048	7.79689	0.0238949	0.00358171	Q9H788;H0Y	SH2 domain-	SH2D4A	2
-1.82337	-2.19106	2.06533	2.77964	1.28E-18	7.90064	3	5	3	5	7.10195	7.01812	6.34633	7.82545	7.28344	7.6785	0.00452962	3.99E-05	D9HTE9;Q6L	Tricarboxylat	SLC25A1	4
1.70867	1.40746	-1.65676	-1.18761	5.37E-06	6.97997	1	1	1	1	6.79385	6.1002	6.6956	6.52222	6.39291	5.93303	0.0380997	0.180887	B7ZLQ5;P283	Probable glol	SMARCA1	7
1.67224	1.20069	-2.01841	-1.24646	0	9.49624	19	24	30	43	9.0844	8.3987	8.98408	9.28341	9.16679	8.65549	0.00651447	0.00199033	P52788;B4DI	Spermine syr	SMS	3
1.43563	0.91311	-2.16961	-1.495	4.22E-10	7.33157	3	2	4	2	7.14476	6.58583	7.00445	6.87512	6.77938	6.17143	0.113026	0.0971982	P37840;H6U	Alpha-synucl	SNCA	6
2.93172	2.45375	-3.74101	-2.83516	1.26E-12	7.02284	3	3	3	3	6.80456	5.65401	6.7727	6.61954	6.55229	5.77626	0.00080037	0.00224189	O76070;Q6FI	Gamma-synu	SNCG	4
1.63338	1.32343	-1.80682	-1.18187	3.57E-06	7.37146	2	1	3	1	7.19769	6.54683	7.08789	6.8896	6.82061	6.05664	0.0302831	0.182846	D2JYI1;A3QN	TGF-beta rec	TGFBR2	5
1.4197	1.00813	-3.20051	-2.67355	0.00028262	6.86025	1	1	1	1	6.56071	5.98134	6.42792	6.55775	6.52842	5.3726	0.112813	0.00388153	Q96HP8;H7C	Transmembr	TMEM176A	4
-1.58701	-2.04258	1.98939	2.6893	4.54E-09	7.79993	2	2	3	2	7.63155	7.47387	7.11508	7.30698	6.61731	7.20772	0.00597974	0.00024261	Q99785;E9PI	Tumor protei	TP53I11	13
1.49978	0.965175	-2.01392	-1.36702	4.00E-233	9.25249	16	24	25	45	8.66165	8.10332	8.5211	9.12375	9.0089	8.48994	0.0299951	0.00257331	Q16222;B1AI	UDP-N-acety	UAP1	2
-1.52891	-1.65381	-1.9143	-1.2137	1.38E-06	7.03104	1	1	1	2	6.20874	6.06187	5.66644	6.96019	6.84334	6.3329	0.122118	0.172177	P63146;H0Y	Ubiquitin-cor	UBE2B	3
1.72583	1.49093	-3.07534	-2.31058	0.00046673	7.19218	1	1	1	1	7.06002	6.38714	6.95633	6.61111	6.55672	5.68195	0.0162255	0.0121038	P17029;B3KN	Zinc finger pr	ZKSCAN1	5

Table S2. List of reagents used in this study (Related to Figures 1-7)

Primer sequences for qPCR analyses used in this study

**HUMAN**		
**Target**	**Forward primer**	**Reverse primer**
RPS14	CTGCGAGTGCTGTCAGAGG	TCACCGCCCTACACATCAAACT
INK4A	CGGTCGGAGGCCGATCCAG	GCGCCGTGGAGCAGCAGCAGCT
TP53	TGGCCATCTACAAGCAGTCA	GGTACAGTCAGAGCCAACCT
CBX7	AACTCCATCACCGTCACCTT	CCCCAACCCATCCCTATCTC
NDUFA13	CTACGGGCACTGGAGCATAA	AGCGGGTTGTGTGGAACA
S100A3	CAGATTGGTAAACACCCGAAC	ACAAAGTCCACCTCGCAGTC
TGFBR2	CGGCTCCCTAAACACTACCA	TATGTCACCCACTCCCTGCT
AIM1	TCCCCAGAAAGTGAAGGAAA	TGTTGGAAGAGCAGCGTATG
DUSP22	GAAAGGGGAGTGTGGCTGTA	GCGGCTGTGAAGAAAGAACA
RAB8B	GCACATCAGTGTTAGCCTTTCC	TGAACCAGACCAAATACCCTTT
NNT	GATACTGGGTTTGGGCATTG	CCCTGAGAAGTTGTGGAAGG
ITGB3	GGGGTAGGTTGGGAGAATGT	TCTGGGACAAAGGCTAAGGA
SMS	GCACAGCGAAGACTGCTAAA	GGGGAAAGAAACACCATCAA
SNCA	TTCTGGGGCATAGTCATTTCT	TTCCTCCTTCCTTCCTCACC
ZKSCAN1	CCATTTCCCCCTTTTGTTTC	TGCGTGTGCTTTTCTCTTGT
ITGA2	GGATTTGTTTGGCTGACTGG	GATAACTTTGGACCGCTGGA
EHD3	CCCACCACAGACTCCTTCAT	GCTCTCCAGCACAGGGTTAG
MAGED2	CAGGCATACTGGGAACGACT	GCTATTGGGGACTCTGGCATA
S100A2	AAGAGGGCGACAAGTTCAAG	TGATGAGTGCCAGGAAAACA
ARMC8	TCCTCTCCACTCGTCCTCAT	GTTGTCCCATCCGCTATGTT
DAPK1	GAAGCAAGGGGGTGATGTAG	CCACAGACAACGGAATGAGA
SH2D4A	ATGCCCTGTCCTATCTGTCG	TGGAGGCTGTCACTCAAACA
FILIP1L	AAACGCCTCCATAACACCAG	AACCAGTCACAGCCAAAACC
GRO alpha	GAAAGCTTGCCTCAATCCTG	CACCAGTGAGCTTCCTCCTC
GCP2	AGAGCTGCGTTGCACTTGTT	GCAGTTTACCAATCGTTTTGGGG
CCL2	AGCTCGCACTCTCGCCTCCAG	GGCATTGATTGCATCTGGCTGAGC
IL-1 beta	TGCACGCTCCGGGACTCACA	CATGGAGAACACCACTTGTTGCTCC
IL-8	GAGTGGACCACACTGCGCCA	TCCACAACCCTCTGCACCCAGT
IL-6	CCAGGAGCCCAGCTATGAAC	CCCAGGGAGAAGGCAACTG
IL-11	CCTGGCTCTTCCCCATCTAG	CAGCTCTCAGACAAATCGCC
TGFB1	TAC TAC GCC AAG GAG GTC AC	GCT GAG GTA TCG CCA GGA AT
TGFB2	TTT GGA AGT TTG TGT TCT GTT TG	TGT TGT TGT TGT CGT TGT TCA C
TGFB3	ACA CAC AAG CAA CAA ACC TCA C	TCC TCT AAC CAA ACC CAC ACT T
SMAD2	GCAAAAAGGTTCACACTAAAGGA	AGCAAAGGTTGAGGAAGGAGATA
SMAD3	TGATGTTAGAGGGAGATGGAGAG	GAGGAAGTGAGGGGTTTTGTATT
SMAD4	ACCCCAGCTCTGTTAGCCCCA	TGGCAGGCTGACTTGTGGAAGC
TGFBR1 (ALK5)	TCTGCCACAACCGCACTGTCA	GGTAAACCTGAGCCAGAACCTGACG
LTBP1	GAG TGC TGC TGT CTG TAT GGA G	AAACGGTCTTGGATGAAGTAGG
LTBP3	GGA GGA GAA GAG CCT GTG TTT	GTG GGA GGT GAG AAT GTG GTA T
CDKN2B (p15INK4B)	ACCAGATAGCAGAGGGGTAAGAG	GTGTGTGTGTGTGTGTGTGAAAG
CDKN1B (p27KIP)	CCAGTCCATTTGATCAGCGG	ACATCTTTCTCCCGGGTCTG
MMP1	CAAATGCAGGAATTCTTTGGGC	GTAGGTCAGATGTGTTTGCTCC
MMP9	AACTTTGACAGCGACAAGAAGT	ATTCACGTCGTCCTTATGCAAG
ITGB1	TGTGGTTGCTGGAATTGTTCTT	ATTCAGTGTTGTGGGATTTGCA
ITGB2	ATGTGGATGAGAGCCGAGAG	ACTGGGACTTGAGCTTCTCC
ITGB4	ACTACACCCTCACTGCAGAC	TCTGGCTTGCTCCTTGATGA
ITGB5	AACCAGAGCGTGTACCAGAA	AGGAGAAGTTGTCGCACTCA
ITGB6	GAAGGGGTGACTGCTACTGT	TGCACACACATTCACCACAG
ITGB7	AAGTTGGGCGGCATTTTCAT	CCCCAACTGCAGACTTAGGA
ITGB8	CCCAGAATCACTCCAACCCT	GTGAACCCTAATTGCGCCAT
**MOUSE**		
Ink4a	GTGTGCATGACGTGCGGG	GCAGTTCGAATCTGCACCGTAG
Rps14	GACCAAGACCCCTGGACCT	CCCCTTTTCTTCGAGTGCTA
Itgb3	AACCACTACTCTGCCTCCAC	ACTGTGGTCCCAGGAATGAG
Tp53	AAACGCTTCGAGATGTTCCG	GTAGACTGGCCCTTCTTGGT
Cdkn1a (p21CIP)	TCCCGACTCTTGACATTGCT	TGCAGAAGGGGAAGTATGGG
Cxcl1	CTGGGATTCACCTCAAGAACATC	CAGGGTCAAGGCAAGCCTC
Inhibin A	GATCATCACCTTTGCCGAGT	TGGTCCTGGTTCTGTTAGCC
Il-1 alpha	CGCTTGAGTCGGCAAAGAAAT	TGGCAGAACTGTAGTCTTCGT
Il1- beta	TGCCACCTTTTGACAGTGATG	TGATGTGCTGCTGCGAGATT
Il-6	TGATTGTATGAACAACGATGATGC	GGACTCTGGCTTTGTCTTTCTTGT
Cbx7	TGCGGAAGGGCAAAGTTGAAT	ACAAGGCGAGGGTCCAAGA

Primer sequences for ChIP-qPCR analyses used in this study

Target	Forward primer	Reverse primer
ARF	GTGGGTCCCAGTCTGCAGTTA	CCTTTGGCACCAGAGGTGAG
ACTB	CCGTTCCGAAAGTTGCCTT	CGCCGCCGGGTTTTATA
INK4A	ACCCCGATTCAATTTGGCAG	AAAAAGAAATCCGCCCCCG
ITGB3 TSS	CTGAAGACACGTGCCCAAGG	TCCGTCTCTAACCTGGAAGTCC
ITGB3 Coding	GTGCAGTGGTGTGATCCCTG	AGCATTTTGGGAAGCCGAGG
SNCA	GGAGTCGGAGTTGTGGAGAA	GGGACAAGTACTCACCTCCC
ZKSCAN	AAAAGTAAATCTGGCCGGGC	TCCCAGGTTCAAGCGATTCT
S100A2	CCCATCCTTCCAGACACCTT	TGAGAGAGAAGCAACCTGGG
ITGA2	TGCTGGAAATTTGTGGCCAA	TGAGAGCCCATACATGCACT
ARMC8	AAACTCCCAGTGCCTGTCTT	ATGGGGCAGAACATAACCCT
DUSP22	CCGCTGACTTGTTGACACTG	TGTTCATCCCATTCCCCATG
S100A3	GAAGGGGACAGTGGAAGTGG	AAGTTGGGGTTCATCTCACC
TGFBR2	GCGCTGAGTTGAAGTTGAGT	AGATGTGCGGGCCAGATG
SH2D4A	GTCTCCTTCCTTCCAGCCTT	ATCTGCAGATCTGGGCCTTT
AIM1	CGGTCGTGATTACTCCCAGA	CCCGCGGAGATTTCACTTTC
FILIP1L	CAAAGGTGGAAGGTGCACTC	TCCCAAATCCCATCCTTCCC
RAB8B	CTCTCCACCGCCTCCTCT	GGTGGAGATGAAGGTGGTGT
DAPK1	CTTCGGAGTGTGAGGAGGAC	GGGAACACAGCTAGGGAGTG

RNAi sequences used in this study

siRNA	
**Target**	**Sequence**
siTP53_7	CAGCATCTTATCCGAGTGGAA
siITGB3_3	CTCTCCTGATGTTAGCACTTAA
siITGB3_4	CAAGCTGAACCTAATAGCCAT
siITGB3_5	CACGTGTGGCCTGTTCTTCTA
siTGFBR2_2	TCGCTTTGCTGAGGTCTATAA
siTGFBR2_7	TCGGTTAATAACGACATGATA
**shRNA**	
**Target**	**Sequence**
TP53	GTAGATTACCACTGGAGTC
CBX7	CGGAAGGGTAAAGTCGAGT
ITGB3	GATGCAGTGAATTGTACCTAT

Antibodies used in this study

Target	Catalogue n. - Clone	Application	Concentration
CBX7	Ab21873	ChIP, WB	1/200
CBX8	A300-882A	ChIP	1/200
RING1B	Ab3832	ChIP	1/200
IgG	Ab18443 - MOPC21	ChIP	1/200
p16INK4A	sc-56330 - JC-8	WB	1/200
β-tubulin	sc-9104	WB	1/500
ITGB3 or β3	Ab179473 - ERP17507	WB, IF	1/200
β-Actin	sc-47778	WB	1/500
p21CIP	Ab109520	IF	1/200
BrdU	A21303	IF	1/200
αvβ3	LM609	blocking Ab	10μg/ml
p53	sc-126 - DOI	IF	1/200
Pan TGFB 1-3	AB-100-NA	blocking Ab/WB	10μg/ml
IgG blocking antibodies	ab18413	blocking Ab	10μg/ml
pST/Q	9607	IF	1/200
Ki67	ab15580	IF	1/500
SMAD2/3	5678S	IF	1/200
CXCL6/IL-6	AB-206-NA	WB/IF	1:250/1:200
CXCL8/IL-8	6217	WB/IF	1:500/1:200

Inhibitors used in this study

Name	Target	Catalogue n.	Concentration
PD98059	MEK1/2	99005	40 μM
SB202190	p38MAPK	sc-222294	20 μM
Torin-2	mTOR	14185	100 nM
Tgf-B Ri Kinase Inhibitor I	Tgf-B Ri Kinase Inhibitor I	61645	4 μM
Vegfr-2/Flt3/C-Kit	Vegfr-2/Flt3/C-Kit	676500	8 μM
Etoposide	DNA damage	341205	100 μM
PD0332991 (Palbociclib)	CDK4/6	A8316	200 nM
GSK429286A	ROCK1/2	Ab1466581	150 nM
ILK Inhibitor, Cpd22	ILK	40733	50 nM
Cilengitide	αvβ3/αvβ5	A12372	50 nM
